# Supplementary material for: Prognostic factors for favorable outcomes after veno-venous extracorporeal membrane oxygenation in critical care patients with COVID-19
Source: PLoS One. 2023 Jan 20;18(1):e0280502. doi: 10.1371/journal.pone.0280502 (PMC9858373; doi:10.1371/journal.pone.0280502)
Supplement: S1 Table — (DOCX) [file pone.0280502.s003.docx]

| **1 Baseline and demographic data** | | | |
| --- | --- | --- | --- |
| Age at the start of VV ECMO | metric | numeric | years |
| Sex | categorical | 0 = men  1 = women |  |
| Body mass index, BMI | metric | numeric | kg/m² |
| Previous diseases   - cardiovascular - pulmonary - renal - endocrinological - gastrointestinal - metabolic - dermatological - neurological - orthopedic - gynecological/urological - malignant disease - obesity - noxes - infection - other - immunsuppression | categorical | 0 = no  1 = yes |  |
| AKI before the start of VVECMO | categorical | 0 = no  1 = yes |  |

| **2 Initial values before the start of VV ECMO^[[1]](#footnote-1)^** | | | |
| --- | --- | --- | --- |
| pH | metric | numeric |  |
| Bicarbonate ion HCO_3_^-^ | metric | numeric | mmol/L |
| Base excess BE | metric | numeric | mmol/L |
| Partial pressure of oxygen paO_2_ | metric | numeric | mmHg |
| Partial pressure of carbon dioxide paCO_2_ | metric | numeric | mmHg |
| Hemoglobin Hb | metric | numeric | g/dL |
| Lactate | metric | numeric | mg/mL |
| Creatinine | metric | numeric | mg/dL |
| Urea | metric | numeric | mg/dL |
| Aspartate transaminase AST | metric | numeric | U/L |
| Alanine transaminase ALT | metric | numeric | U/L |
| International Normalized Ratio INR | metric | numeric |  |
| Lactate dehydrogenase LDH | metric | numeric | U/L |
| C-reactive protein CRP | metric | numeric | mg/L |
| Procalcitonin PCT | metric | numeric | ng/mL |
| White blood cells WBC | metric | numeric | /nL |
| D-dimers | metric | numeric | mg/L |
| Platelets | metric | numeric | /nL |
| Interleukin 6 IL6 | metric | numeric | pg/mL |
| Norepinephrine | metric | numeric | mg/h |
| Mean arterial pressure MAP | metric | numeric | mmHg |
| Fraction of inspired oxygen FiO_2_ | metric | numeric | % |
| Oxygenation ratio (Horovitz) | metric | numeric |  |
| Positive endexpiratory pressure PEEP | metric | numeric | mmHg |
| Tidal volume VT | metric | numeric | mL |
| Mean airway pressure Pmean | metric | numeric | mmHg |

| **3 Basic data ECMO** | | | |
| --- | --- | --- | --- |
| Period symptom onset until the start of VV ECMO | metric | numeric | days |
| Period admission ICU until the start of VV ECMO | metric | numeric | days |
| Period intubation until the start of VV ECMO | metric | numeric | days |
| Start of VV-ECMO at an external hospital | categorical | 0 = no  1 = yes |  |
| Duration of VV ECMO | metric | numeric | days |

| **4 Laboratory blood diagnostics^[[2]](#footnote-2)^** | | | |
| --- | --- | --- | --- |
| pH (average, minimum, maximum) | metric | numeric |  |
| Bicarbonate ion HCO_3_^-^ (average, minimum, maximum) | metric | numeric | mmol/L |
| Base excess BE (average, minimum, maximum) | metric | numeric | mmol/L |
| Cloride Cl^-^ (average, minimum, maximum) | metric | numeric | mmol/L |
| Partial pressure of oxygen paO_2_ (average, minimum) | metric | numeric | mmHg |
| Partial pressure of carbon dioxide paCO_2_ (average, minimum, maximum) | metric | numeric | mmHg |
| Hemoglobin Hb (average, minimum, maximum) | metric | numeric | g/dL |
| Lactate (average, maximum) | metric | numeric | mg/mL |
| Troponin (maximum) | metric | numeric | ng/L |
| Glomerular filtration rate GFR (minimum) | metric | numeric | mL/min/1.73m^2^ |
| Creatinine (maximum) | metric | numeric | mg/dL |
| Urea (maximum) | metric | numeric | mg/dL |
| Aspartate transaminase AST (maximum) | metric | numeric | U/L |
| Alanine transaminase ALT (maximum) | metric | numeric | U/L |
| Lactate dehydrogenase LDH (maximum) | metric | numeric | U/L |
| C-reactive protein CRP (maximum) | metric | numeric | mg/L |
| Procalcitonin PCT (maximum) | metric | numeric | ng/mL |
| White blood cells WBC (maximum) | metric | numeric | /nL |
| Ferritin (maximum) | metric | numeric | ng/mL |
| Lymphocytes (absolute value) (maximum) | metric | numeric | x10ᶾ/μL |
| Interleukin 6 IL6 (maximum) | metric | numeric | pg/mL |
| International Normalized Ratio INR (average) | metric | numeric |  |
| D-dimers (maximum) | metric | numeric | mg/L |
| Platelets (average) | metric | numeric | /nL |

| **5 Dosage of catecholamines and circulatory parameters^[[3]](#footnote-3)^** | | | |
| --- | --- | --- | --- |
| Norepinephrine (average, maximum) | metric | numeric | mg/h |
| Mean arterial pressure MAP (average, minimum, maximum) | metric | numeric | mmHg |
| Heartrate HR (average, minimum, maximum) | metric | numeric | bpm |

| **6 VV ECMO parameters^3^** | | | |
| --- | --- | --- | --- |
| Pump flow rate (average, minimum, maximum) | metric | numeric | L/min |
| Sweep gas flow rate (average, minimum, maximum) | metric | numeric | L/min |

| **7 Ventilation therapy^3^** | | | |
| --- | --- | --- | --- |
| Fraction of inspired oxygen FiO_2_ (average, minimum, maximum) | metric | numeric | % |
| Oxygenation ratio (Horovitz) (average, minimum, maximum) | metric | numeric |  |
| Positive endexpiratory pressure PEEP (average) | metric | numeric | mmHg |
| Respiratory minute volume (RMV) (average) | metric | numeric | mL |
| Tidal volume (VT) (average) | metric | numeric | mL |
| Peak inspiratory airway pressure Ppeak (average) | metric | numeric | mmHg |

| **8 Fever, RRT and ICU scores^3^** | | | |
| --- | --- | --- | --- |
| Therapeutic Intervention Scoring System TISS | metric | numeric |  |
| Simplified Acute Physiology Score SAPS | metric | numeric |  |
| Fever, daily peak value | categorical | 0 = daily peak value < 38°C  1 = daily peak value ≥ 38°C |  |
| Renal replacement therapy RRT^A^ | categorical | 0 = no RRT  1 = RRT |  |

| **9 Anticoagulation and antithrombotic mediation^[[4]](#footnote-4)^** | | | |
| --- | --- | --- | --- |
| Unfractionated heparin, low molecular weight heparin or argatoban | categorical | 0 = not received  1 = received |  |
| Acetylsalicylic acid | categorical | 0 = not received  1 = received |  |

| **10 Description of fatal cases** | | | |
| --- | --- | --- | --- |
| Time of death after the start of VV ECMO | metric |  |  |
| Died while being on VV ECMO | categorical | 0 = no  1 = yes |  |
| Cause of death | categorical | 1 = MOF  2 = respiratory failure  3 = cerebral bleeding  4 = fatal bleeding  5 = cardiac failure  6 = cerebral ischemia  7 = intestinal ischemia |  |

1. Last measured value before the start of VV ECMO [↑](#footnote-ref-1)
2. Each calculated for the time periods 0-24 h, 48-72 h, 96-120 h, and 216-240 h after the start VV ECMO [↑](#footnote-ref-2)
3. Each calculated for the time periods 0-24 h, 48-72 h, 96-120 h, and 216-240 h after the start of VV ECMO [↑](#footnote-ref-3)
4. Each calculated for the time periods 0-24 h, 48-72 h, 96-120 h, and 216-240 h after the start of VV ECMO [↑](#footnote-ref-4)
