# Supplementary material for: Prognostic factors for favorable outcomes after veno-venous extracorporeal membrane oxygenation in critical care patients with COVID-19
Source: PLoS One. 2023 Jan 20;18(1):e0280502. doi: 10.1371/journal.pone.0280502 (PMC9858373; doi:10.1371/journal.pone.0280502)
Supplement: S2 Table — (DOCX) [file pone.0280502.s004.docx]

|  | Survivors  SD  min  max | | | | Non-survivors | | | | 95%-CI of MD | | | p |
| --- | --- | --- | --- | --- | --- | --- | --- | --- | --- | --- | --- | --- |
|  | mean | SD | min | max | mean | SD | min | max | mean | min | max |  |
| pH av 1 | 7.46 | 0.05 | 7.30 | 7.57 | 7.46 | 0.06 | 7.34 | 7.62 | 0.00 | -0.02 | 0.02 | 9.661E-01 |
| pH av 3 | 7.45 | 0.04 | 7.33 | 7.54 | 7.45 | 0.04 | 7.36 | 7.55 | 0.00 | -0.01 | 0.02 | 4.958E-01 |
| pH av 5 | 7.45 | 0.03 | 7.36 | 7.52 | 7.43 | 0.05 | 7.28 | 7.52 | 0.02 | 0.01 | 0.04 | 8.479E-03 |
| pH av 10 | 7.45 | 0.03 | 7.38 | 7.51 | 7.42 | 0.05 | 7.28 | 7.52 | 0.02 | 0.01 | 0.04 | 4.055E-02 |
| pH min 1 | 7.37 | 0.08 | 7.06 | 7.50 | 7.36 | 0.10 | 7.07 | 7.57 | 0.01 | -0.02 | 0.04 | 6.260E-01 |
| pH min 3 | 7.40 | 0.05 | 7.28 | 7.49 | 7.40 | 0.05 | 7.31 | 7.53 | 0.00 | -0.01 | 0.02 | 4.916E-01 |
| pH min 5 | 7.41 | 0.04 | 7.31 | 7.50 | 7.38 | 0.06 | 7.20 | 7.48 | 0.02 | 0.00 | 0.04 | 6.169E-02 |
| pH min 10 | 7.39 | 0.04 | 7.30 | 7.52 | 7.37 | 0.06 | 7.19 | 7.46 | 0.03 | 0.01 | 0.05 | 4.926E-02 |
| pH max 1 | 7.54 | 0.06 | 7.37 | 7.67 | 7.56 | 0.07 | 7.44 | 7.70 | -0.01 | -0.04 | 0.01 | 4.173E-01 |
| pH max 3 | 7.50 | 0.04 | 7.41 | 7.60 | 7.50 | 0.04 | 7.41 | 7.59 | 0.00 | -0.01 | 0.02 | 6.255E-01 |
| pH max 5 | 7.51 | 0.03 | 7.43 | 7.59 | 7.49 | 0.06 | 7.35 | 7.62 | 0.02 | 0.00 | 0.04 | 5.348E-02 |
| pH max 10 | 7.51 | 0.05 | 7.43 | 7.64 | 7.49 | 0.06 | 7.36 | 7.62 | 0.02 | 0.00 | 0.05 | 5.561E-02 |
| HCO_3_^-^ av 1 | 30.5 | 4.7 | 19.3 | 42.0 | 30.4 | 4.6 | 20.0 | 40.0 | 0.1 | -1.5 | 1.8 | 9.894E-01 |
| HCO_3_^-^ av 3 | 31.9 | 3.9 | 23.9 | 44.1 | 30.8 | 3.6 | 21.6 | 37.7 | 1.1 | -0.2 | 2.5 | 1.304E-01 |
| HCO_3_^-^ av 5 | 31.9 | 3.8 | 25.4 | 43.3 | 30.4 | 5.0 | 20.6 | 42.8 | 1.5 | -0.2 | 3.2 | 6.974E-02 |
| HCO_3_^-^ av 10 | 31.7 | 3.7 | 22.3 | 40.6 | 29.3 | 5.0 | 18.0 | 39.1 | 2.3 | 0.6 | 4.1 | 5.374E-03 |
| HCO_3_^-^ min 1 | 27.0 | 4.7 | 15.1 | 38.7 | 26.7 | 4.6 | 15.6 | 35.7 | 0.3 | -1.3 | 2.0 | 7.829E-01 |
| HCO_3_^-^ min 3 | 29.6 | 3.8 | 21.4 | 40.4 | 28.7 | 3.6 | 20.3 | 35.8 | 0.9 | -0.4 | 2.3 | 2.160E-01 |
| HCO_3_^-^ min 5 | 29.4 | 3.9 | 20.2 | 39.9 | 28.3 | 4.8 | 19.7 | 41.6 | 1.1 | -0.5 | 2.7 | 2.389E-01 |
| HCO_3_^-^ min 10 | 29.3 | 3.8 | 20.9 | 39.5 | 26.5 | 5.3 | 13.5 | 37.3 | 2.7 | 0.9 | 4.5 | 2.737E-03 |
| HCO_3_^-^ max 1 | 33.7 | 4.8 | 23.4 | 44.5 | 33.7 | 5.2 | 22.2 | 44.7 | 0.0 | -1.8 | 1.9 | 8.737E-01 |
| HCO_3_^-^ max 3 | 34.0 | 4.1 | 25.3 | 47.0 | 32.9 | 3.8 | 23.2 | 40.7 | 1.1 | -0.3 | 2.5 | 1.408E-01 |
| HCO_3_^-^ max 5 | 34.3 | 4.0 | 26.7 | 46.4 | 32.7 | 5.1 | 22.4 | 46.3 | 1.6 | -0.1 | 3.3 | 4.987E-02 |
| HCO_3_^-^ max 10 | 33.9 | 3.7 | 25.7 | 42.3 | 31.5 | 5.2 | 22.6 | 42.7 | 2.4 | 0.6 | 4.2 | 4.460E-03 |
| BE av 1 | 6.2 | 4.5 | -4.3 | 16.9 | 6.1 | 4.7 | -3.9 | 15.3 | 0.1 | -1.6 | 1.8 | 9.468E-01 |
| BE av 3 | 7.2 | 3.7 | -0.1 | 17.9 | 6.1 | 3.5 | -2.9 | 12.7 | 1.1 | -0.3 | 2.4 | 1.540E-01 |
| BE av 5 | 7.2 | 3.5 | 1.8 | 18.0 | 5.6 | 4.8 | -3.8 | 17.5 | 1.7 | 0.1 | 3.3 | 4.649E-02 |
| BE av 10 | 6.9 | 3.5 | -1.6 | 14.7 | 4.5 | 5.1 | -6.9 | 14.5 | 2.4 | 0.6 | 4.1 | 7.052E-03 |
| BE min 1 | 3.1 | 4.7 | -8.8 | 13.5 | 2.3 | 4.6 | -12.2 | 11.1 | 0.8 | -0.9 | 2.4 | 4.235E-01 |
| BE min 3 | 5.1 | 3.7 | -3.0 | 14.8 | 4.1 | 3.4 | -4.0 | 10.5 | 1.0 | -0.2 | 2.3 | 1.355E-01 |
| BE min 5 | 4.9 | 3.6 | -3.8 | 15.0 | 3.5 | 4.7 | -5.4 | 16.8 | 1.5 | -0.1 | 3.1 | 9.309E-02 |
| BE min 10 | 4.6 | 3.6 | -3.4 | 13.4 | 1.9 | 5.4 | -12.2 | 12.9 | 2.7 | 0.8 | 4.6 | 4.062E-03 |
| BE max 1 | 8.6 | 4.6 | 0.2 | 19.8 | 9.0 | 5.3 | -2.5 | 19.1 | -0.3 | -2.2 | 1.5 | 7.094E-01 |
| BE max 3 | 9.0 | 3.8 | 1.0 | 20.3 | 7.9 | 3.8 | -2.1 | 15.2 | 1.1 | -0.3 | 2.5 | 1.650E-01 |
| BE max 5 | 9.2 | 3.8 | 2.7 | 20.8 | 7.5 | 5.0 | -2.9 | 19.7 | 1.7 | 0.1 | 3.4 | 3.204E-02 |
| BE max 10 | 8.8 | 3.7 | -0.2 | 16.8 | 6.6 | 5.1 | -2.3 | 18.1 | 2.2 | 0.5 | 4.0 | 7.695E-03 |
| Cl^-^ av 1 | 107.9 | 5.2 | 96.5 | 121.8 | 107.4 | 5.1 | 94.8 | 122.6 | 0.6 | -1.3 | 2.4 | 5.144E-01 |
| Cl^-^ av 3 | 108.7 | 5.6 | 88.1 | 120.3 | 109.5 | 6.2 | 95.9 | 123.7 | -0.8 | -3.0 | 1.4 | 4.989E-01 |
| Cl^-^ av 5 | 107.0 | 6.0 | 86.8 | 119.8 | 108.8 | 7.7 | 91.4 | 131.2 | -1.8 | -4.4 | 0.8 | 2.774E-01 |
| Cl^-^ av 10 | 106.0 | 5.8 | 91.3 | 120.2 | 109.1 | 7.0 | 94.1 | 125.1 | -3.2 | -5.7 | -0.6 | 2.923E-02 |
| Cl^-^ min 1 | 104.8 | 5.4 | 94.0 | 119.0 | 104.1 | 5.8 | 91.0 | 120.0 | 0.7 | -1.4 | 2.7 | 5.040E-01 |
| Cl^-^ min 3 | 106.3 | 5.6 | 85.0 | 118.0 | 107.0 | 6.2 | 94.0 | 122.0 | -0.8 | -2.9 | 1.4 | 7.794E-01 |
| Cl^-^ min 5 | 104.1 | 6.2 | 84.0 | 117.0 | 106.4 | 7.8 | 89.0 | 129.0 | -2.4 | -5.0 | 0.3 | 2.053E-01 |
| Cl^-^ min 10 | 103.4 | 5.8 | 90.0 | 119.0 | 106.8 | 6.9 | 91.0 | 121.0 | -3.4 | -5.9 | -0.9 | 1.179E-02 |
| Cl^-^ max 1 | 111.5 | 5.4 | 99.0 | 126.0 | 111.6 | 4.6 | 100.0 | 126.0 | -0.1 | -1.9 | 1.6 | 8.543E-01 |
| Cl^-^ max 3 | 111.6 | 5.5 | 94.0 | 123.0 | 112.2 | 6.2 | 99.0 | 126.0 | -0.6 | -2.8 | 1.6 | 6.848E-01 |
| Cl^-^ max 5 | 110.4 | 6.0 | 91.0 | 123.0 | 112.0 | 7.5 | 96.0 | 132.0 | -1.6 | -4.1 | 1.0 | 3.708E-01 |
| Cl^-^ max 10 | 108.7 | 6.2 | 93.0 | 123.0 | 112.0 | 6.8 | 96.0 | 128.0 | -3.2 | -5.8 | -0.7 | 2.548E-02 |
| paO_2_ av 1 | 77.8 | 8.6 | 56.5 | 97.2 | 76.4 | 11.6 | 57.9 | 113.1 | 1.3 | -2.5 | 5.2 | 1.183E-01 |
| paO_2_ av 3 | 77.0 | 8.1 | 59.9 | 99.9 | 73.3 | 8.6 | 55.2 | 102.2 | 3.7 | 0.6 | 6.7 | 9.342E-03 |
| paO_2_ av 5 | 76.8 | 7.8 | 64.1 | 101.0 | 74.6 | 9.3 | 51.1 | 99.1 | 2.2 | -1.0 | 5.4 | 2.106E-01 |
| paO_2_ av 10 | 78.0 | 7.5 | 65.4 | 100.9 | 74.2 | 7.9 | 59.3 | 102.1 | 3.8 | 0.8 | 6.8 | 1.310E-02 |
| paO_2_ min 1 | 60.5 | 10.1 | 13.3 | 78.9 | 58.6 | 10.5 | 37.8 | 97.0 | 1.9 | -1.8 | 5.7 | 5.832E-02 |
| paO_2_ min 3 | 65.8 | 7.8 | 41.2 | 89.0 | 63.0 | 5.8 | 50.1 | 81.6 | 2.9 | 0.5 | 5.3 | 1.478E-02 |
| paO_2_ min 5 | 66.2 | 6.9 | 47.7 | 79.6 | 62.1 | 8.6 | 37.3 | 84.8 | 4.0 | 1.1 | 7.0 | 2.141E-03 |
| paO_2_ min 10 | 66.0 | 8.9 | 33.1 | 85.4 | 61.8 | 8.0 | 37.0 | 73.7 | 4.2 | 1.0 | 7.4 | 1.017E-02 |
| paO_2_ max 1 | 101.3 | 21.1 | 66.0 | 167.0 | 100.7 | 26.8 | 64.6 | 182.0 | 0.7 | -8.3 | 9.7 | 3.918E-01 |
| paO_2_ max 3 | 89.9 | 13.7 | 62.5 | 133.0 | 88.7 | 18.0 | 62.2 | 151.0 | 1.2 | -4.8 | 7.2 | 3.271E-01 |
| paO_2_ max 5 | 89.2 | 13.9 | 69.2 | 140.0 | 89.6 | 18.9 | 55.1 | 136.0 | -0.3 | -6.6 | 6.0 | 7.573E-01 |
| paO_2_ max 10 | 92.4 | 15.0 | 70.7 | 137.0 | 91.2 | 16.8 | 65.4 | 130.0 | 1.2 | -5.0 | 7.4 | 5.292E-01 |

|  | Survivors  SD  min  max | | | | | Non-survivors | | | | 95%-CI of MD | | | p |
| --- | --- | --- | --- | --- | --- | --- | --- | --- | --- | --- | --- | --- | --- |
|  | mean | SD | | min | max | mean | SD | min | max | mean | min | max |  |
| paCO_2_ av 1 | 43.1 | 6.4 | 30.9 | | 61.3 | 42.9 | 5.5 | 30.8 | 53.3 | 0.2 | -1.9 | 2.3 | 7.980E-01 |
| paCO_2_ av 3 | 46.0 | 5.7 | 35.1 | | 63.1 | 44.9 | 4.7 | 35.7 | 54.5 | 1.1 | -0.8 | 2.9 | 4.788E-01 |
| paCO_2_ av 5 | 45.5 | 5.2 | 34.9 | | 59.1 | 46.2 | 5.2 | 35.7 | 55.9 | -0.7 | -2.5 | 1.2 | 4.297E-01 |
| paCO_2_ av 10 | 46.2 | 4.7 | 34.9 | | 60.4 | 44.8 | 4.9 | 32.8 | 54.6 | 1.4 | -0.5 | 3.2 | 2.934E-01 |
| paCO_2_ min 1 | 35.5 | 6.9 | 20.5 | | 56.1 | 36.4 | 6.2 | 24.6 | 50.2 | -0.9 | -3.2 | 1.5 | 2.629E-01 |
| paCO_2_ min 3 | 41.9 | 5.5 | 31.8 | | 59.1 | 41.0 | 4.7 | 31.5 | 50.6 | 0.8 | -1.0 | 2.7 | 4.239E-01 |
| paCO_2_ min 5 | 41.5 | 5.6 | 30.6 | | 55.0 | 41.9 | 5.5 | 31.5 | 54.1 | -0.4 | -2.4 | 1.6 | 7.238E-01 |
| paCO_2_ min 10 | 42.0 | 5.3 | 30.2 | | 59.2 | 40.9 | 4.5 | 29.1 | 52.1 | 1.1 | -0.7 | 2.9 | 3.756E-01 |
| paCO_2_ max 1 | 50.8 | 9.8 | 33.5 | | 97.4 | 49.0 | 6.4 | 35.1 | 61.9 | 1.8 | -1.1 | 4.6 | 7.551E-01 |
| paCO_2_ max 3 | 50.2 | 6.8 | 37.0 | | 70.8 | 48.9 | 5.8 | 37.6 | 60.9 | 1.4 | -0.9 | 3.6 | 4.056E-01 |
| paCO_2_ max 5 | 49.6 | 6.1 | 36.4 | | 66.7 | 50.2 | 6.5 | 36.8 | 63.9 | -0.5 | -2.8 | 1.8 | 5.871E-01 |
| paCO_2_ max 10 | 50.7 | 5.5 | 39.1 | | 66.8 | 48.6 | 6.0 | 36.5 | 60.4 | 2.1 | -0.1 | 4.3 | 1.138E-01 |
| Hb av 1 | 10.2 | 1.5 | 7.8 | | 14.4 | 9.8 | 1.6 | 6.5 | 13.8 | 0.4 | -0.1 | 1.0 | 1.466E-01 |
| Hb av 3 | 9.7 | 1.3 | 7.7 | | 14.3 | 9.5 | 1.0 | 8.0 | 11.6 | 0.1 | -0.3 | 0.5 | 5.669E-01 |
| Hb av 5 | 9.9 | 1.3 | 7.8 | | 14.3 | 9.4 | 1.1 | 7.7 | 13.0 | 0.4 | 0.0 | 0.9 | 7.487E-02 |
| Hb av 10 | 9.4 | 1.0 | 8.0 | | 12.6 | 9.0 | 0.6 | 8.0 | 9.8 | 0.4 | 0.1 | 0.7 | 6.652E-02 |
| Hb min 1 | 9.4 | 1.5 | 6.4 | | 13.5 | 8.7 | 1.6 | 5.7 | 12.7 | 0.7 | 0.1 | 1.2 | 3.017E-02 |
| Hb min 3 | 9.0 | 1.2 | 6.7 | | 13.6 | 9.0 | 1.0 | 7.6 | 11.1 | -0.1 | -0.5 | 0.3 | 5.817E-01 |
| Hb min 5 | 9.3 | 1.2 | 7.2 | | 14.0 | 8.8 | 1.1 | 7.1 | 12.4 | 0.5 | 0.0 | 0.9 | 4.610E-02 |
| Hb min 10 | 8.7 | 1.1 | 6.6 | | 12.0 | 8.3 | 0.7 | 5.4 | 9.5 | 0.4 | 0.1 | 0.7 | 1.032E-01 |
| Hb max 1 | 11.3 | 1.8 | 8.3 | | 16.5 | 11.1 | 1.8 | 8.5 | 15.3 | 0.2 | -0.4 | 0.9 | 4.957E-01 |
| Hb max 3 | 10.4 | 1.9 | 8.0 | | 19.2 | 10.1 | 1.1 | 8.3 | 12.6 | 0.3 | -0.2 | 0.9 | 5.886E-01 |
| Hb max 5 | 10.5 | 1.5 | 8.2 | | 14.7 | 10.1 | 1.3 | 8.2 | 13.4 | 0.4 | -0.1 | 0.9 | 1.283E-01 |
| Hb max 10 | 10.1 | 1.1 | 8.5 | | 13.0 | 9.6 | 0.7 | 8.3 | 10.8 | 0.6 | 0.2 | 0.9 | 7.325E-03 |
| Lactate av 1 | 13.9 | 5.0 | 6.8 | | 32.1 | 14.3 | 5.2 | 6.8 | 29.2 | -0.4 | -2.2 | 1.5 | 7.998E-01 |
| Lactate av 3 | 10.5 | 3.2 | 5.0 | | 21.6 | 11.4 | 4.0 | 4.8 | 24.2 | -0.9 | -2.3 | 0.5 | 1.758E-01 |
| Lactate av 5 | 10.5 | 3.5 | 5.4 | | 19.2 | 10.4 | 4.0 | 3.7 | 26.5 | 0.1 | -1.2 | 1.5 | 8.506E-01 |
| Lactate av 10 | 10.7 | 4.2 | 2.7 | | 19.7 | 11.7 | 7.9 | 3.7 | 45.6 | -0.9 | -3.5 | 1.7 | 8.482E-01 |
| Lactate max 1 | 19.8 | 7.7 | 8.0 | | 41.0 | 21.5 | 9.1 | 10.0 | 49.0 | -1.7 | -4.8 | 1.4 | 4.005E-01 |
| Lactate max 3 | 13.7 | 4.7 | 6.0 | | 29.0 | 14.6 | 5.1 | 7.0 | 28.0 | -0.9 | -2.7 | 0.9 | 2.792E-01 |
| Lactate max 5 | 14.0 | 5.0 | 7.0 | | 28.0 | 13.4 | 5.4 | 5.0 | 32.0 | 0.6 | -1.3 | 2.5 | 5.590E-01 |
| Lactate max 10 | 14.6 | 6.3 | 3.0 | | 36.0 | 16.0 | 12.5 | 5.0 | 71.0 | -1.4 | -5.5 | 2.7 | 7.174E-01 |
| Trop max 1 | 48.4 | 81.0 | 5.6 | | 614.0 | 69.1 | 67.7 | 9.4 | 274.0 | -20.7 | -48.1 | 6.6 | 3.936E-03 |
| Trop max 3 | 20.6 | 19.3 | 4.8 | | 102.0 | 35.7 | 37.5 | 8.9 | 174.0 | -15.1 | -32.9 | 2.6 | 2.093E-02 |
| Trop max 5 | 28.3 | 60.6 | 4.3 | | 466.0 | 51.2 | 70.0 | 4.5 | 424.0 | -22.8 | -50.4 | 4.7 | 2.664E-04 |
| Trop max 10 | 28.2 | 24.7 | 5.7 | | 115.0 | 76.3 | 153.1 | 4.5 | 940.0 | -48.2 | -99.6 | 3.3 | 3.532E-03 |
| GFR min 1 | 84.5 | 30.6 | 12.0 | | 140.0 | 76.3 | 34.8 | 14.0 | 126.0 | 8.2 | -3.9 | 20.3 | 2.639E-01 |
| GFR min 3 | 89.8 | 29.9 | 16.0 | | 149.0 | 83.6 | 30.8 | 18.0 | 127.0 | 6.3 | -4.9 | 17.4 | 3.125E-01 |
| GFR min 5 | 90.3 | 29.8 | 17.0 | | 149.0 | 86.3 | 29.8 | 21.0 | 130.0 | 4.1 | -6.8 | 14.9 | 5.067E-01 |
| GFR min 10 | 98.7 | 27.1 | 26.0 | | 153.0 | 91.4 | 29.4 | 31.0 | 140.0 | 7.3 | -3.7 | 18.3 | 2.547E-01 |
| Crea max 1 | 1.1 | 0.7 | 0.3 | | 4.0 | 1.3 | 0.9 | 0.3 | 4.7 | -0.2 | -0.5 | 0.1 | 5.366E-01 |
| Crea max 3 | 1.0 | 0.6 | 0.4 | | 3.6 | 1.1 | 0.6 | 0.4 | 2.8 | -0.1 | -0.3 | 0.2 | 6.285E-01 |
| Crea max 5 | 1.0 | 0.6 | 0.4 | | 3.0 | 1.0 | 0.6 | 0.4 | 3.2 | 0.0 | -0.3 | 0.2 | 8.122E-01 |
| Crea max 10 | 0.8 | 0.4 | 0.3 | | 3.0 | 0.9 | 0.5 | 0.3 | 2.7 | -0.1 | -0.3 | 0.1 | 7.853E-01 |
| Urea max 1 | 62.0 | 36.8 | 20.0 | | 263.0 | 77.1 | 36.3 | 12.0 | 162.0 | -15.0 | -28.2 | -1.8 | 7.666E-03 |
| Urea max 3 | 70.7 | 36.2 | 27.0 | | 209.0 | 78.6 | 42.2 | 14.0 | 184.0 | -7.9 | -22.6 | 6.7 | 4.249E-01 |
| Urea max 5 | 77.7 | 37.4 | 20.0 | | 204.0 | 84.0 | 40.7 | 23.0 | 209.0 | -6.3 | -20.6 | 8.1 | 3.650E-01 |
| Urea max 10 | 79.3 | 47.1 | 26.0 | | 238.0 | 88.3 | 45.5 | 37.0 | 262.0 | -9.1 | -26.8 | 8.6 | 4.599E-02 |
| AST max 1 | 92.3 | 132.4 | 22.0 | | 917.0 | 83.0 | 111.8 | 24.0 | 802.0 | 9.3 | -34.2 | 52.9 | 9.457E-01 |
| AST max 3 | 70.9 | 49.9 | 20.0 | | 336.0 | 71.8 | 38.4 | 21.0 | 192.0 | -0.9 | -16.8 | 14.9 | 4.771E-01 |
| AST max 5 | 70.4 | 57.5 | 23.0 | | 402.0 | 88.1 | 65.6 | 24.0 | 291.0 | -17.7 | -40.7 | 5.2 | 1.528E-01 |
| AST max 10 | 63.8 | 59.0 | 13.0 | | 314.0 | 398.4 | 1939 | 17.0 | 12809.0 | -331.7 | -928.6 | 265.3 | 1.829E-03 |
| ALT max 1 | 74.1 | 112.2 | 12.0 | | 978.0 | 72.2 | 93.0 | 10.0 | 622.0 | 1.9 | -34.7 | 38.5 | 7.398E-01 |
| ALT max 3 | 68.7 | 72.7 | 15.0 | | 586.0 | 77.0 | 87.9 | 11.0 | 555.0 | -8.3 | -38.5 | 21.9 | 9.375E-01 |
| ALT max 5 | 87.5 | 67.2 | 19.0 | | 302.0 | 95.3 | 73.7 | 9.0 | 266.0 | -7.8 | -34.0 | 18.4 | 7.237E-01 |
| ALT max 10 | 87.4 | 66.7 | 16.0 | | 360.0 | 271.3 | 898.3 | 19.0 | 5952 | -183.9 | -460.8 | 92.9 | 4.571E-02 |
| LDH max 1 | 508 | 248 | 202 | | 1255 | 462 | 197 | 223 | 1166 | 47 | -32 | 125 | 4.670E-01 |
| LDH max 3 | 426 | 157 | 220 | | 997 | 417 | 145 | 188 | 928 | 9 | -46 | 64 | 9.266E-01 |
| LDH max 5 | 419 | 136 | 191 | | 747 | 442 | 185 | 211 | 1077 | -23 | -85 | 39 | 7.144E-01 |
| LDH max 10 | 411 | 152 | 158 | | 957 | 782 | 2229 | 236 | 14973 | -372 | -1058 | 315 | 7.041E-01 |
| CRP max 1 | 195.9 | 125.3 | 6.3 | | 547.0 | 209.6 | 102.4 | 0.6 | 473.0 | -13.7 | -53.9 | 26.6 | 3.103E-01 |
| CRP max 3 | 109.1 | 89.8 | 1.1 | | 388.0 | 137.8 | 85.4 | 0.6 | 459.0 | -28.7 | -60.5 | 3.0 | 2.338E-02 |
| CRP max 5 | 73.6 | 79.1 | 0.6 | | 339.0 | 114.6 | 95.5 | 0.6 | 319.0 | -41.0 | -74.1 | -8.0 | 4.630E-03 |
| CRP max 10 | 68.7 | 86.2 | 0.6 | | 443.0 | 100.8 | 111.9 | 2.9 | 536.0 | -32.2 | -71.8 | 7.5 | 6.016E-02 |
| PCT max 1 | 1.3 | 2.3 | 0.1 | | 13.1 | 2.9 | 5.8 | 0.1 | 34.2 | -1.6 | -3.4 | 0.1 | 1.491E-02 |
| PCT max 3 | 0.7 | 1.4 | 0.1 | | 8.7 | 1.3 | 2.1 | 0.1 | 12.6 | -0.5 | -1.2 | 0.2 | 1.092E-03 |
| PCT max 5 | 0.4 | 0.5 | 0.1 | | 2.9 | 0.7 | 0.7 | 0.1 | 2.5 | -0.4 | -0.6 | -0.1 | 1.281E-03 |
| PCT max 10 | 0.3 | 0.5 | 0.1 | | 3.4 | 1.1 | 2.6 | 0.1 | 14.2 | -0.8 | -1.6 | 0.0 | 3.448E-03 |

|  | Survivors  SD  min  max | | | | Non-survivors | | | | 95%-CI of MD | | | p |
| --- | --- | --- | --- | --- | --- | --- | --- | --- | --- | --- | --- | --- |
|  | mean | SD | min | max | mean | SD | min | max | mean | min | max |  |
| WBC max 1 | 15.0 | 8.3 | 2.9 | 47.1 | 14.8 | 7.9 | 4.3 | 50.0 | 0.2 | -2.7 | 3.1 | 9.198E-01 |
| WBC max 3 | 11.2 | 4.6 | 4.2 | 27.1 | 11.2 | 5.5 | 4.4 | 28.6 | 0.0 | -1.9 | 1.9 | 7.455E-01 |
| WBC max 5 | 13.5 | 6.9 | 3.5 | 56.3 | 12.4 | 5.9 | 5.2 | 29.6 | 1.1 | -1.2 | 3.4 | 2.017E-01 |
| WBC max 10 | 11.5 | 4.8 | 3.8 | 29.9 | 11.1 | 5.7 | 4.0 | 30.0 | 0.4 | -1.7 | 2.5 | 2.672E-01 |
| Ferritin max 1 | 1933 | 1711 | 198 | 10210 | 1857 | 1228 | 442 | 4886 | 76 | -506 | 659 | 5.778E-01 |
| Ferritin max 3 | 1520 | 1094 | 240 | 5396 | 1582 | 1054 | 297 | 4766 | -62 | -555 | 430 | 5.871E-01 |
| Ferritin max 5 | 1379 | 1106 | 67 | 5318 | 1953 | 1534 | 385 | 6687 | -574 | -1279 | 131 | 7.538E-02 |
| Ferritin max 10 | 1528 | 2921 | 192 | 19268 | 2153 | 2664 | 295 | 13382 | -625 | -1969 | 719 | 8.752E-02 |
| Lymphos max 1 | 1.1 | 0.6 | 0.2 | 4.4 | 1.1 | 0.7 | 0.3 | 3.1 | 0.0 | -0.3 | 0.2 | 6.439E-01 |
| Lymphos max 3 | 1.2 | 0.5 | 0.3 | 2.7 | 1.1 | 0.6 | 0.3 | 2.7 | 0.1 | -0.1 | 0.3 | 2.026E-01 |
| Lymphos max 5 | 1.5 | 0.7 | 0.3 | 3.0 | 1.3 | 0.8 | 0.3 | 4.3 | 0.1 | -0.1 | 0.4 | 1.128E-01 |
| Lymphos max 10 | 1.3 | 0.6 | 0.3 | 2.8 | 1.4 | 0.8 | 0.3 | 3.7 | 0.0 | -0.3 | 0.2 | 6.119E-01 |
| IL6 max 1 | 514 | 983 | 5 | 5545 | 680 | 1447 | 22 | 8140 | -166.6 | -636.3 | 303.1 | 1.978E-01 |
| IL6 max 3 | 133 | 271 | 3 | 1391 | 205 | 333 | 3 | 1475 | -72.4 | -220.4 | 75.5 | 3.263E-02 |
| IL6 max 5 | 114 | 222 | 3 | 1094 | 237 | 468 | 3 | 2358 | -122.6 | -273.4 | 28.2 | 4.660E-02 |
| IL6 max 10 | 116 | 368 | 3 | 2625 | 120 | 213 | 3 | 970 | -4.0 | -115.0 | 107.0 | 5.493E-02 |
| INR av 1 | 1.1 | 0.2 | 0.9 | 2.1 | 1.2 | 0.2 | 1.0 | 1.5 | -0.1 | -0.1 | 0.0 | 1.822E-02 |
| INR av 3 | 1.2 | 0.2 | 0.9 | 2.0 | 1.2 | 0.2 | 0.9 | 1.9 | 0.0 | -0.1 | 0.0 | 1.641E-01 |
| INR av 5 | 1.2 | 0.2 | 0.9 | 1.9 | 1.2 | 0.2 | 0.9 | 2.0 | 0.0 | -0.1 | 0.0 | 1.938E-01 |
| INR av 10 | 1.3 | 0.3 | 0.9 | 2.3 | 1.3 | 0.3 | 1.0 | 2.4 | 0.0 | -0.2 | 0.1 | 4.945E-01 |
| D-Dim. max 1 | 9.5 | 10.7 | 0.5 | 35.0 | 10.6 | 11.2 | 0.8 | 35.0 | -1.1 | -5.1 | 3.0 | 6.070E-01 |
| D-Dim. max 3 | 11.6 | 10.9 | 0.5 | 35.0 | 9.6 | 8.8 | 0.5 | 35.0 | 2.0 | -1.5 | 5.5 | 5.867E-01 |
| D-Dim. max 5 | 14.0 | 11.5 | 0.7 | 35.0 | 14.7 | 11.9 | 0.8 | 35.0 | -0.6 | -4.9 | 3.7 | 8.065E-01 |
| D-Dim. max 10 | 23.9 | 11.6 | 2.4 | 35.0 | 21.3 | 11.0 | 2.5 | 35.0 | 2.6 | -1.8 | 6.9 | 2.488E-01 |
| Platelets av 1 | 299.6 | 117.7 | 99.0 | 702.0 | 240.0 | 121.1 | 53.0 | 575.0 | 59.6 | 16.3 | 103.0 | 2.685E-03 |
| Platelets av 3 | 260.6 | 90.7 | 72.0 | 523.0 | 204.8 | 97.4 | 56.0 | 438.0 | 55.8 | 21.2 | 90.5 | 1.992E-03 |
| Platelets av 5 | 251.0 | 97.7 | 72.0 | 506.0 | 189.9 | 82.4 | 59.0 | 381.0 | 61.1 | 29.0 | 93.2 | 1.057E-03 |
| Platelets av 10 | 199.2 | 88.0 | 53.0 | 463.0 | 147.1 | 57.9 | 47.0 | 242.0 | 52.1 | 25.0 | 79.2 | 1.636E-03 |
| Norepi av 1 | 0.3 | 0.3 | 0.0 | 1.4 | 0.3 | 0.3 | 0.0 | 0.9 | 0.0 | -0.1 | 0.1 | 6.661E-01 |
| Norepi av 3 | 0.1 | 0.2 | 0.0 | 0.7 | 0.1 | 0.1 | 0.0 | 0.6 | 0.0 | -0.1 | 0.0 | 1.140E-01 |
| Norepi av 5 | 0.1 | 0.2 | 0.0 | 1.3 | 0.2 | 0.3 | 0.0 | 1.4 | -0.1 | -0.1 | 0.0 | 2.292E-01 |
| Norepi av 10 | 0.1 | 0.2 | 0.0 | 1.1 | 0.3 | 0.5 | 0.0 | 2.9 | -0.1 | -0.3 | 0.0 | 2.869E-01 |
| Norepi max 1 | 0.7 | 1.0 | 0.0 | 7.6 | 0.6 | 0.5 | 0.0 | 2.8 | 0.1 | -0.2 | 0.4 | 7.840E-01 |
| Norepi max 3 | 0.2 | 0.2 | 0.0 | 1.0 | 0.3 | 0.3 | 0.0 | 1.3 | -0.1 | -0.2 | 0.0 | 2.487E-02 |
| Norepi max 5 | 0.2 | 0.3 | 0.0 | 1.5 | 0.3 | 0.4 | 0.0 | 1.7 | -0.1 | -0.2 | 0.0 | 1.465E-01 |
| Norepi max 10 | 0.2 | 0.3 | 0.0 | 1.5 | 0.5 | 0.8 | 0.0 | 4.0 | -0.2 | -0.5 | 0.0 | 2.633E-01 |
| MAP av 1 | 77.9 | 7.1 | 65.1 | 109.2 | 77.9 | 6.1 | 68.1 | 98.3 | 0.0 | -2.4 | 2.3 | 9.894E-01 |
| MAP av 3 | 81.0 | 8.8 | 68.7 | 114.2 | 78.4 | 8.0 | 64.9 | 107.4 | 2.6 | -0.4 | 5.6 | 6.490E-02 |
| MAP av 5 | 78.9 | 7.7 | 66.6 | 103.6 | 76.1 | 6.0 | 67.5 | 92.1 | 2.8 | 0.4 | 5.2 | 4.622E-02 |
| MAP av 10 | 78.3 | 7.6 | 65.6 | 95.0 | 75.8 | 7.4 | 57.5 | 93.2 | 2.4 | -0.4 | 5.3 | 1.811E-01 |
| MAP min 1 | 61.4 | 7.8 | 36.0 | 89.0 | 61.8 | 6.2 | 50.0 | 74.0 | -0.4 | -2.9 | 2.0 | 8.127E-01 |
| MAP min 3 | 65.4 | 7.3 | 50.0 | 91.0 | 62.1 | 8.9 | 49.0 | 97.0 | 3.3 | 0.3 | 6.3 | 7.448E-03 |
| MAP min 5 | 62.8 | 7.1 | 45.0 | 83.0 | 61.6 | 6.7 | 44.0 | 77.0 | 1.2 | -1.3 | 3.7 | 5.763E-01 |
| MAP min 10 | 63.6 | 7.8 | 34.0 | 80.0 | 60.2 | 7.4 | 45.0 | 80.0 | 3.4 | 0.5 | 6.3 | 1.156E-02 |
| MAP max 1 | 103.5 | 14.9 | 76.0 | 144.0 | 105.2 | 15.2 | 81.0 | 139.0 | -1.7 | -7.1 | 3.7 | 6.647E-01 |
| MAP max 3 | 100.8 | 14.4 | 57.0 | 144.0 | 98.1 | 13.6 | 75.0 | 138.0 | 2.7 | -2.3 | 7.8 | 2.416E-01 |
| MAP max 5 | 99.5 | 13.2 | 75.0 | 134.0 | 94.5 | 10.9 | 78.0 | 130.0 | 5.0 | 0.7 | 9.3 | 3.197E-02 |
| MAP max 10 | 99.6 | 14.5 | 79.0 | 161.0 | 95.5 | 14.5 | 70.0 | 143.0 | 4.1 | -1.4 | 9.3 | 1.184E-01 |
| HR av 1 | 76.4 | 13.6 | 48.5 | 110.2 | 81.4 | 16.6 | 55.2 | 127.3 | -5.1 | -10.7 | 0.6 | 1.108E-01 |
| HR av 3 | 72.9 | 14.5 | 43.4 | 113.9 | 76.0 | 15.8 | 48.0 | 114.5 | -3.1 | -8.7 | 2.5 | 2.763E-01 |
| HR av 5 | 72.8 | 14.3 | 41.9 | 111.5 | 75.8 | 16.9 | 47.4 | 117.0 | -2.9 | -8.8 | 2.9 | 4.168E-01 |
| HR av 10 | 78.1 | 13.6 | 42.6 | 108.1 | 77.7 | 15.1 | 49.6 | 107.3 | 0.4 | -5.2 | 6.0 | 7.614E-01 |
| HR min 1 | 65.5 | 12.3 | 43.0 | 98.0 | 69.0 | 13.8 | 45.0 | 99.0 | -3.5 | -8.3 | 1.3 | 1.657E-01 |
| HR min 3 | 63.8 | 13.4 | 39.0 | 104.0 | 68.6 | 14.4 | 43.0 | 97.0 | -4.8 | -9.9 | 0.3 | 6.624E-02 |
| HR min 5 | 64.4 | 14.1 | 36.0 | 109.0 | 67.4 | 15.2 | 40.0 | 96.0 | -3.0 | -8.4 | 2.4 | 3.209E-01 |
| HR min 10 | 68.8 | 13.8 | 39.0 | 102.0 | 69.6 | 14.2 | 44.0 | 100.0 | -0.8 | -6.2 | 4.6 | 8.594E-01 |
| HR max 1 | 95.2 | 20.5 | 52.0 | 169.0 | 100.8 | 20.2 | 65.0 | 155.0 | -5.6 | -13.0 | 1.7 | 1.247E-01 |
| HR max 3 | 87.6 | 19.2 | 48.0 | 139.0 | 88.7 | 19.5 | 55.0 | 129.0 | -1.1 | -8.1 | 6.0 | 6.655E-01 |
| HR max 5 | 88.4 | 18.8 | 46.0 | 145.0 | 89.0 | 21.6 | 54.0 | 162.0 | -0.6 | -8.1 | 6.9 | 9.578E-01 |
| HR max 10 | 95.8 | 18.4 | 51.0 | 138.0 | 92.2 | 22.6 | 59.0 | 197.0 | 3.6 | -4.5 | 11.7 | 1.412E-01 |

|  | Survivors  SD  min  max | | | | Non-survivors | | | | 95%-CI of MD  min  max | | | p |
| --- | --- | --- | --- | --- | --- | --- | --- | --- | --- | --- | --- | --- |
|  | mean | SD | min | max | mean | SD | min | max | mean | min | max |  |
| flow av 1 | 3.3 | 0.5 | 2.2 | 4.3 | 3.3 | 0.5 | 2.0 | 4.5 | 0.0 | -0.2 | 0.1 | 6.525E-01 |
| flow av 3 | 3.3 | 0.6 | 2.2 | 4.8 | 3.4 | 0.6 | 1.9 | 4.8 | -0.1 | -0.4 | 0.1 | 2.070E-01 |
| flow av 5 | 3.0 | 0.7 | 1.4 | 4.7 | 3.4 | 0.7 | 1.3 | 4.8 | -0.3 | -0.6 | -0.1 | 7.080E-03 |
| flow av 10 | 2.8 | 0.8 | 1.1 | 4.5 | 3.4 | 0.8 | 2.0 | 5.5 | -0.7 | -1.0 | -0.3 | 2.504E-04 |
| flow min 1 | 3.0 | 0.5 | 1.8 | 4.0 | 3.0 | 0.6 | 1.3 | 4.4 | 0.0 | -0.2 | 0.2 | 7.403E-01 |
| flow min 3 | 3.0 | 0.7 | 1.6 | 4.4 | 3.1 | 0.7 | 1.2 | 4.5 | -0.1 | -0.4 | 0.1 | 2.997E-01 |
| flow min 5 | 2.7 | 0.8 | 0.8 | 4.6 | 3.1 | 0.7 | 1.2 | 4.6 | -0.4 | -0.6 | -0.1 | 4.645E-03 |
| flow min 10 | 2.5 | 0.9 | 0.3 | 3.9 | 3.2 | 0.9 | 1.3 | 5.3 | -0.7 | -1.1 | -0.4 | 1.001E-04 |
| flow max 1 | 3.5 | 0.5 | 2.4 | 4.5 | 3.6 | 0.6 | 2.1 | 5.0 | -0.1 | -0.3 | 0.1 | 2.787E-01 |
| flow max 3 | 3.4 | 0.7 | 2.4 | 5.2 | 3.6 | 0.6 | 1.9 | 5.1 | -0.1 | -0.4 | 0.1 | 1.938E-01 |
| flow max 5 | 3.2 | 0.7 | 1.6 | 5.0 | 3.5 | 0.7 | 1.4 | 5.1 | -0.3 | -0.6 | 0.0 | 1.291E-02 |
| flow max 10 | 3.0 | 0.8 | 1.2 | 4.7 | 3.6 | 0.8 | 2.0 | 5.7 | -0.6 | -0.9 | -0.3 | 6.533E-04 |
| sweep av 1 | 4.4 | 1.4 | 2.1 | 10.0 | 4.2 | 1.3 | 1.8 | 7.8 | 0.2 | -0.3 | 0.6 | 5.906E-01 |
| sweep av 3 | 5.0 | 1.6 | 2.0 | 11.3 | 5.4 | 1.7 | 1.8 | 8.5 | -0.4 | -1.0 | 0.2 | 1.282E-01 |
| sweep av 5 | 5.1 | 2.1 | 2.0 | 12.0 | 5.8 | 2.1 | 0.9 | 12.0 | -0.7 | -1.4 | 0.1 | 2.817E-02 |
| sweep av 10 | 5.0 | 2.3 | 0.8 | 12.0 | 6.7 | 2.5 | 2.0 | 11.0 | -1.7 | -2.6 | -0.8 | 4.299E-04 |
| sweep min 1 | 3.8 | 1.4 | 2.0 | 10.0 | 3.6 | 1.3 | 1.0 | 7.0 | 0.1 | -0.4 | 0.6 | 6.650E-01 |
| sweep min 3 | 4.7 | 1.7 | 1.5 | 11.0 | 5.2 | 1.7 | 1.5 | 8.0 | -0.5 | -1.1 | 0.1 | 4.664E-02 |
| sweep min 5 | 4.9 | 2.2 | 0.0 | 12.0 | 5.6 | 2.1 | 0.0 | 12.0 | -0.7 | -1.5 | 0.1 | 3.376E-02 |
| sweep min 10 | 4.7 | 2.5 | 0.0 | 12.0 | 6.3 | 2.4 | 2.0 | 10.0 | -1.6 | -2.6 | -0.7 | 1.048E-03 |
| sweep max 1 | 5.3 | 1.5 | 3.0 | 10.0 | 5.2 | 1.6 | 2.0 | 10.0 | 0.1 | -0.5 | 0.7 | 6.552E-01 |
| sweep max 3 | 5.4 | 1.8 | 2.0 | 12.0 | 5.7 | 1.8 | 2.0 | 9.0 | -0.3 | -1.0 | 0.3 | 2.193E-01 |
| sweep max 5 | 5.5 | 2.1 | 2.0 | 12.0 | 6.1 | 2.1 | 1.0 | 12.0 | -0.6 | -1.4 | 0.1 | 4.435E-02 |
| sweep max 10 | 5.4 | 2.3 | 1.0 | 12.0 | 7.1 | 2.7 | 2.0 | 12.0 | -1.7 | -2.7 | -0.7 | 8.353E-04 |
| FiO_2_ av 1 | 60.4 | 14.0 | 36.2 | 96.9 | 64.5 | 15.9 | 32.6 | 98.3 | -4.1 | -9.6 | 1.4 | 8.680E-02 |
| FiO_2_ av 3 | 56.1 | 15.3 | 33.7 | 98.8 | 61.3 | 18.5 | 35.1 | 100.0 | -5.2 | -11.6 | 1.2 | 1.681E-01 |
| FiO_2_ av 5 | 53.5 | 15.8 | 32.1 | 97.4 | 61.6 | 17.9 | 37.1 | 100.0 | -8.1 | -14.4 | -1.9 | 8.359E-03 |
| FiO_2_ av 10 | 50.5 | 15.5 | 30.7 | 100.0 | 60.7 | 18.0 | 35.0 | 100.0 | -10.2 | -16.8 | -3.6 | 9.882E-04 |
| FiO_2_ min 1 | 51.5 | 13.6 | 30.0 | 98.0 | 54.0 | 14.4 | 29.9 | 85.0 | -2.5 | -7.6 | 2.6 | 3.138E-01 |
| FiO_2_ min 3 | 49.8 | 13.3 | 29.0 | 90.0 | 54.6 | 17.2 | 30.0 | 100.0 | -4.7 | -10.5 | 1.1 | 1.306E-01 |
| FiO_2_ min 5 | 47.7 | 13.2 | 30.0 | 90.0 | 55.8 | 17.4 | 29.4 | 100.0 | -8.1 | -14.0 | -2.3 | 8.308E-03 |
| FiO_2_ min 10 | 45.3 | 14.1 | 30.0 | 100.0 | 55.6 | 18.3 | 35.0 | 100.0 | -10.4 | -16.9 | -3.8 | 2.240E-04 |
| FiO_2_ max 1 | 81.8 | 19.6 | 40.0 | 100.0 | 86.8 | 17.6 | 41.0 | 100.0 | -5.0 | -11.6 | 1.6 | 1.283E-01 |
| FiO_2_ max 3 | 69.6 | 22.0 | 35.0 | 100.0 | 70.3 | 22.9 | 36.0 | 100.0 | -0.7 | -8.9 | 7.5 | 7.142E-01 |
| FiO_2_ max 5 | 67.1 | 22.9 | 35.0 | 100.0 | 77.2 | 21.7 | 40.0 | 100.0 | -10.1 | -18.2 | -2.1 | 1.190E-02 |
| FiO_2_ max 10 | 65.7 | 23.1 | 35.0 | 100.0 | 74.6 | 23.7 | 36.0 | 100.0 | -9.0 | -18.0 | 0.1 | 3.541E-02 |
| HV av 1 | 138.7 | 40.4 | 62.3 | 243.8 | 129.7 | 49.3 | 67.8 | 301.5 | 9.0 | -7.7 | 25.7 | 7.075E-02 |
| HV av 3 | 149.6 | 44.7 | 62.8 | 262.4 | 132.1 | 42.6 | 60.7 | 229.8 | 17.5 | 1.7 | 33.3 | 3.755E-02 |
| HV av 5 | 156.4 | 47.9 | 71.0 | 280.8 | 135.7 | 49.4 | 51.0 | 277.7 | 20.7 | 2.9 | 38.5 | 1.640E-02 |
| HV av 10 | 168.5 | 47.5 | 66.0 | 307.2 | 135.5 | 41.8 | 59.2 | 220.5 | 33.0 | 16.1 | 50.0 | 2.570E-04 |
| HV min 1 | 102.6 | 35.1 | 28.0 | 193.0 | 99.0 | 56.7 | 42.0 | 332.0 | 3.6 | -14.5 | 21.7 | 4.147E-02 |
| HV min 3 | 124.5 | 39.1 | 42.0 | 236.0 | 110.8 | 37.2 | 53.0 | 193.0 | 13.7 | -0.1 | 27.5 | 6.062E-02 |
| HV min 5 | 129.5 | 42.2 | 53.0 | 237.0 | 109.9 | 39.6 | 47.0 | 210.0 | 19.5 | 4.8 | 34.3 | 8.542E-03 |
| HV min 10 | 135.3 | 47.7 | 58.0 | 281.0 | 111.5 | 36.9 | 46.0 | 198.0 | 23.8 | 7.9 | 39.7 | 9.383E-03 |
| HV max 1 | 176.7 | 54.6 | 82.0 | 337.0 | 162.6 | 56.7 | 89.0 | 328.0 | 14.2 | -6.0 | 34.4 | 1.291E-01 |
| HV max 3 | 177.0 | 53.9 | 76.0 | 327.0 | 160.1 | 55.8 | 64.0 | 316.0 | 16.8 | -3.2 | 36.9 | 8.117E-02 |
| HV max 5 | 182.7 | 58.1 | 81.0 | 346.0 | 161.7 | 62.5 | 55.0 | 338.0 | 21.0 | -1.2 | 43.2 | 3.476E-02 |
| HV max 10 | 198.8 | 57.6 | 75.0 | 377.0 | 166.5 | 56.0 | 69.0 | 288.0 | 32.2 | 10.4 | 54.0 | 3.350E-03 |
| PEEP av 1 | 14.4 | 2.3 | 5.6 | 19.7 | 14.1 | 2.6 | 5.1 | 19.6 | 0.4 | -0.6 | 1.3 | 6.334E-01 |
| PEEP av 3 | 13.9 | 2.8 | 6.8 | 19.6 | 13.9 | 2.8 | 6.4 | 23.2 | 0.0 | -1.0 | 1.0 | 6.592E-01 |
| PEEP av 5 | 13.3 | 2.8 | 6.3 | 18.5 | 13.7 | 2.6 | 5.4 | 21.1 | -0.4 | -1.4 | 0.6 | 8.752E-01 |
| PEEP av 10 | 11.6 | 3.1 | 4.8 | 18.4 | 12.5 | 2.8 | 4.8 | 18.9 | -0.9 | -2.0 | 0.3 | 1.202E-01 |
| RMV av 1 | 3.1 | 1.7 | 0.6 | 13.7 | 3.1 | 1.7 | 1.0 | 10.7 | -0.1 | -0.7 | 0.6 | 8.986E-01 |
| RMV av 3 | 3.3 | 2.0 | 0.9 | 15.4 | 3.0 | 1.7 | 0.6 | 9.0 | 0.3 | -0.3 | 1.0 | 3.932E-01 |
| RMV av 5 | 3.6 | 1.8 | 0.8 | 8.7 | 2.9 | 1.7 | 0.5 | 8.6 | 0.6 | 0.0 | 1.3 | 4.732E-02 |
| RMV av 10 | 4.2 | 2.0 | 0.9 | 12.0 | 3.1 | 2.3 | 0.6 | 13.5 | 1.1 | 0.2 | 1.9 | 5.851E-04 |
| VT av 1 | 259.4 | 94.4 | 59.5 | 521.8 | 249.6 | 90.5 | 80.2 | 459.4 | 9.8 | -23.4 | 43.0 | 5.294E-01 |
| VT av 3 | 266.5 | 109.7 | 74.3 | 580.2 | 229.5 | 104.8 | 61.0 | 549.0 | 37.0 | -1.8 | 75.8 | 7.926E-02 |
| VT av 5 | 274.2 | 116.0 | 72.7 | 642.8 | 216.5 | 102.8 | 47.0 | 497.4 | 57.7 | 18.5 | 96.9 | 3.195E-03 |
| VT av 10 | 292.4 | 121.1 | 85.3 | 818.2 | 209.6 | 104.5 | 54.5 | 536.3 | 82.9 | 40.1 | 125.6 | 2.800E-05 |
| Ppeak av 1 | 24.6 | 3.0 | 15.8 | 31.3 | 25.0 | 3.6 | 13.5 | 34.5 | -0.4 | -1.6 | 0.9 | 3.353E-01 |
| Ppeak av 3 | 24.5 | 3.5 | 11.4 | 35.8 | 25.2 | 3.1 | 17.1 | 31.9 | -0.7 | -1.9 | 0.5 | 2.459E-01 |
| Ppeak av 5 | 24.7 | 3.6 | 15.5 | 33.7 | 25.4 | 3.1 | 14.9 | 31.2 | -0.7 | -2.0 | 0.5 | 2.273E-01 |
| Ppeak av 10 | 24.0 | 3.5 | 15.2 | 31.5 | 25.1 | 3.1 | 16.0 | 31.6 | -1.1 | -2.4 | 0.2 | 7.436E-02 |

|  | Survivors  SD  min  max | | | | Non-survivors | | | | 95%-CI of MD  min  max | | | p |
| --- | --- | --- | --- | --- | --- | --- | --- | --- | --- | --- | --- | --- |
|  | mean | SD | min | max | mean | SD | min | max | mean | min | max |  |
| TISS 1 | 17.6 | 5.0 | 10.0 | 32.0 | 19.0 | 4.2 | 10.0 | 26.0 | -1.5 | -4.2 | 1.3 | 1.426E-01 |
| TISS 3 | 12.4 | 2.9 | 10.0 | 20.0 | 13.0 | 3.5 | 10.0 | 26.0 | -0.5 | -1.7 | 0.7 | 4.342E-01 |
| TISS 5 | 12.3 | 2.8 | 10.0 | 19.0 | 12.7 | 3.0 | 10.0 | 19.0 | -0.5 | -1.6 | 0.6 | 3.596E-01 |
| TISS 10 | 13.4 | 3.6 | 10.0 | 24.0 | 14.2 | 4.8 | 10.0 | 28.0 | -0.8 | -2.5 | 0.9 | 7.125E-01 |
| SAPS 1 | 27.4 | 6.8 | 15.0 | 40.0 | 29.4 | 7.9 | 16.0 | 52.0 | -2.0 | -6.6 | 2.5 | 5.863E-01 |
| SAPS 3 | 21.8 | 5.6 | 8.0 | 35.0 | 24.6 | 6.8 | 14.0 | 38.0 | -2.8 | -5.2 | -0.5 | 4.085E-02 |
| SAPS 5 | 22.1 | 6.0 | 9.0 | 40.0 | 25.7 | 7.4 | 16.0 | 42.0 | -3.6 | -6.1 | -1.0 | 1.905E-02 |
| SAPS 10 | 22.6 | 6.5 | 11.0 | 47.0 | 28.3 | 9.1 | 16.0 | 55.0 | -5.6 | -8.8 | -2.5 | 3.781E-04 |

**Course of ICU treatment.** Significant differences between survivors and non-survivors are highlighted in orange color, highly significant differences in purple color. The values of the individual parameters are given for day 1, day 3, day 5 and day 10 after the start of ECMO therapy. Av, average value for the day; min, minimum value for the day; max, maximum value for the day; HCO_3_^-^, standard bicarbonate; BE base excess, Cl^-^, chloride; paO_2_, partial pressure of oxygen; paCO_2_, partial pressure of carbon dioxide; Hb, hemoglobin; GFR, glomerular filtration rate; AST, aspartate transaminase; ALT, alanine transaminase; LDH, lactate dehydrogenase; CRP, C-reactive protein; PCT, procalcitonin, WBC, white blood cells; IL6, interleukin 6; INR, International Normalized Ratio; D-Dim, d-dimers; MAP, mean arterial pressure; HR, heart rate; flow, pump flow rate; sweep, sweep gas flow rate; FiO_2_, fraction of inspired oxygen; HV, oxygenation ratio; PEEP, positive endexpiratory pressure; RMV, respiratory minute volume; TV, tidal volume; Ppeak, peak inspiratory pressure; TISS, Therapeutic Intervention Scoring System; SAPS, Simplified Acute Physiology Score; CI, confidence interval; MD, mean difference.
