# Supplementary material for: Prognostic factors for favorable outcomes after veno-venous extracorporeal membrane oxygenation in critical care patients with COVID-19
Source: PLoS One. 2023 Jan 20;18(1):e0280502. doi: 10.1371/journal.pone.0280502 (PMC9858373; doi:10.1371/journal.pone.0280502)
Supplement: S3 Table — (DOCX) [file pone.0280502.s005.docx]

**Correlation coefficients (Pearson)**

**Model initial**

|  | **Age** | **Platelets initial** |
| --- | --- | --- |
| **Age** | 1 | -0.116 |
| **Platelets initial** | -0.116 | 1 |

**Model day 10**

|  | **Age** | **pH av** | **paO_2_ min** | **Platelets av** | **ECMO flow av** |
| --- | --- | --- | --- | --- | --- |
| **Age** | 1 | 0.085 | -0.090 | -0.125 | 0.009 |
| **pH av** | 0.085 | 1 | -0.051 | -0.084 | -0.205 |
| **paO_2_ min** | -0.090 | -0.051 | 1 | -0.006 | -0.339 |
| **Platelets av** | -0.125 | -0.084 | -0.006 | 1 | 0.115 |
| **ECMO flow av** | 0.009 | -0.205 | -0.339 | 0.115 | 1 |
