# Supplementary figures and images for: Prognostic factors for favorable outcomes after veno-venous extracorporeal membrane oxygenation in critical care patients with COVID-19
Source: PLoS One. 2023 Jan 20;18(1):e0280502. doi: 10.1371/journal.pone.0280502 (PMC9858373; doi:10.1371/journal.pone.0280502)

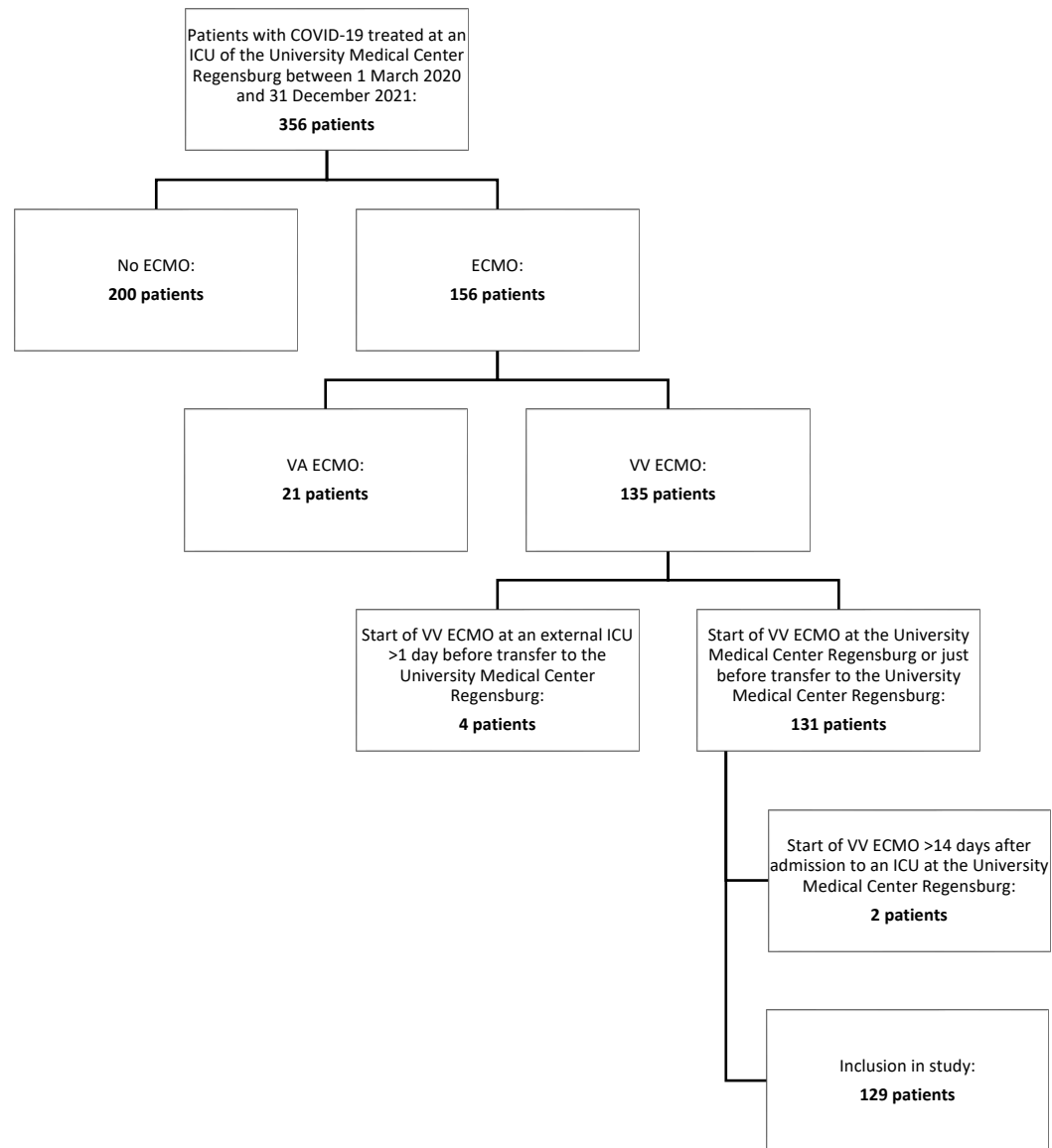

Supplement: S1 Fig — (PDF) [file pone.0280502.s006.pdf]

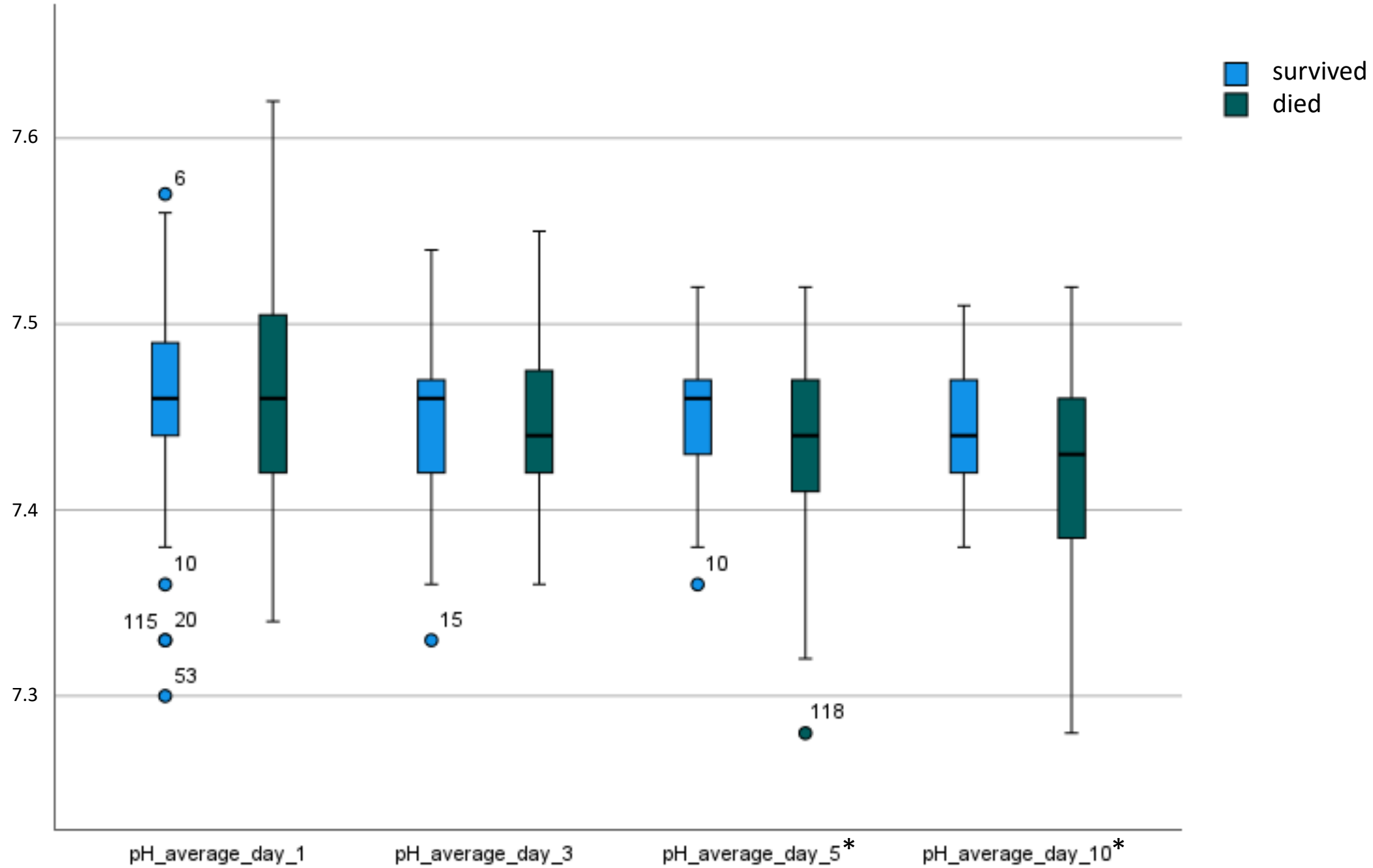

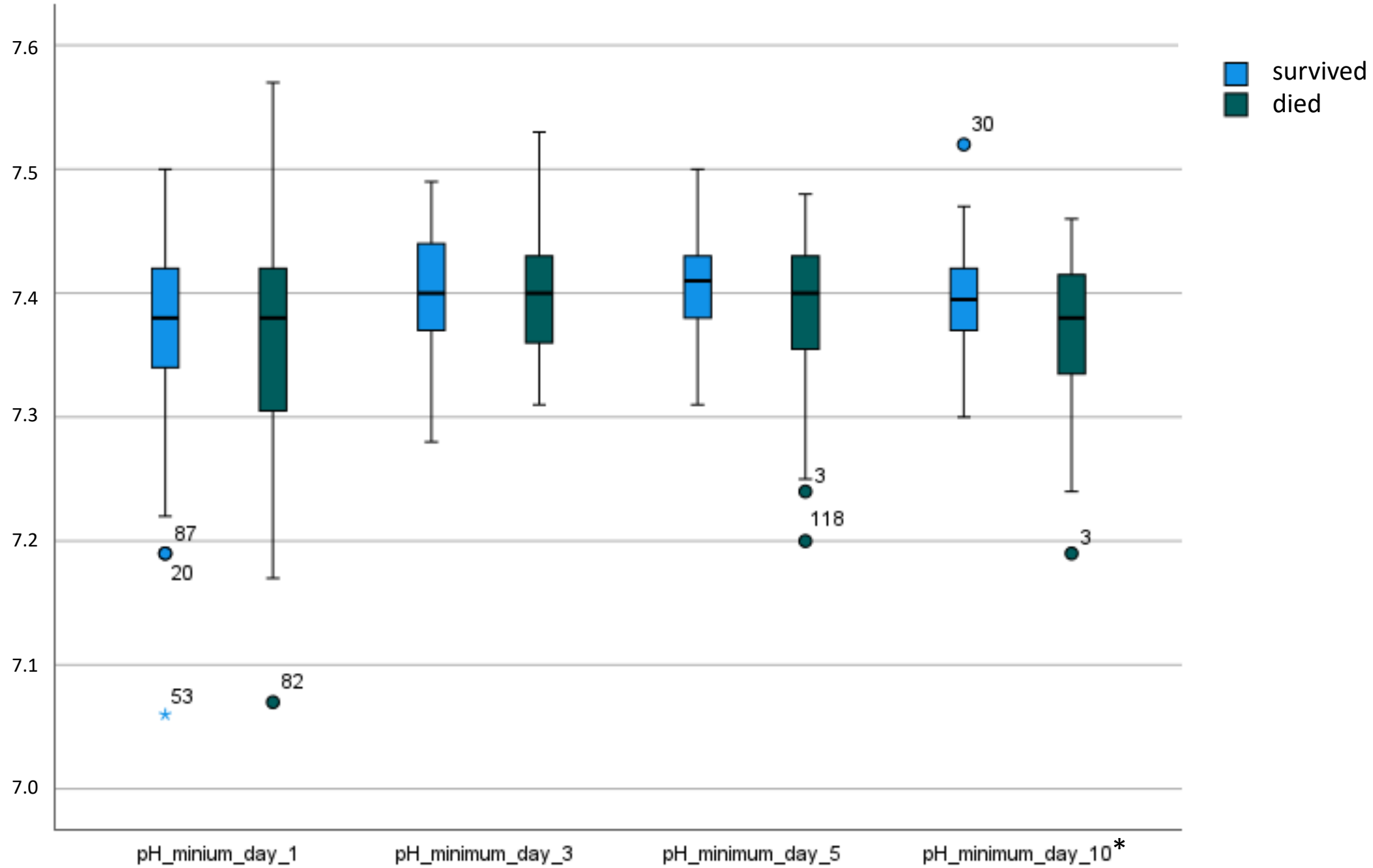

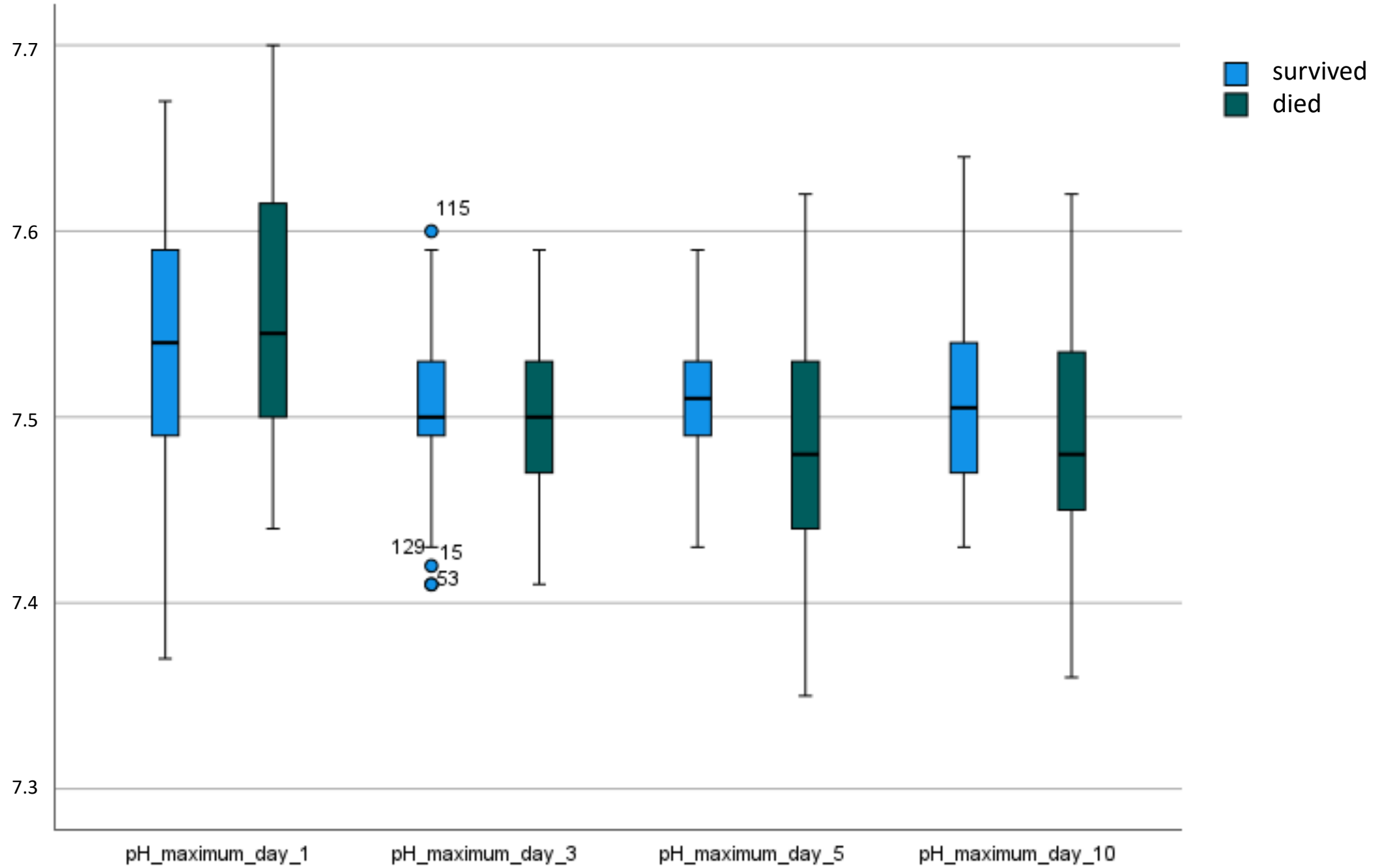

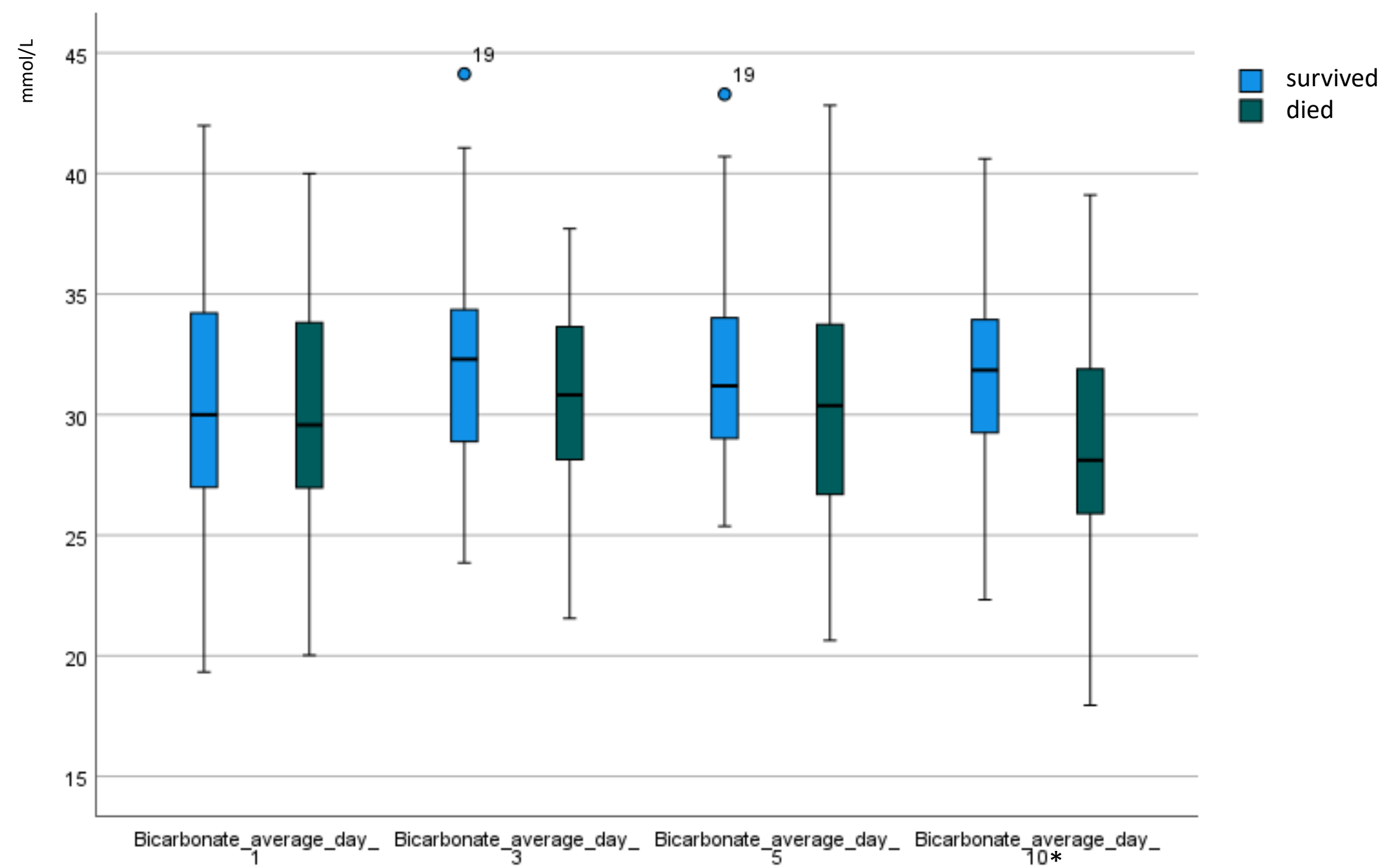

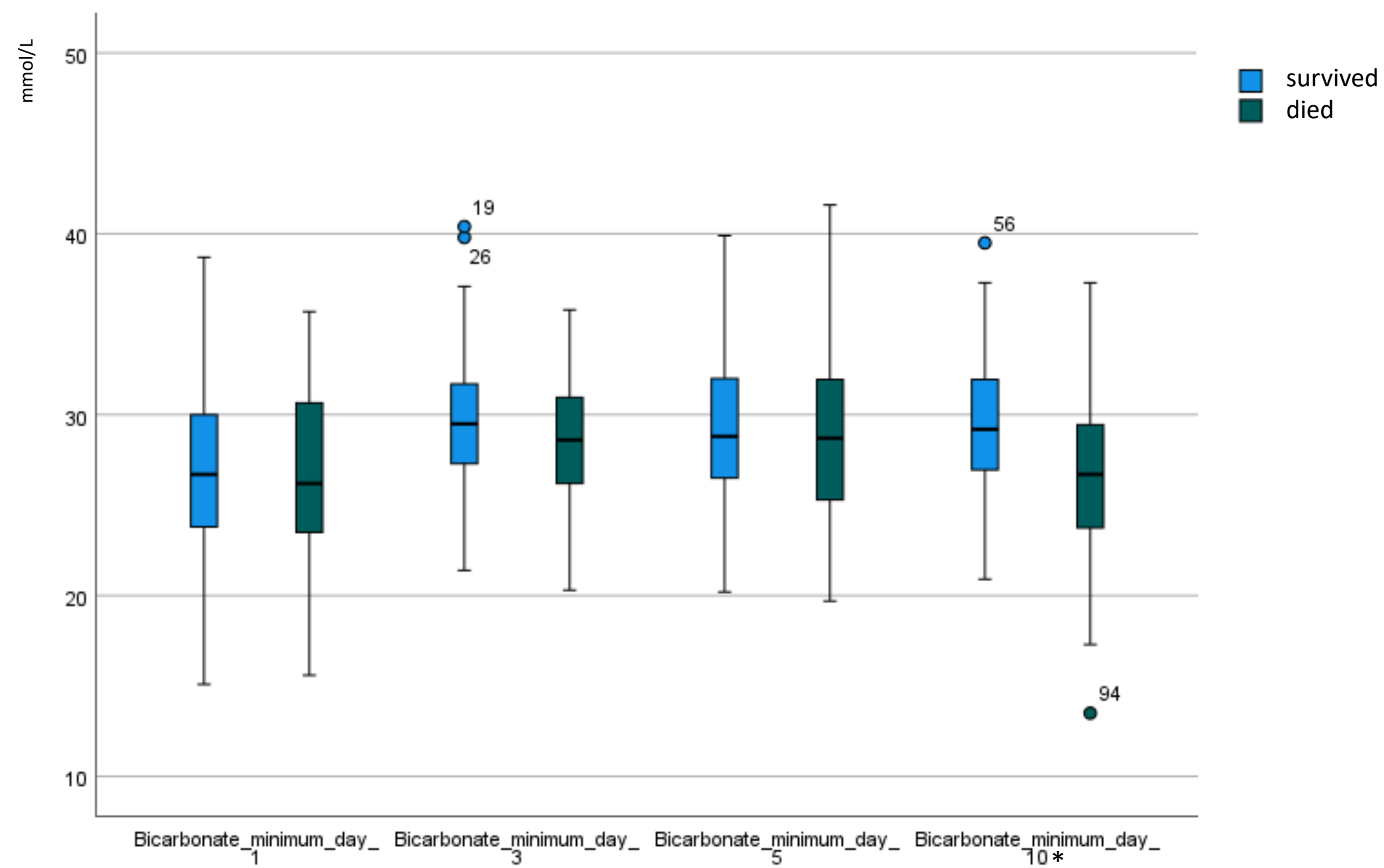

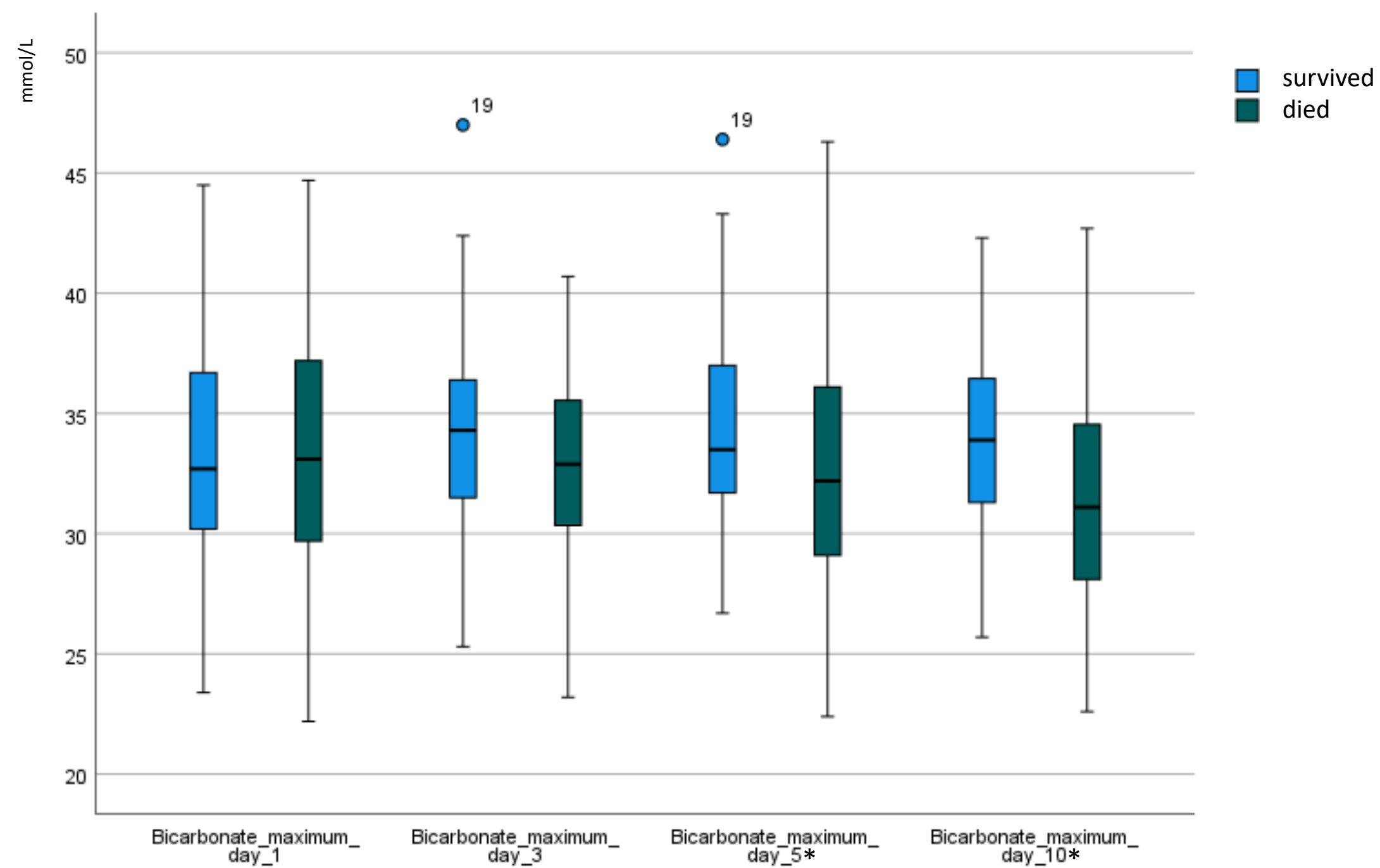

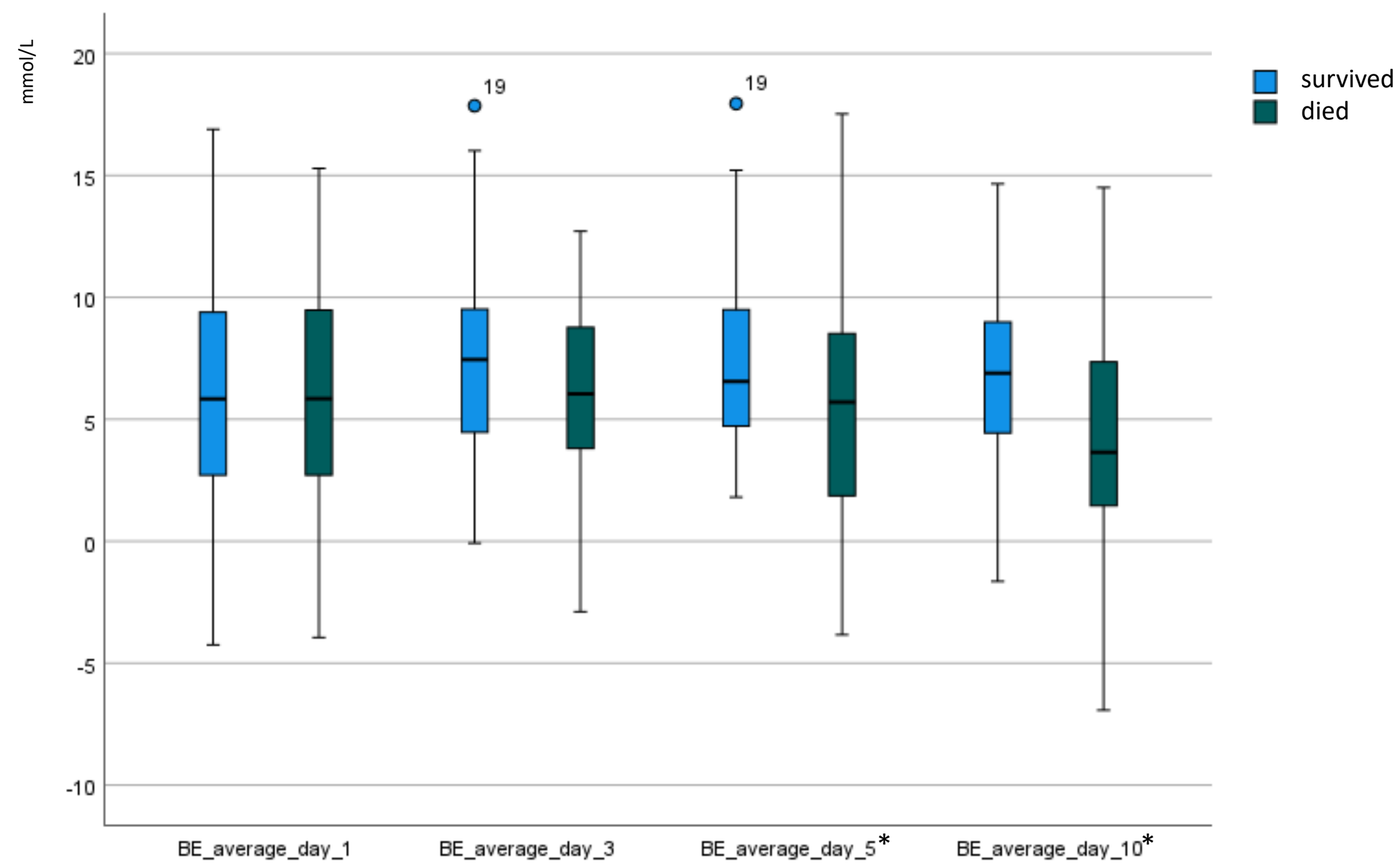

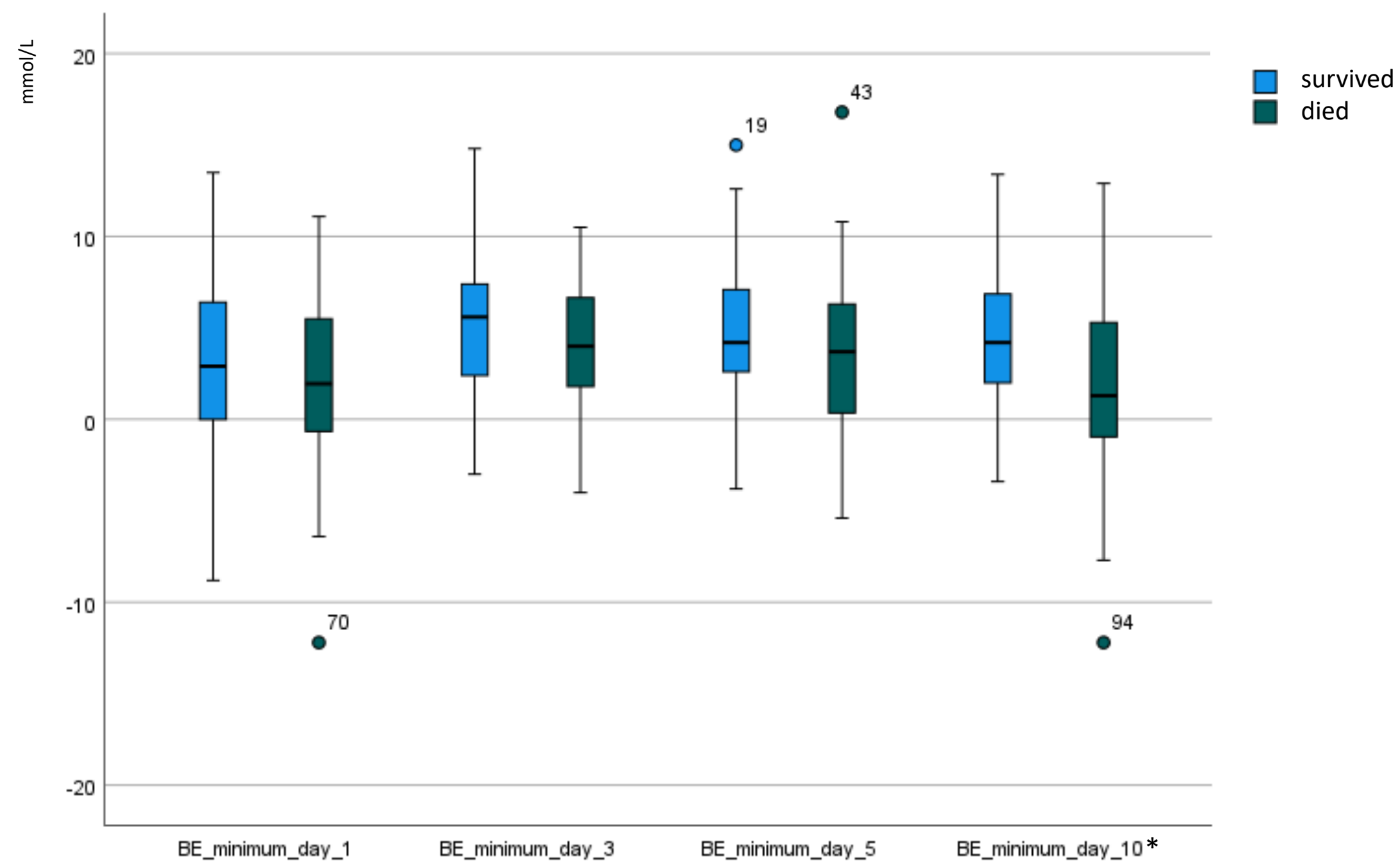

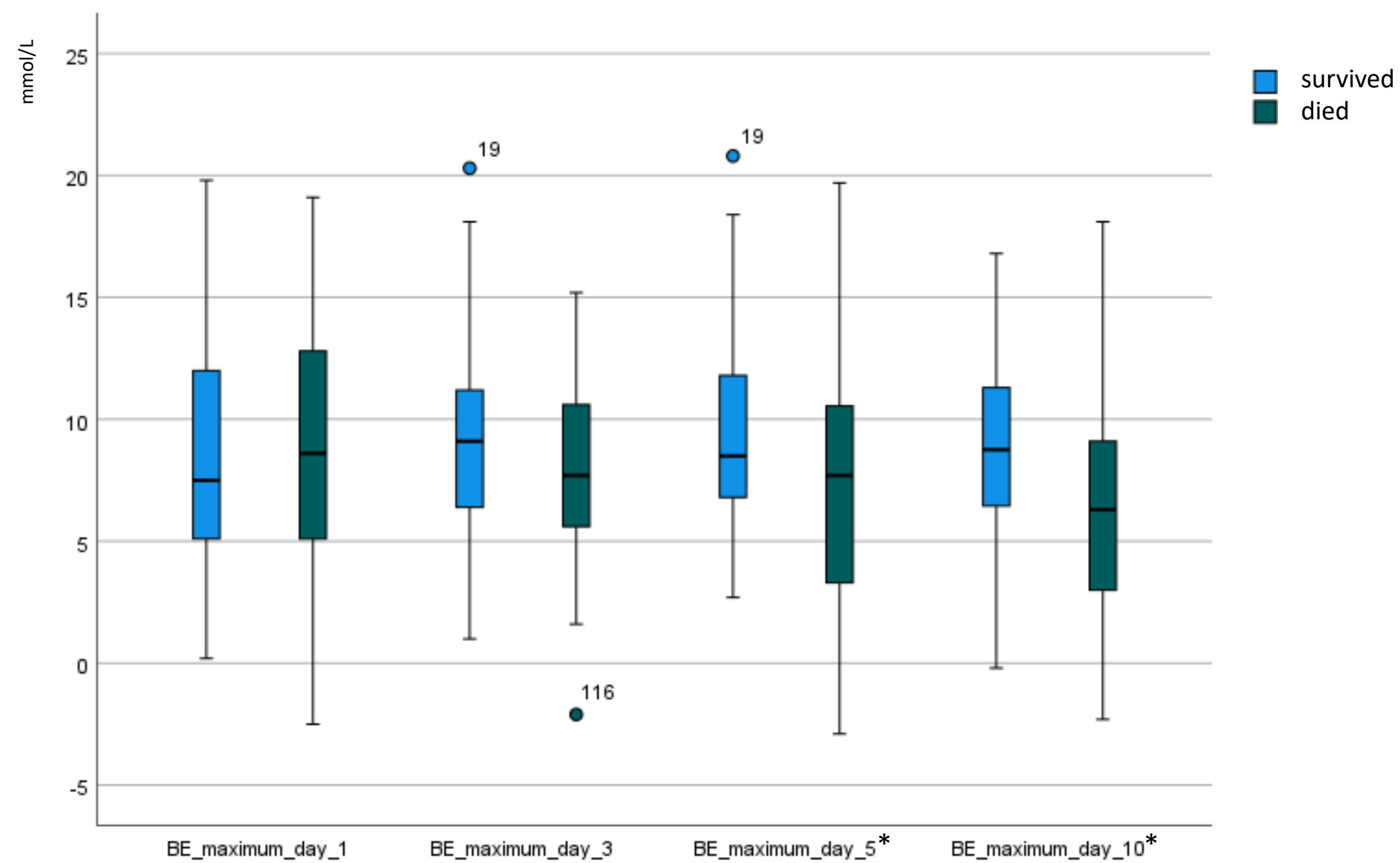

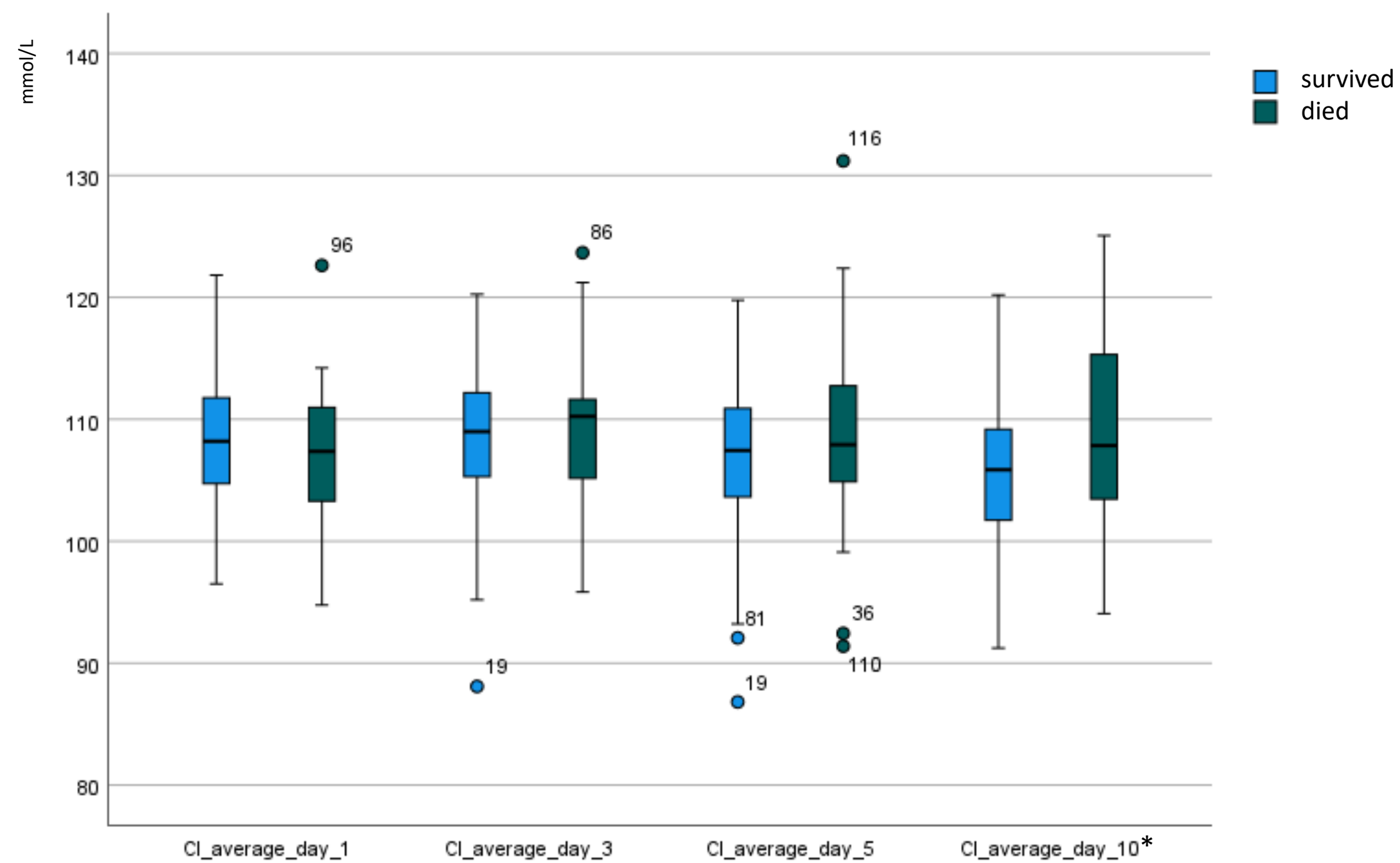

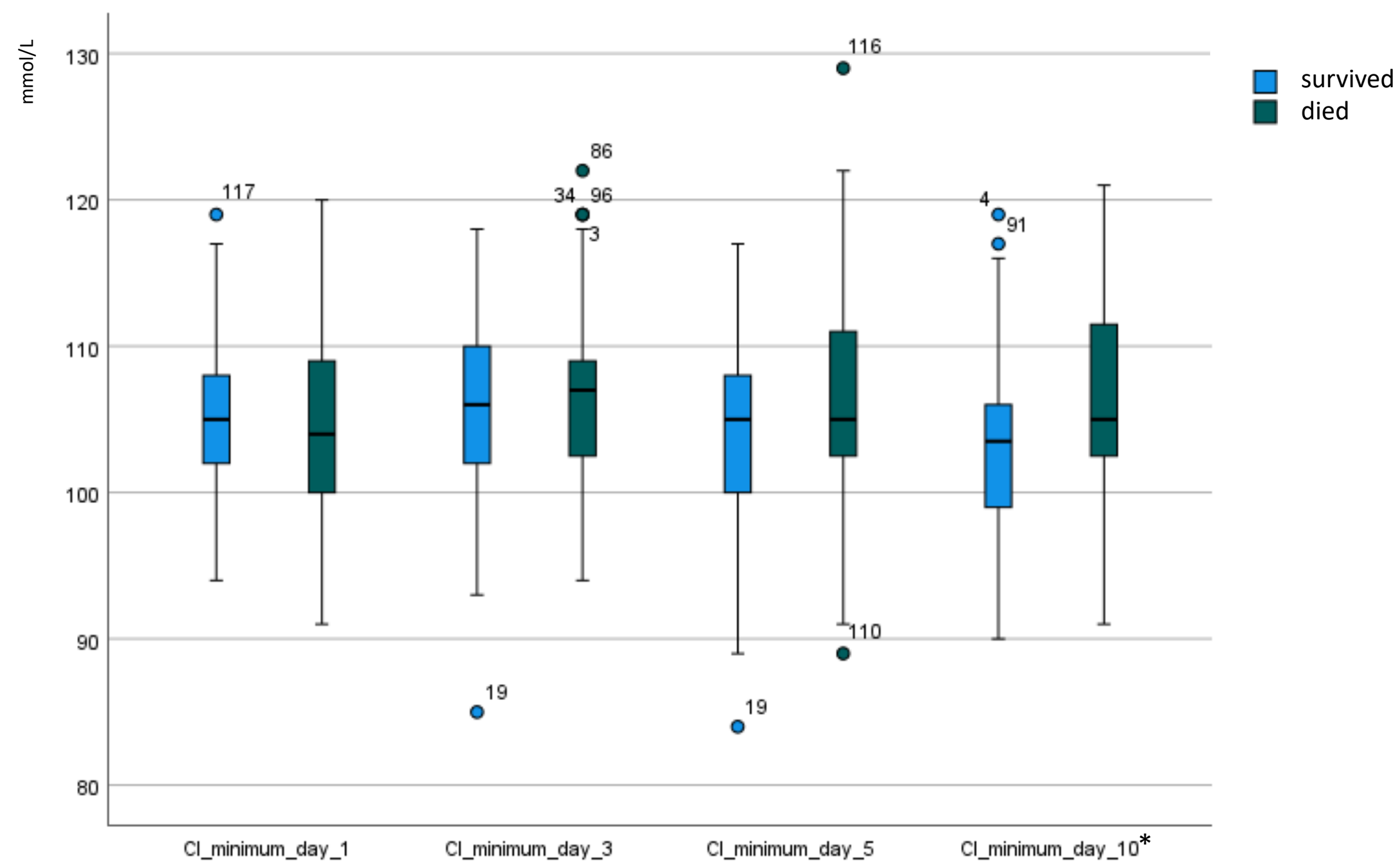

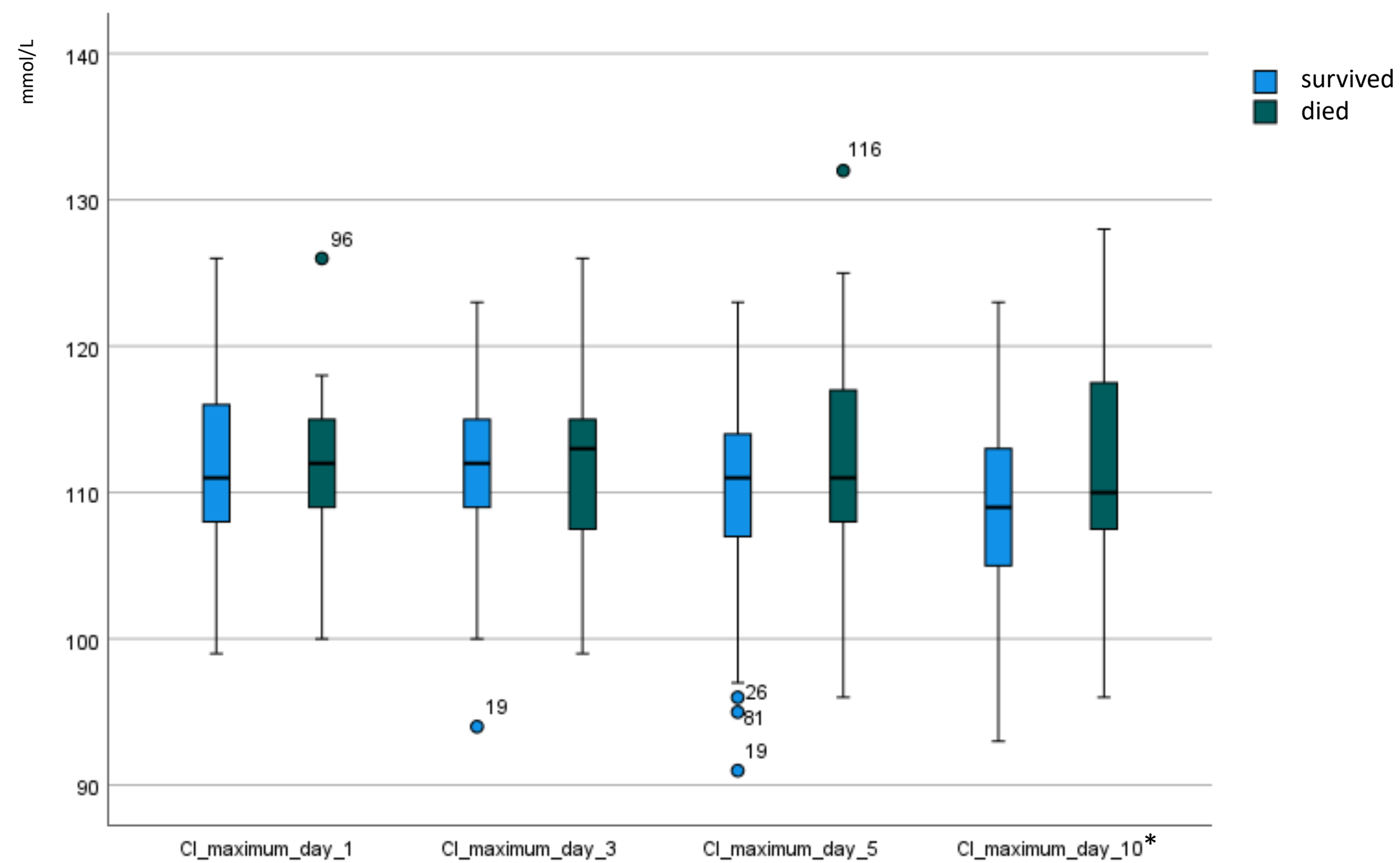

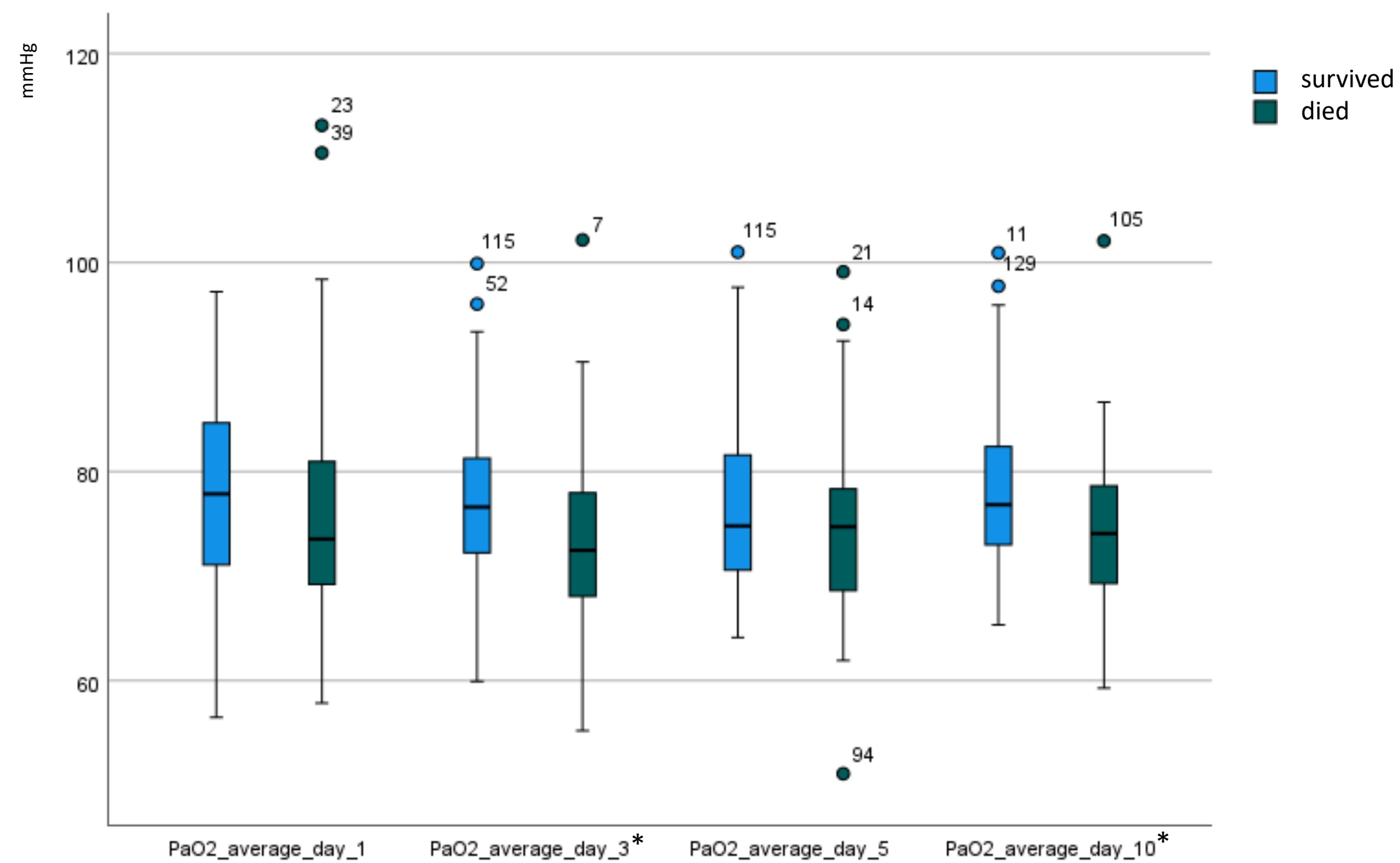

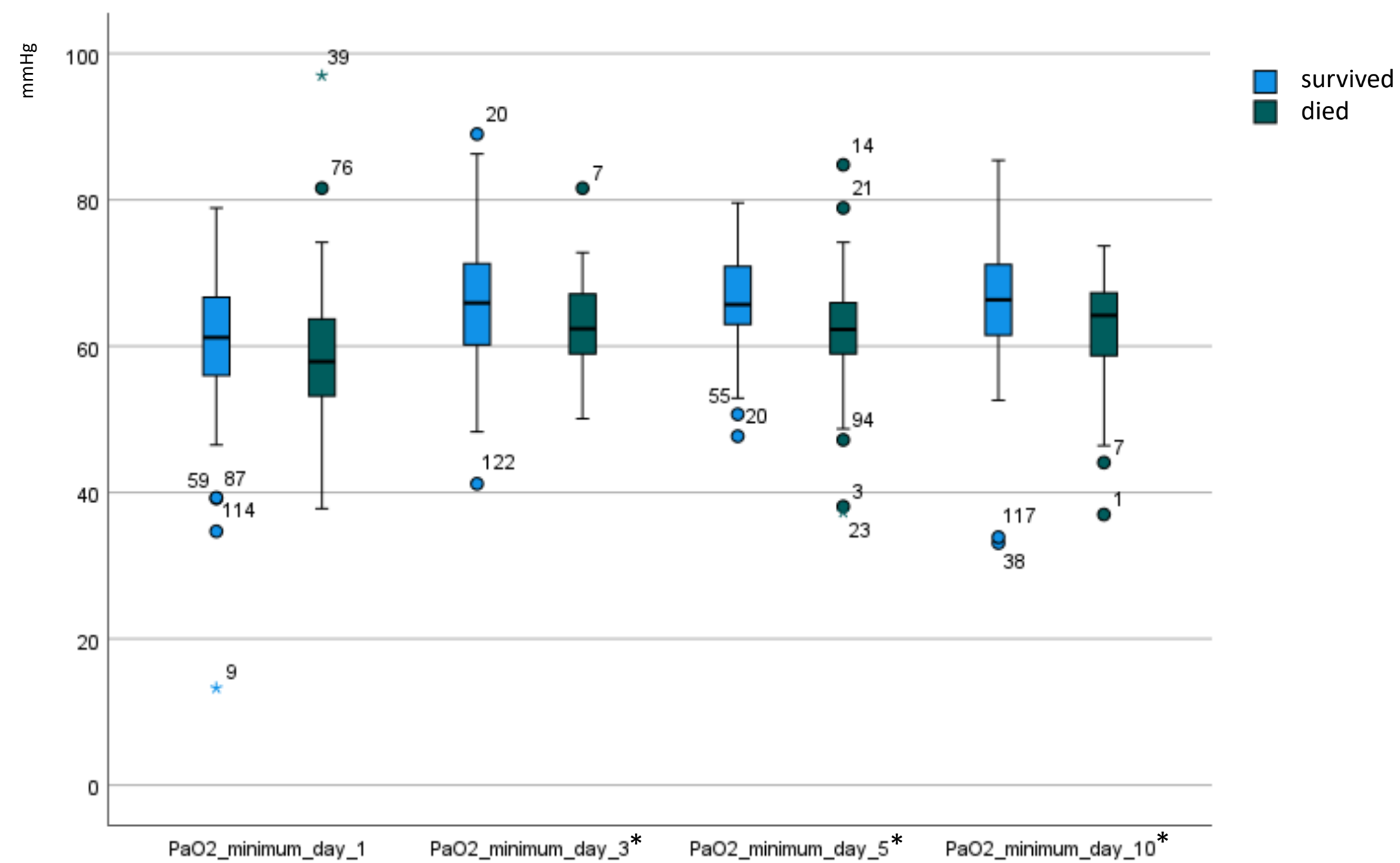

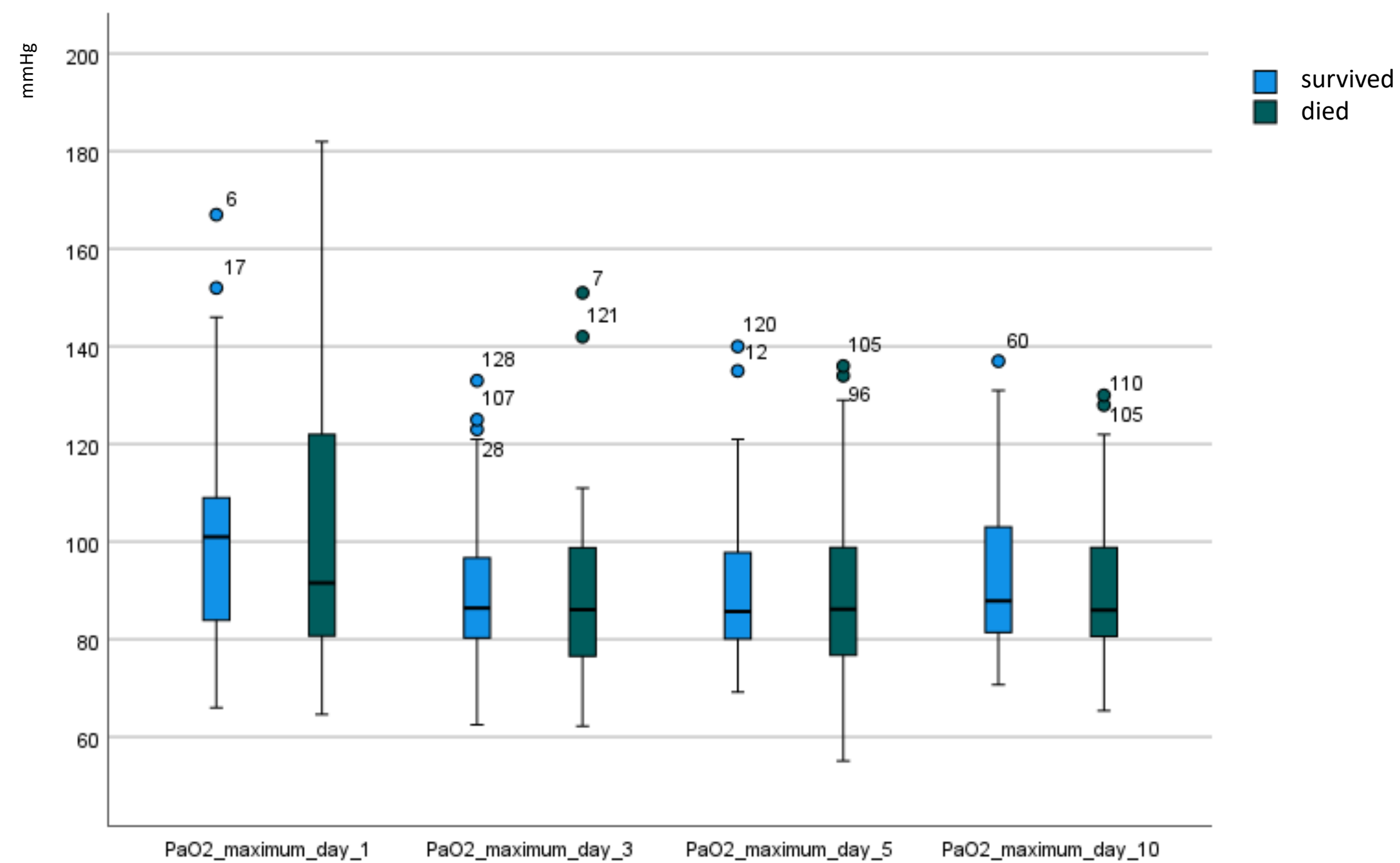

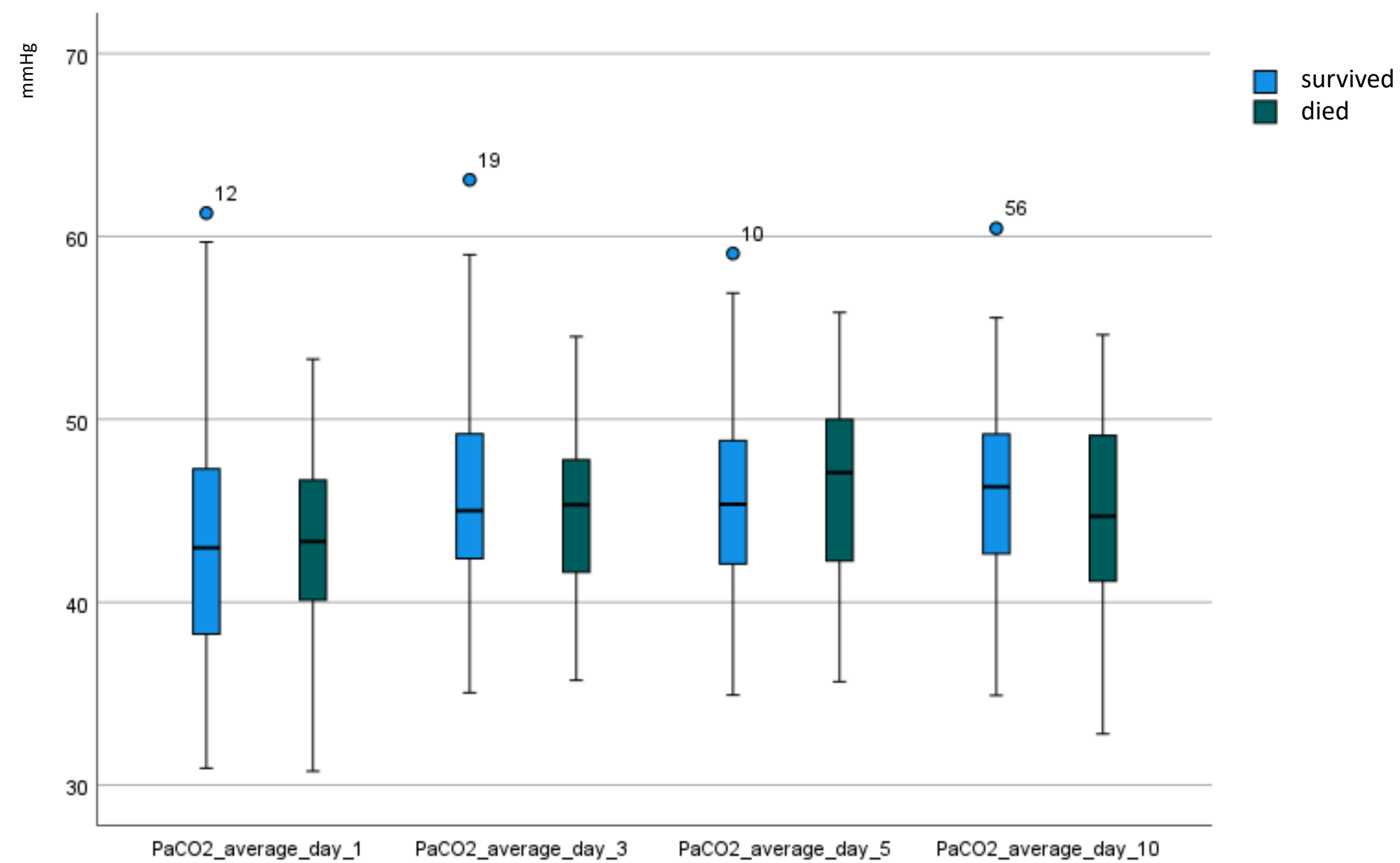

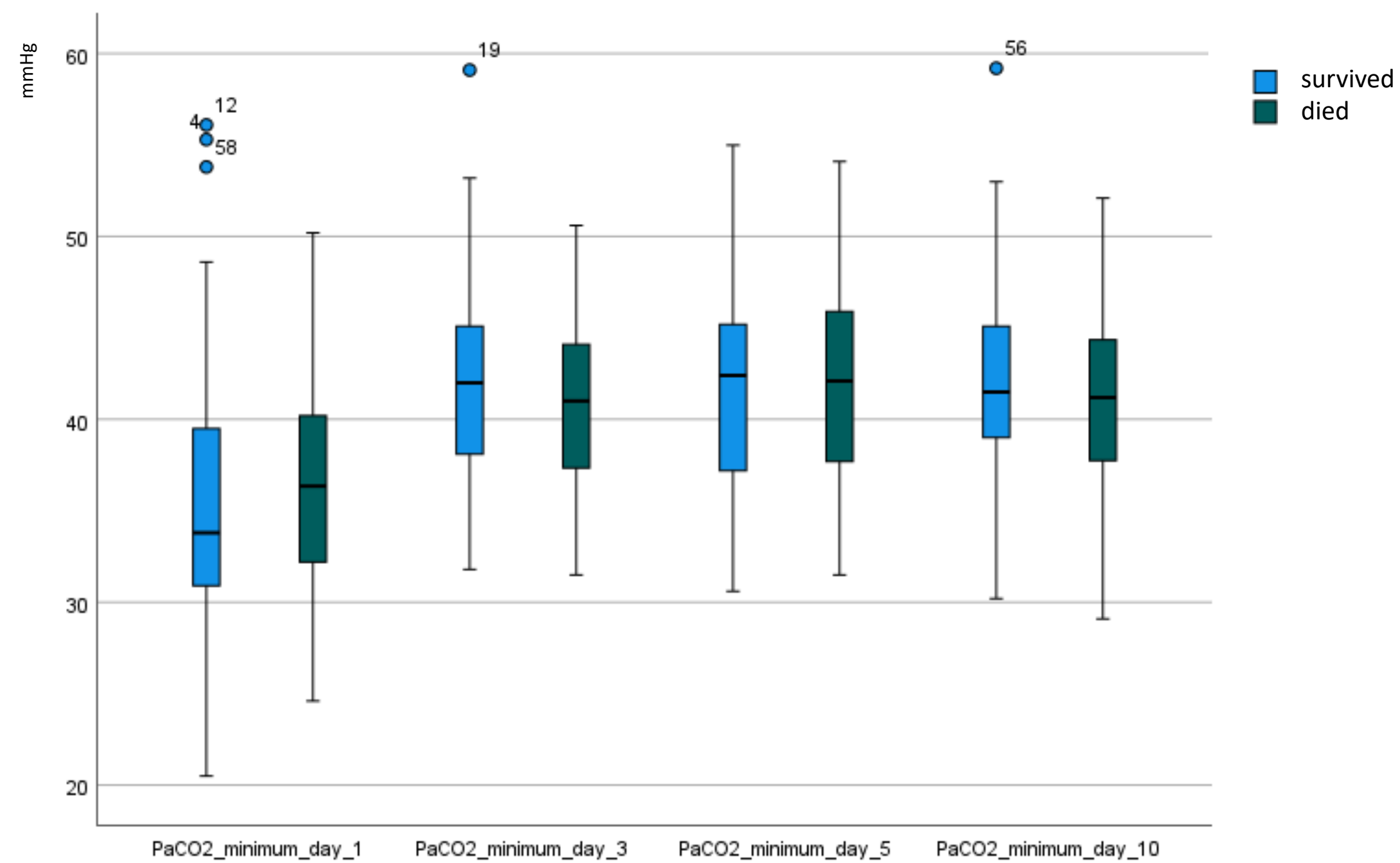

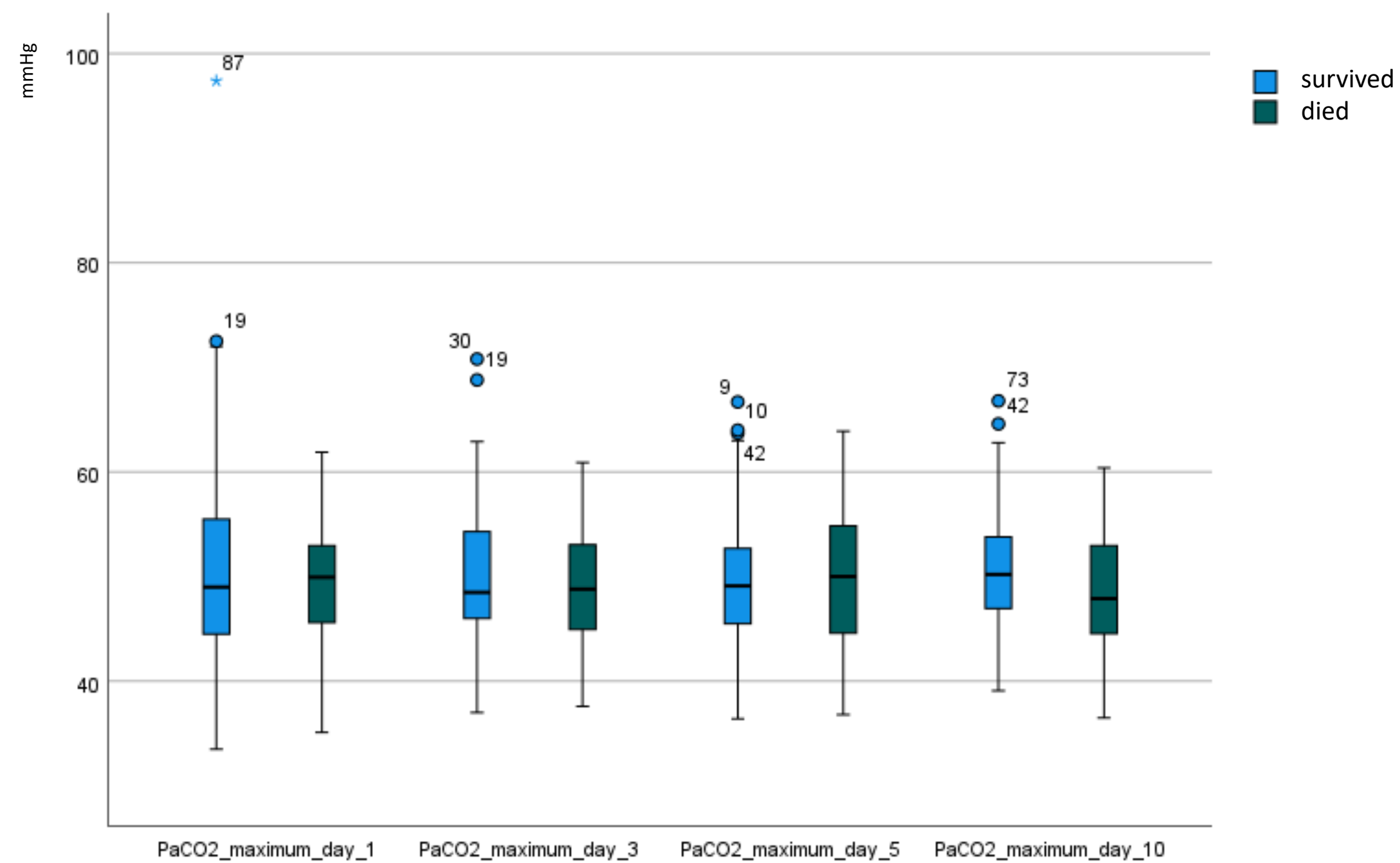

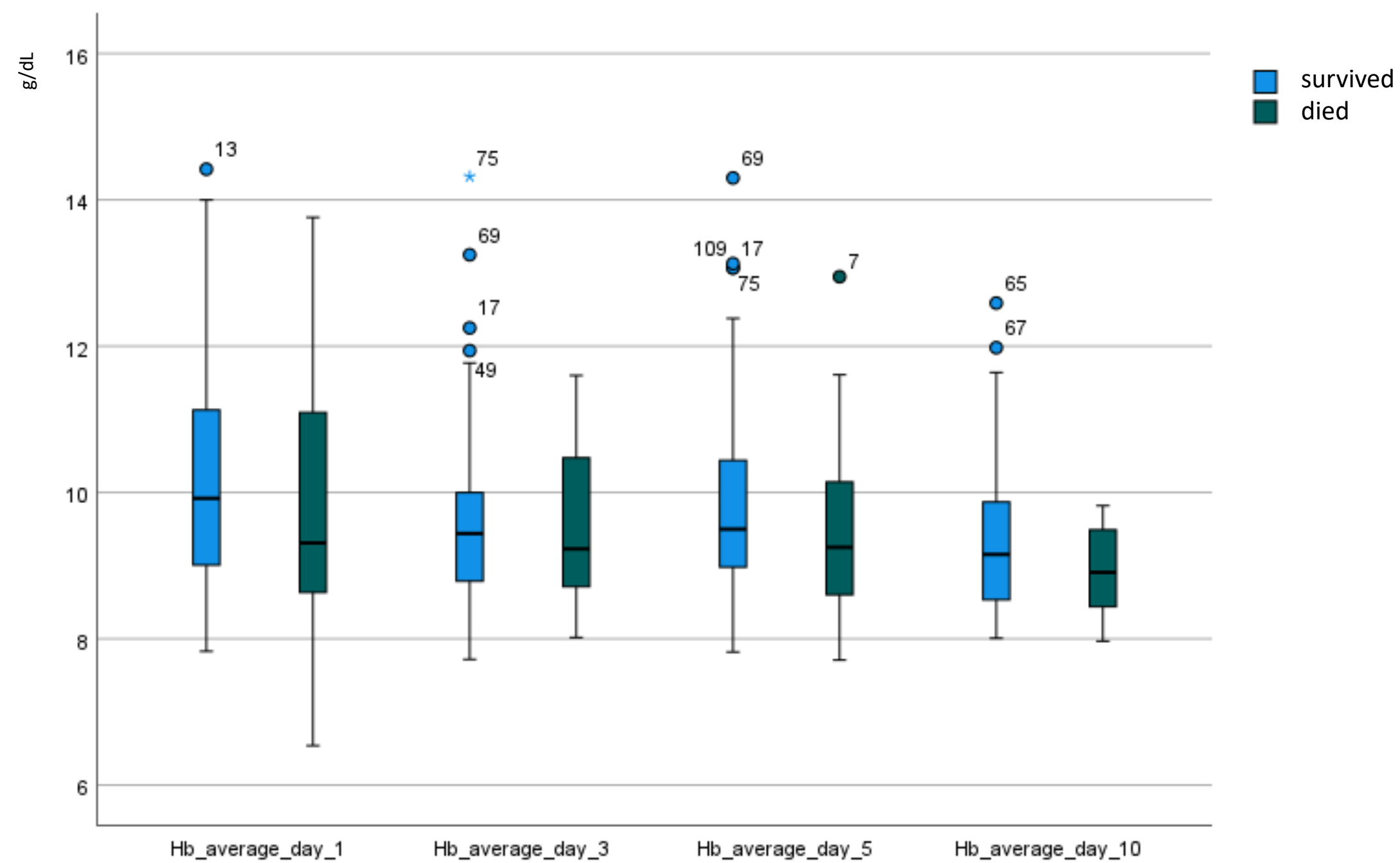

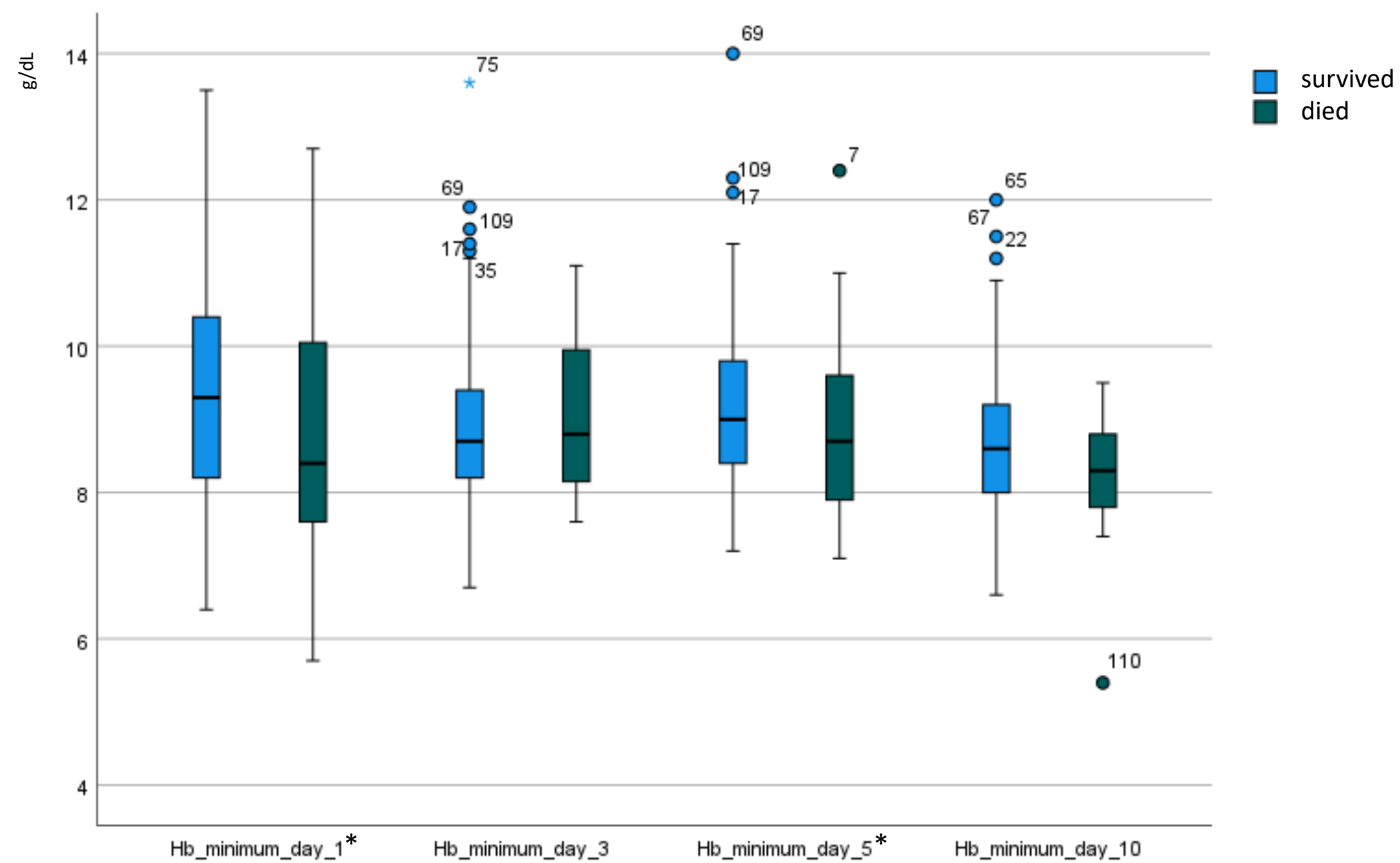

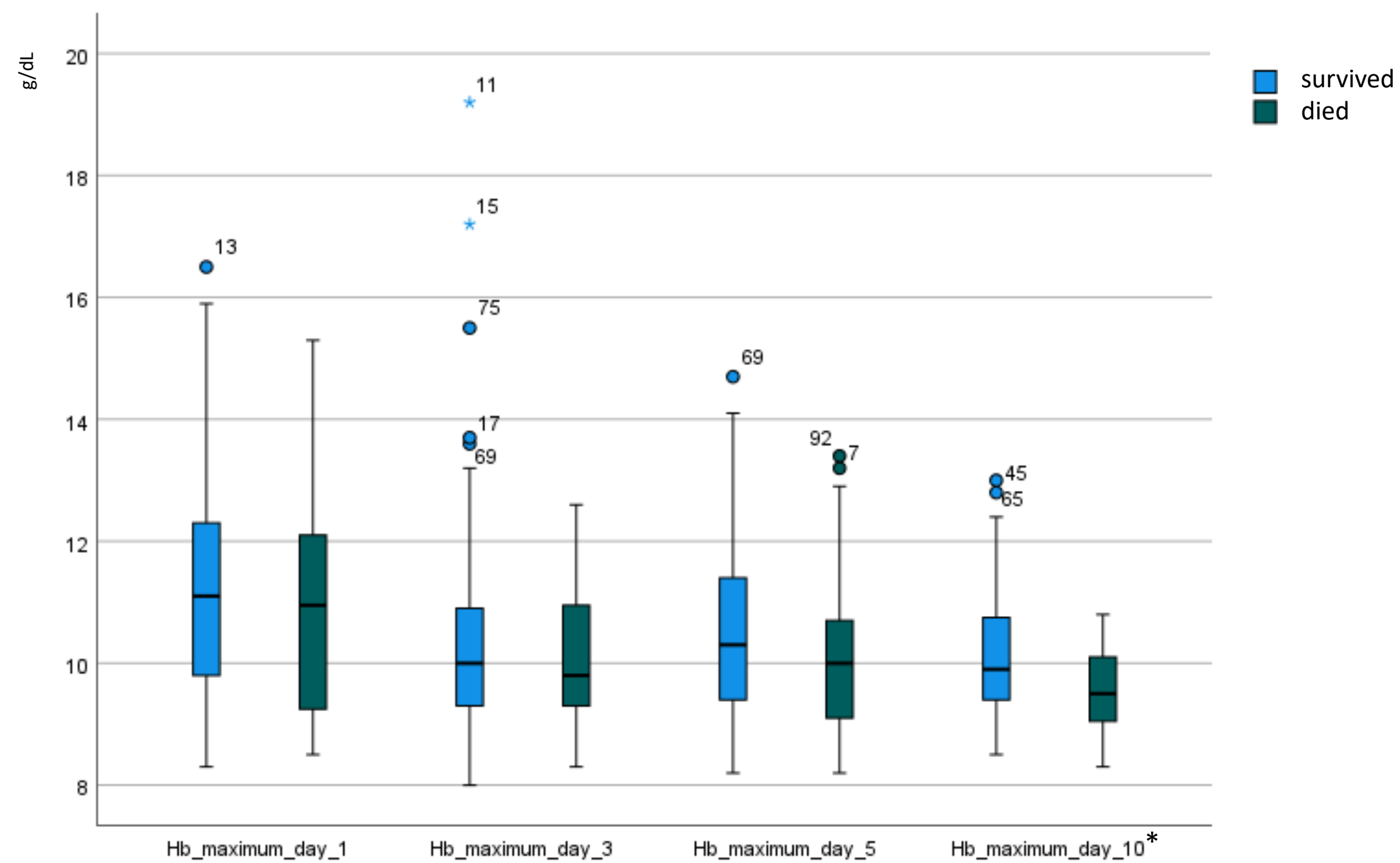

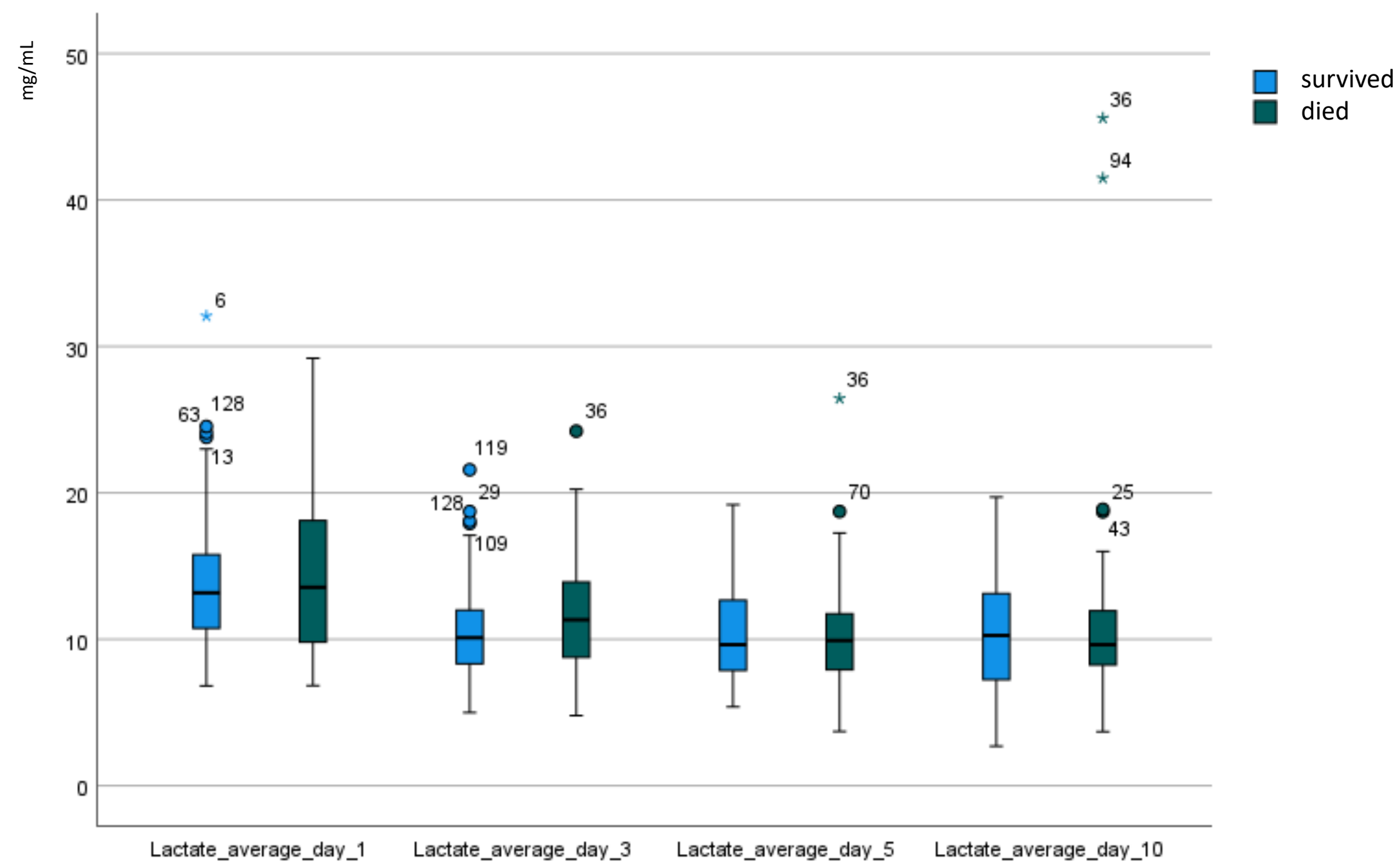

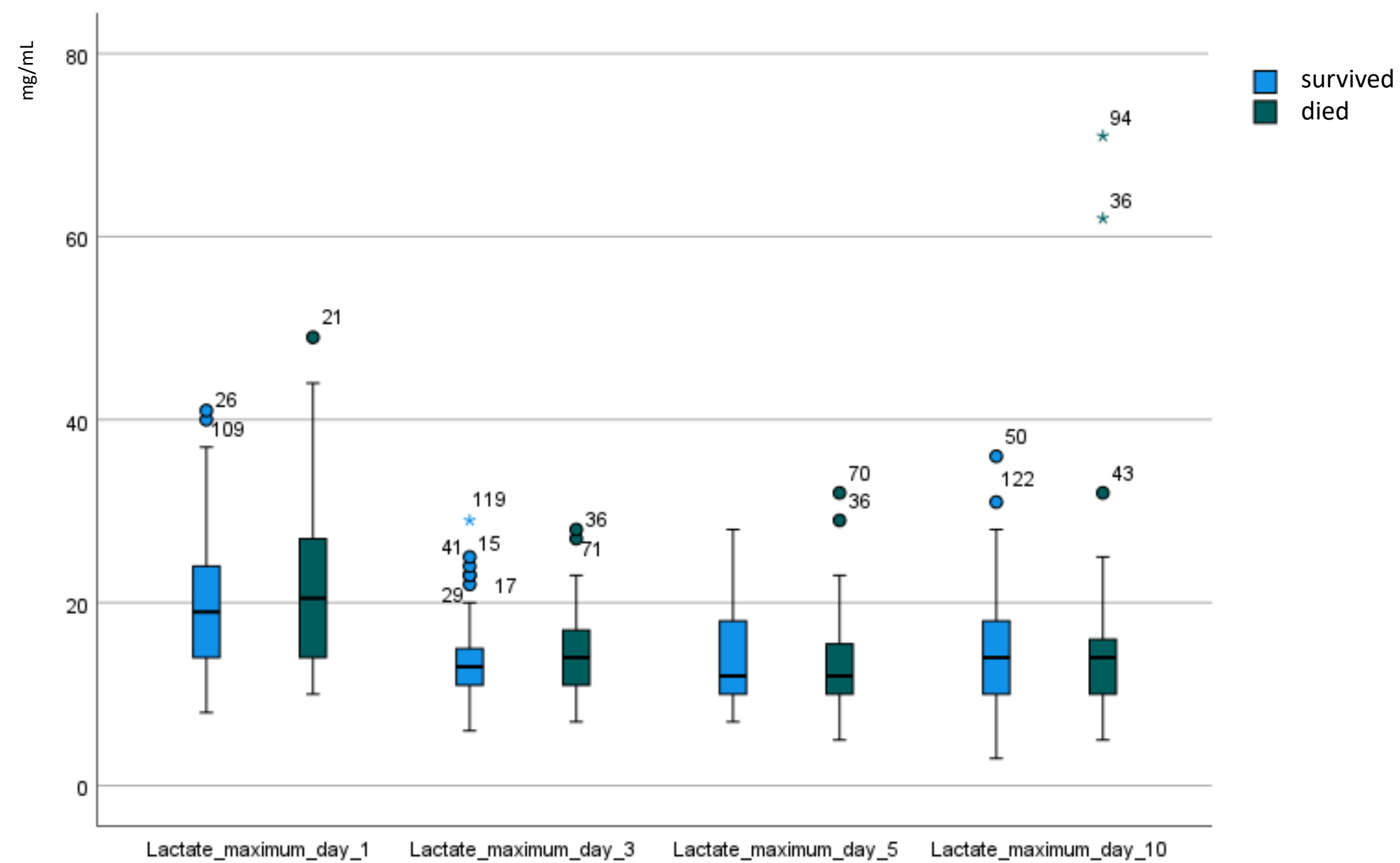

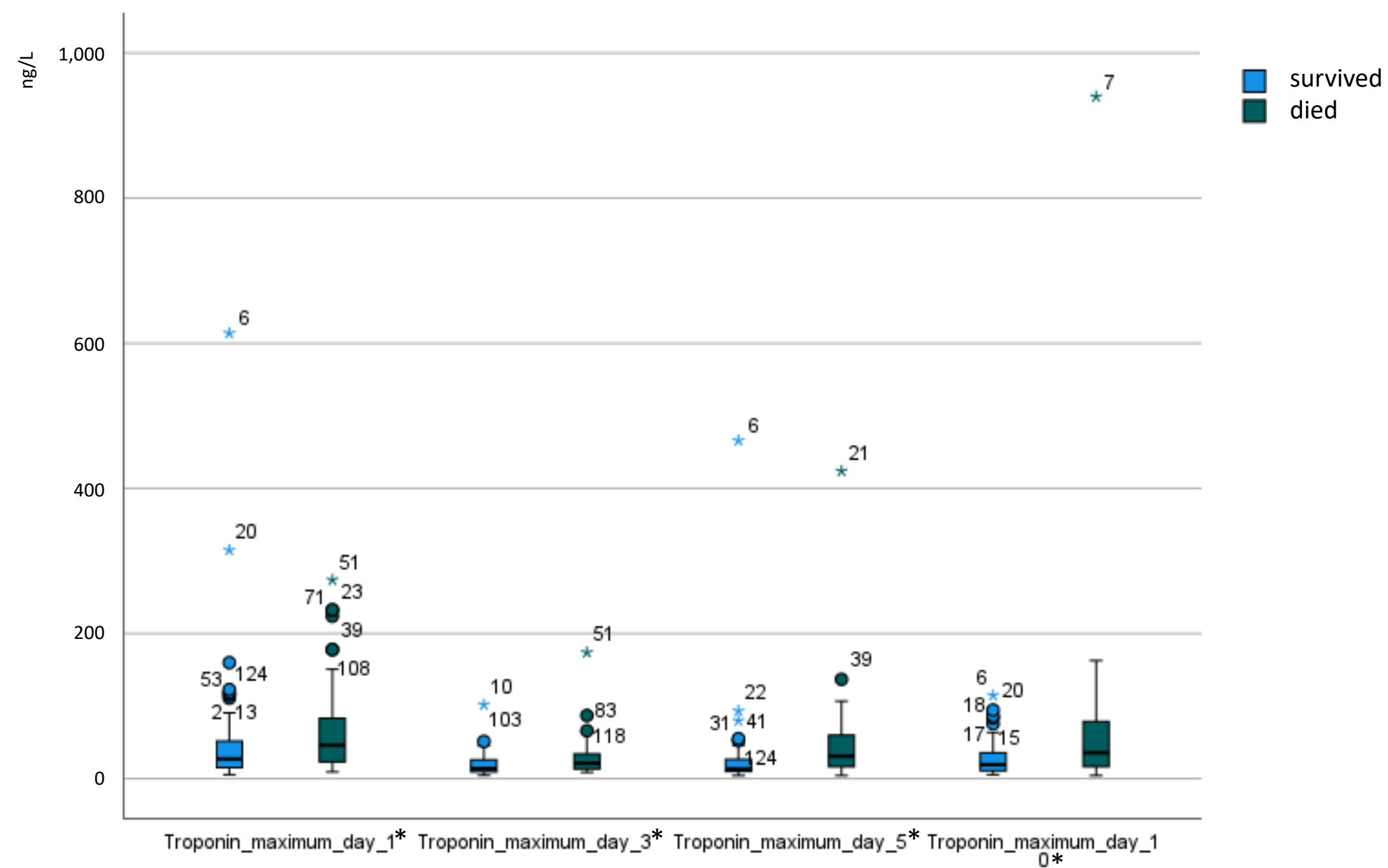

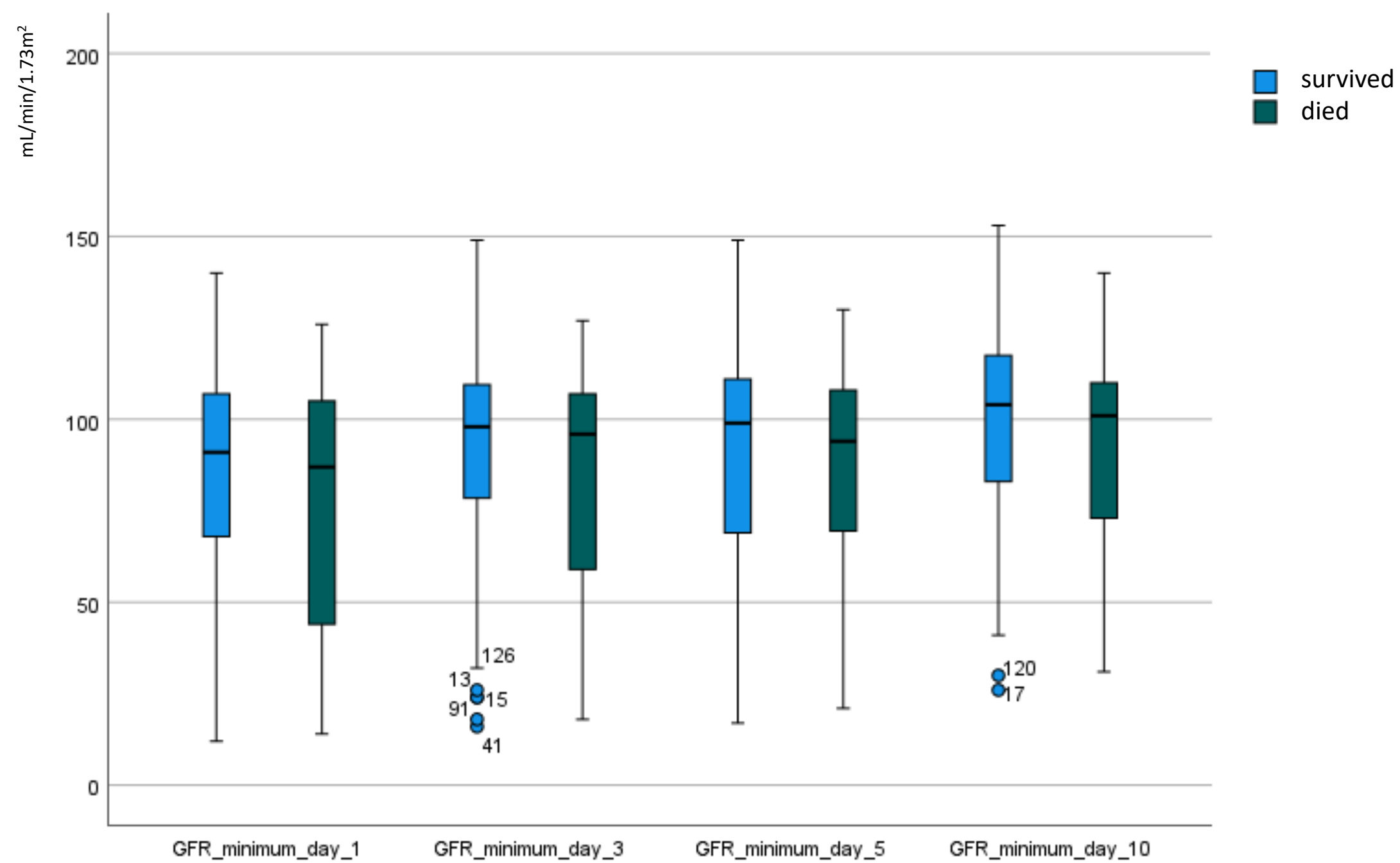

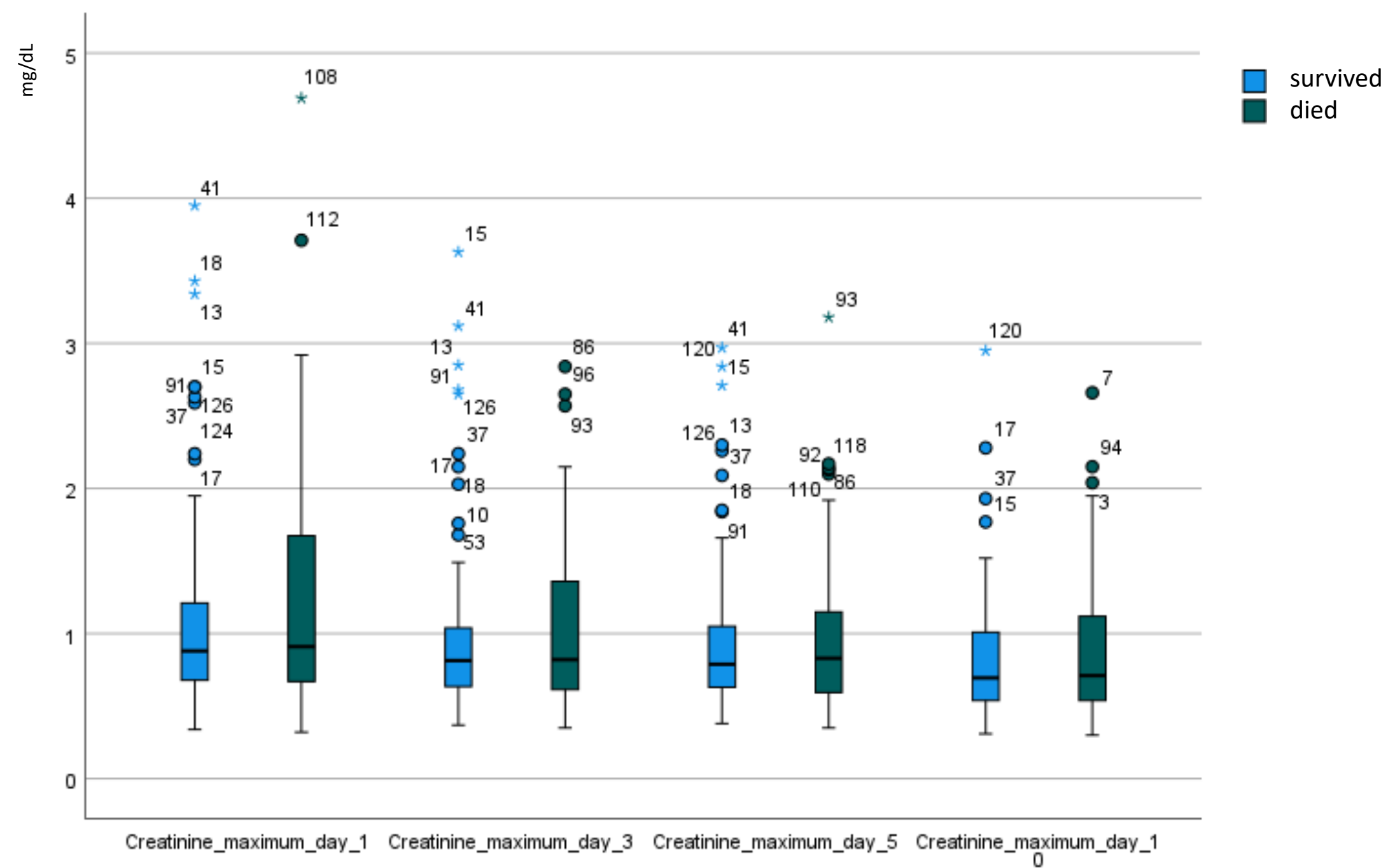

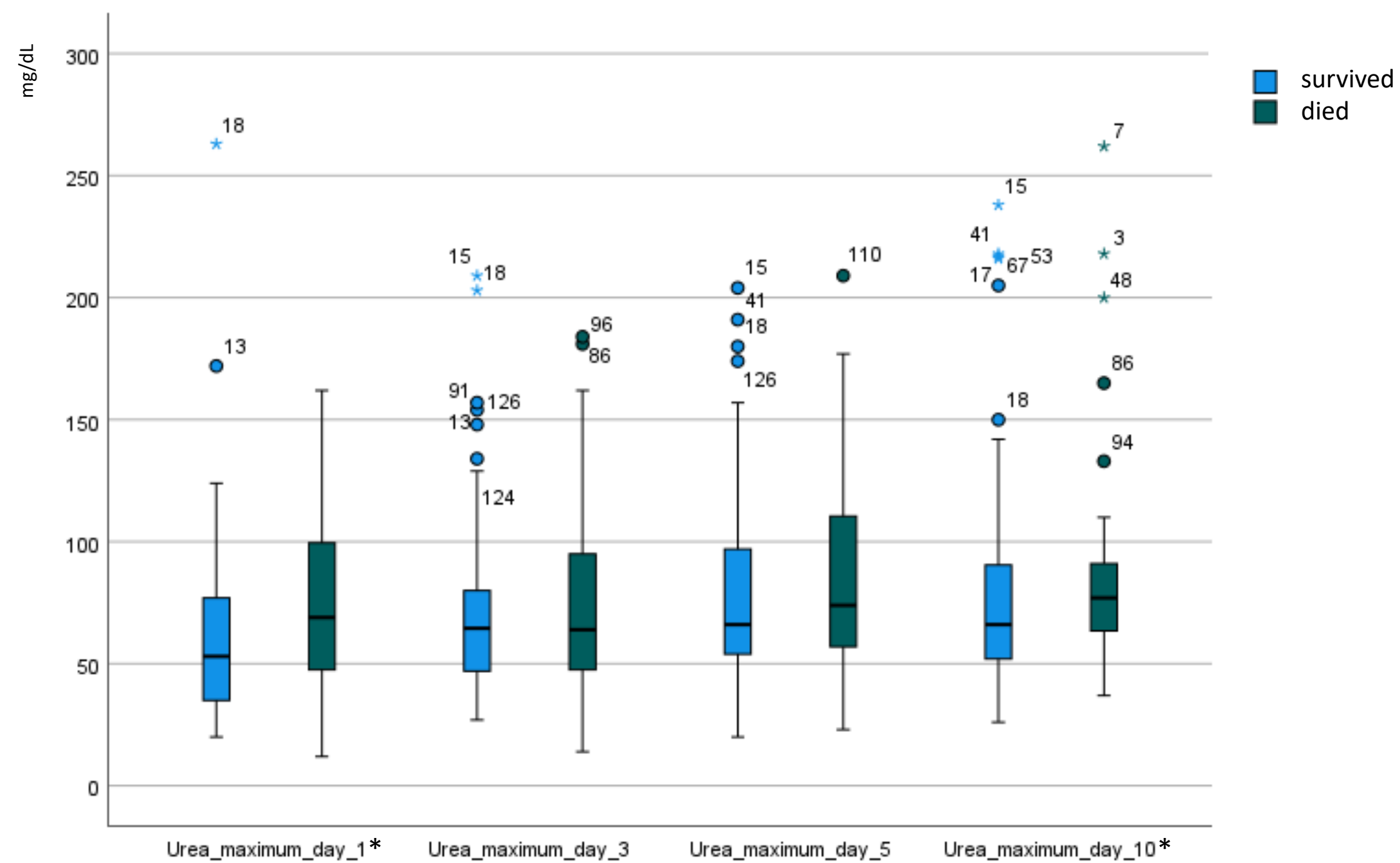

U/L

12,500

10,000

7,500

5,000

2,500

0

■ survived  
■ died

107 47  
124 109  
41 72  
AST\_maximum\_day\_1

78  
107  
100  
AST\_maximum\_day\_3

107 103  
50 78  
120 84 56 117  
AST\_maximum\_day\_5

105  
34  
120 46  
4 98 1115  
AST\_maximum\_day\_10\*

94

\*

36

7

7

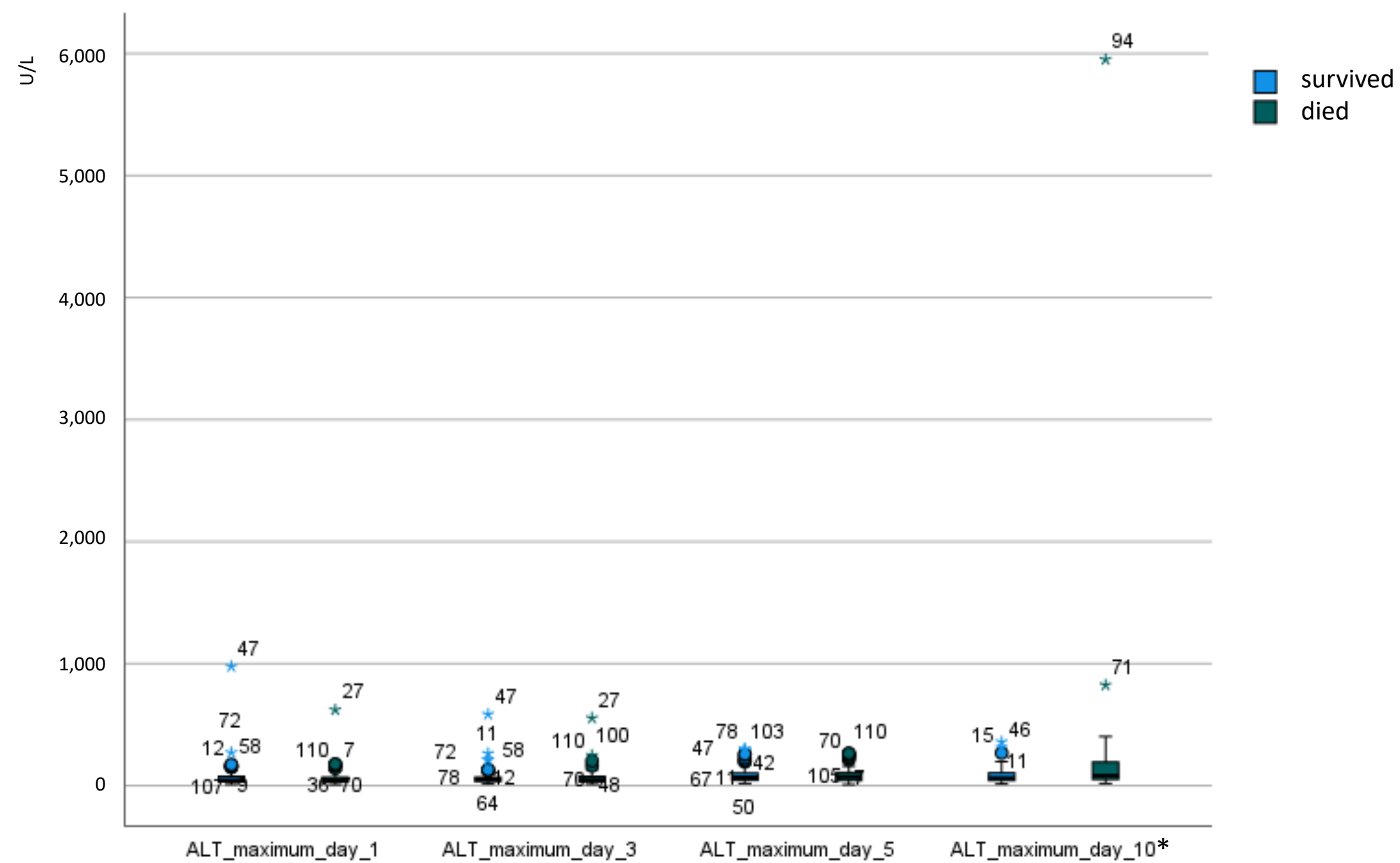

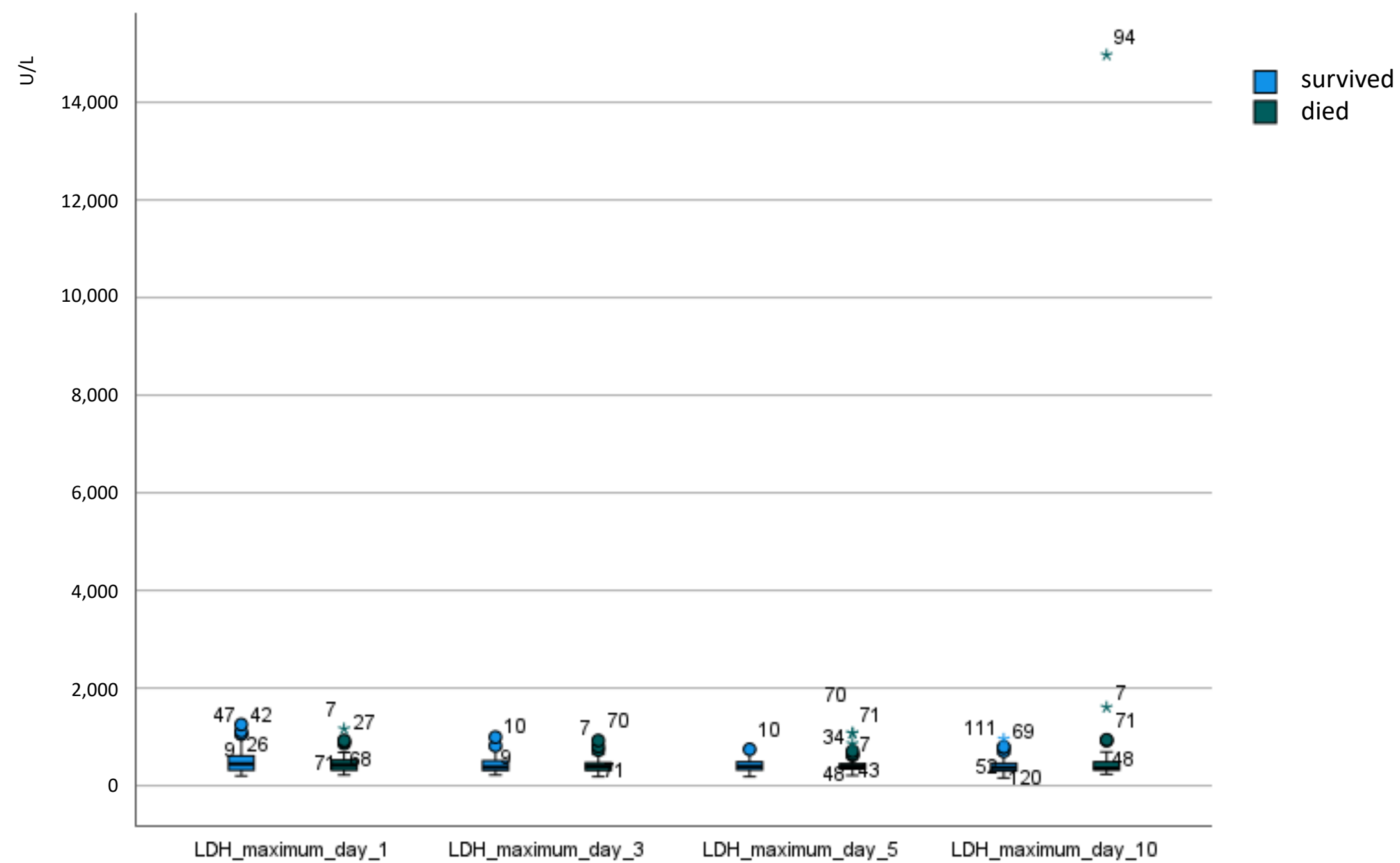

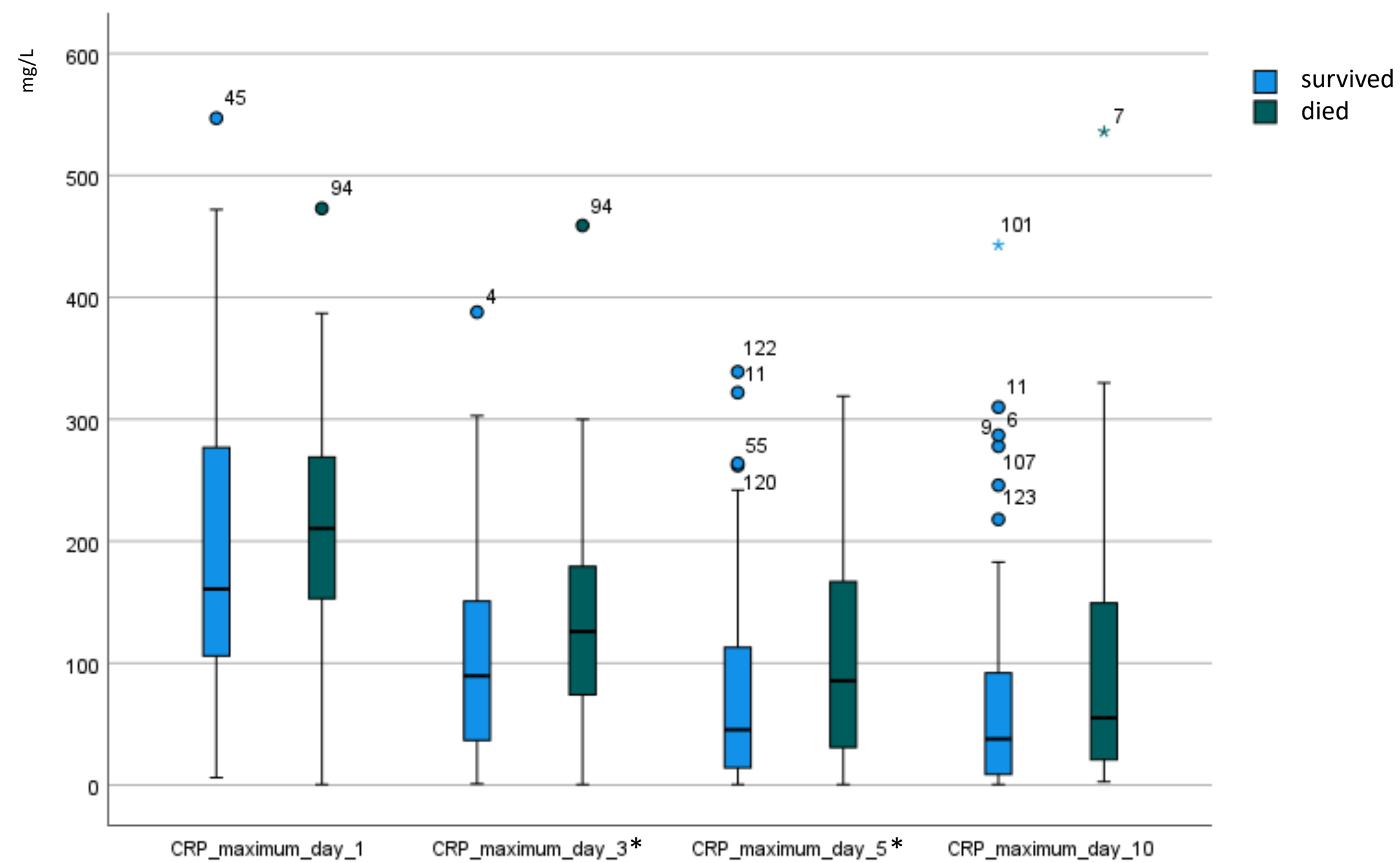

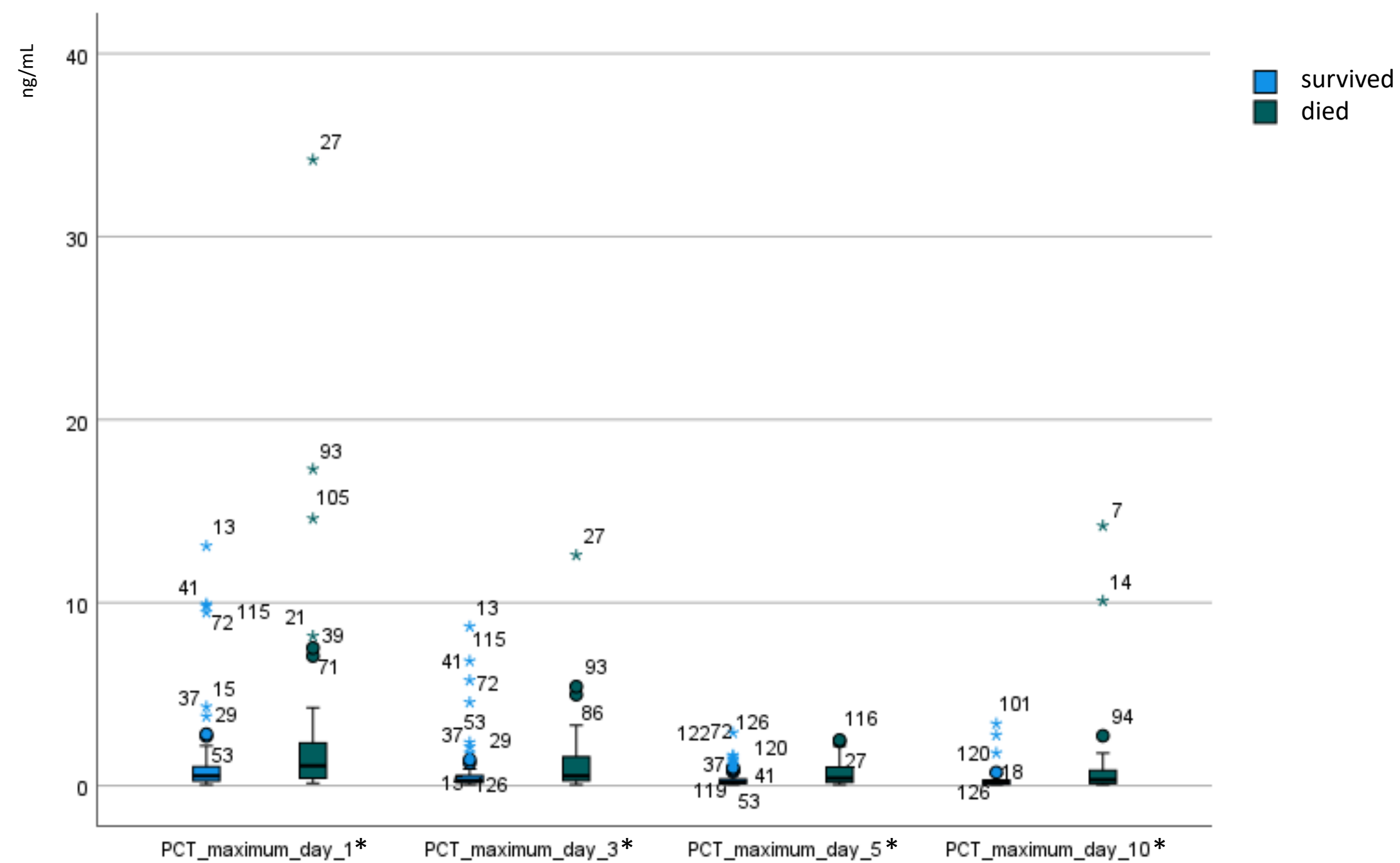

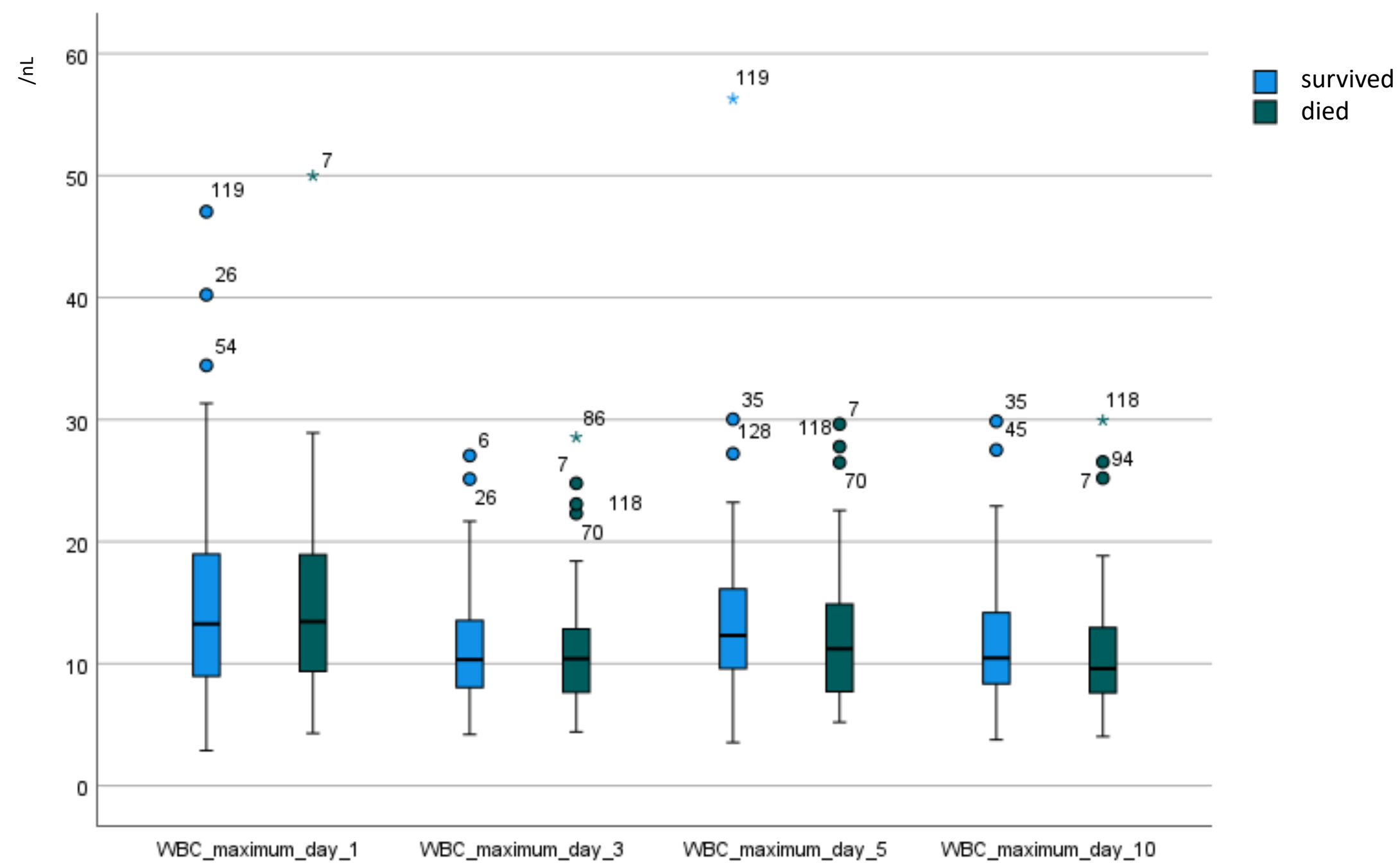

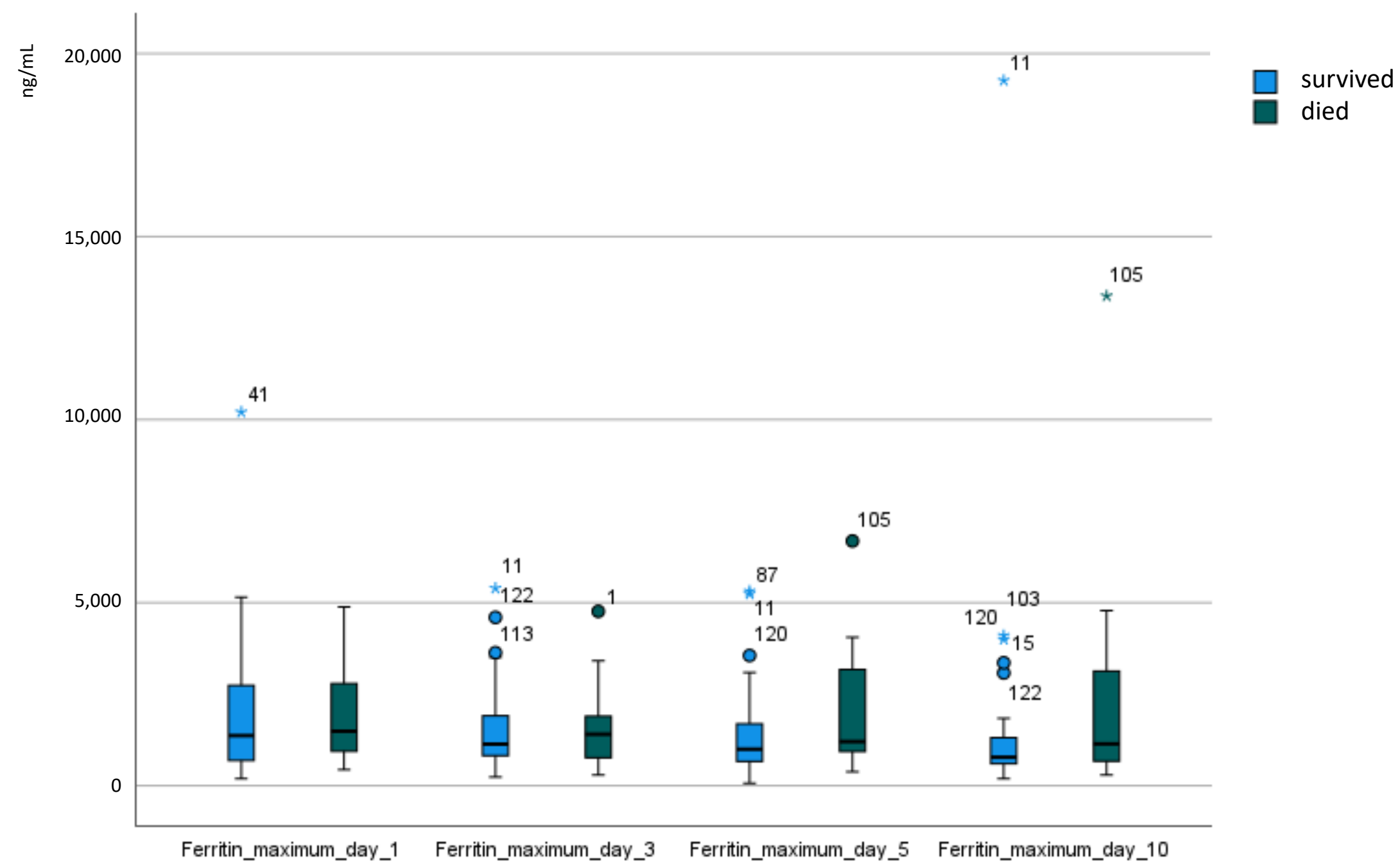

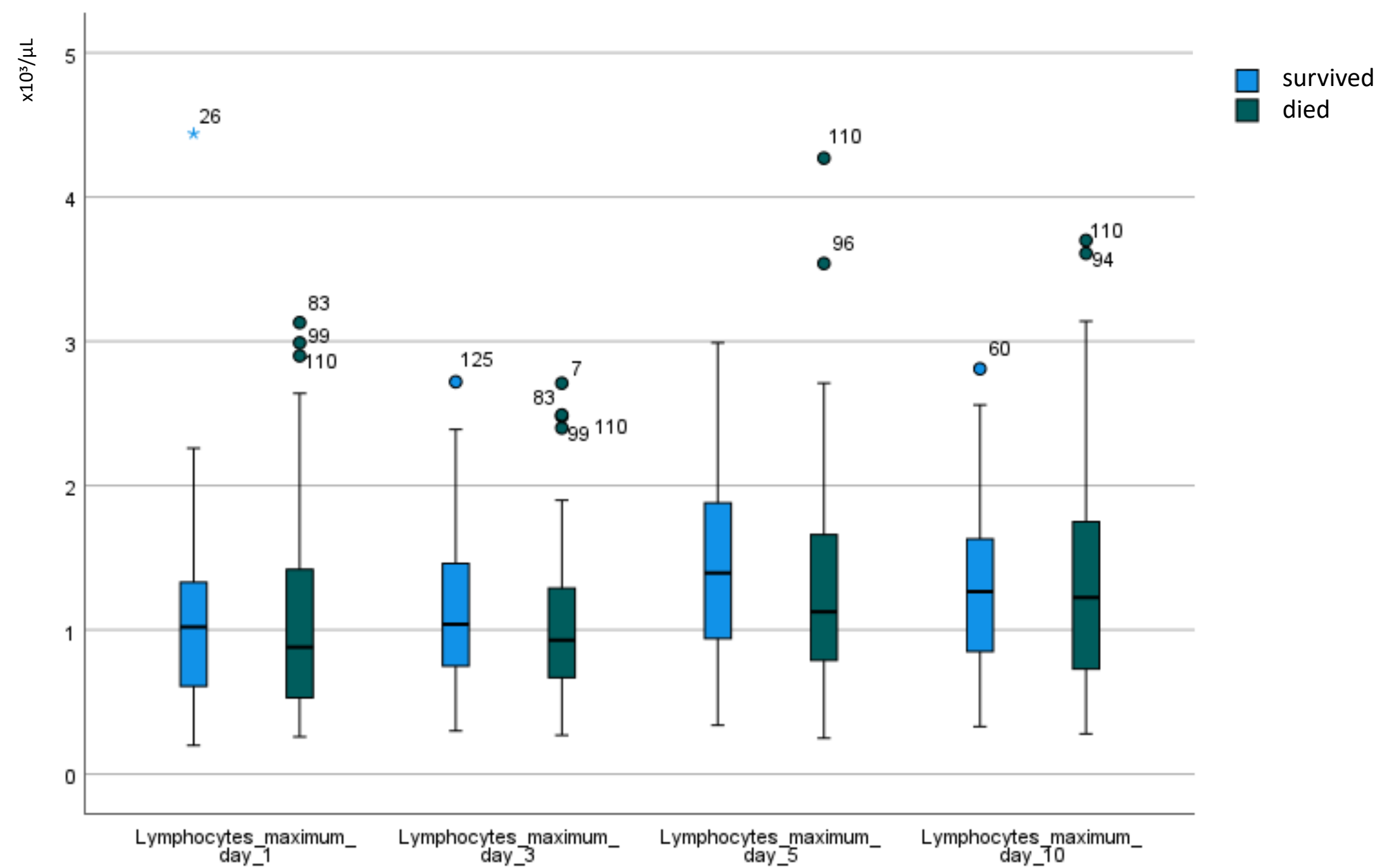

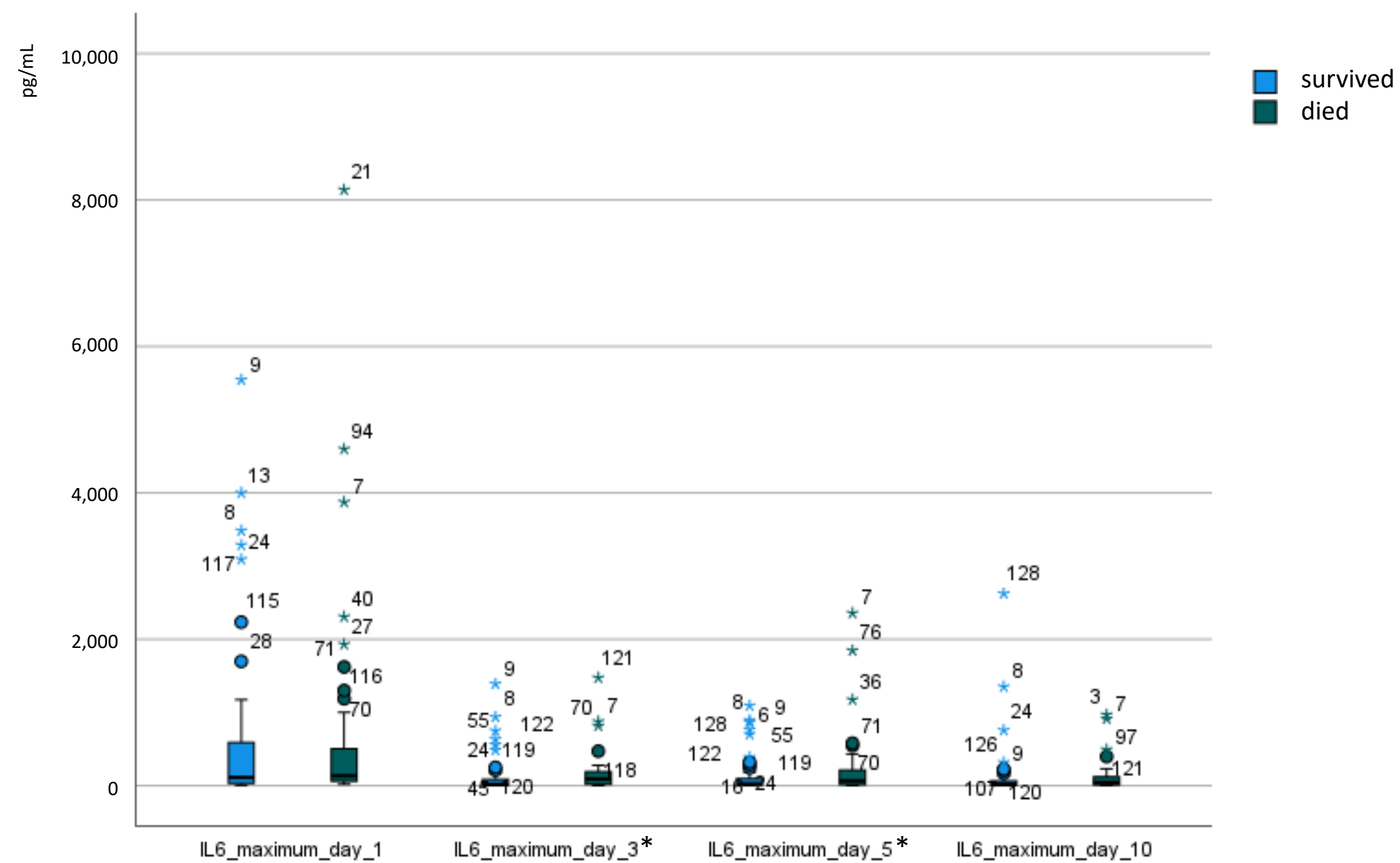

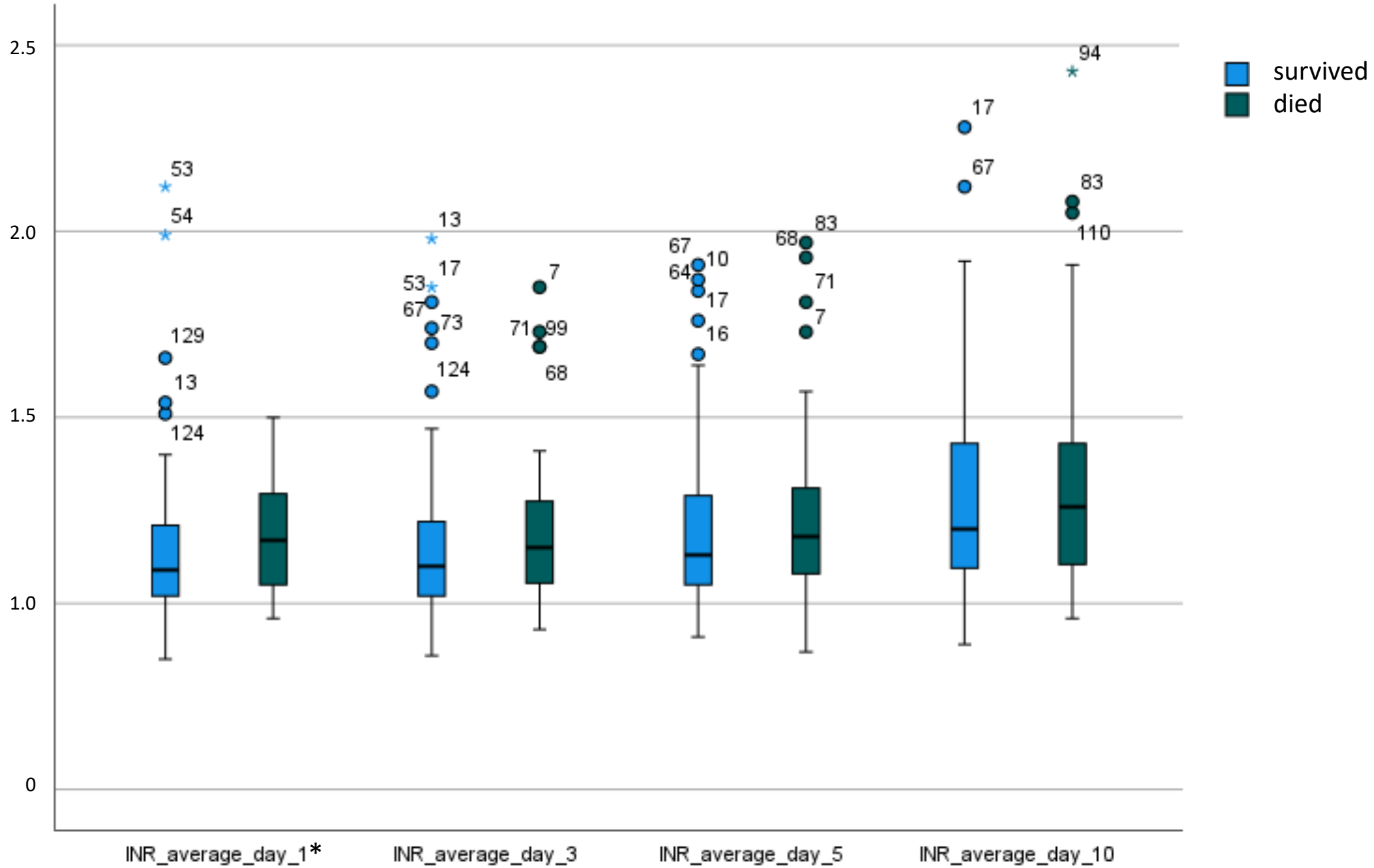

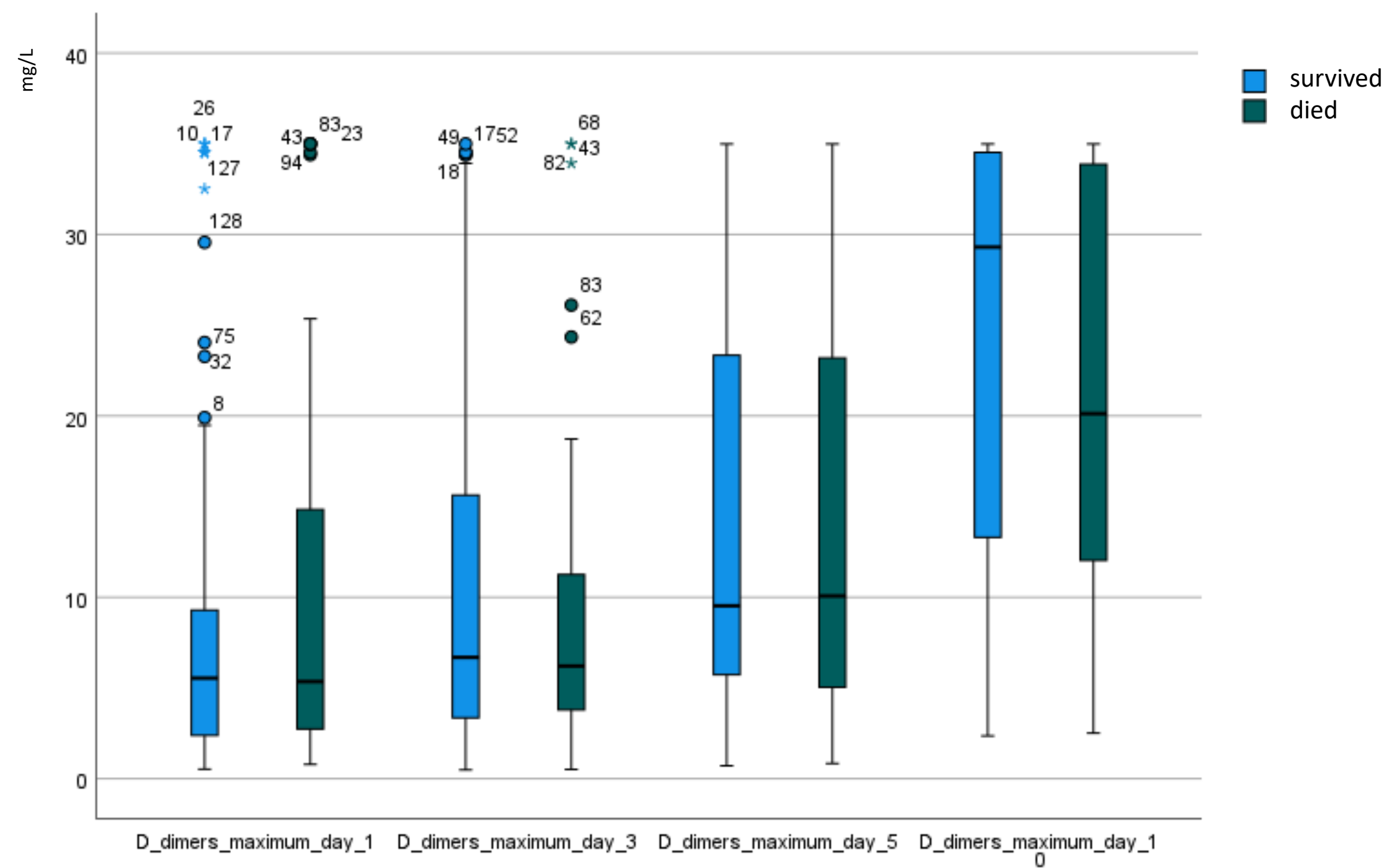

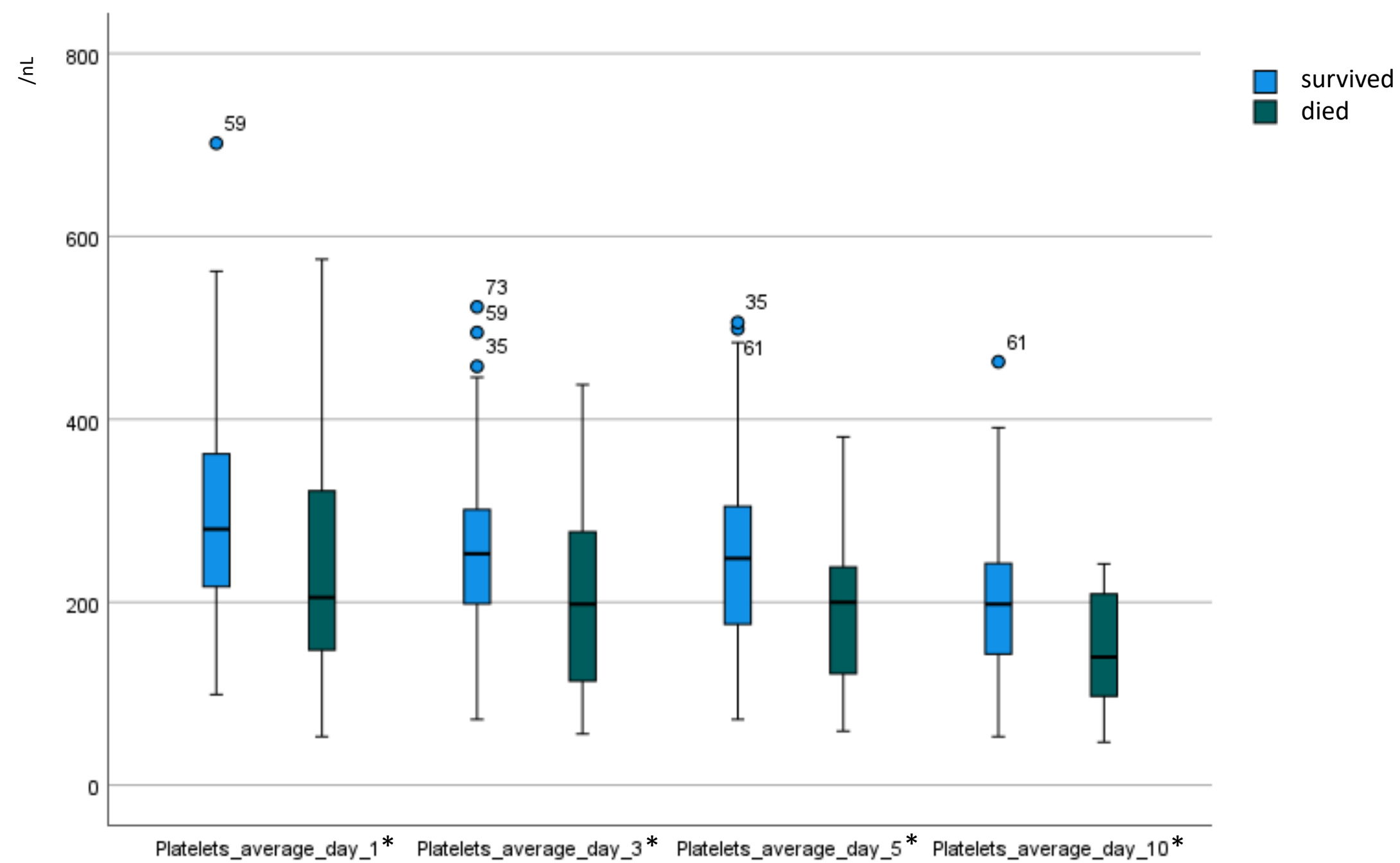

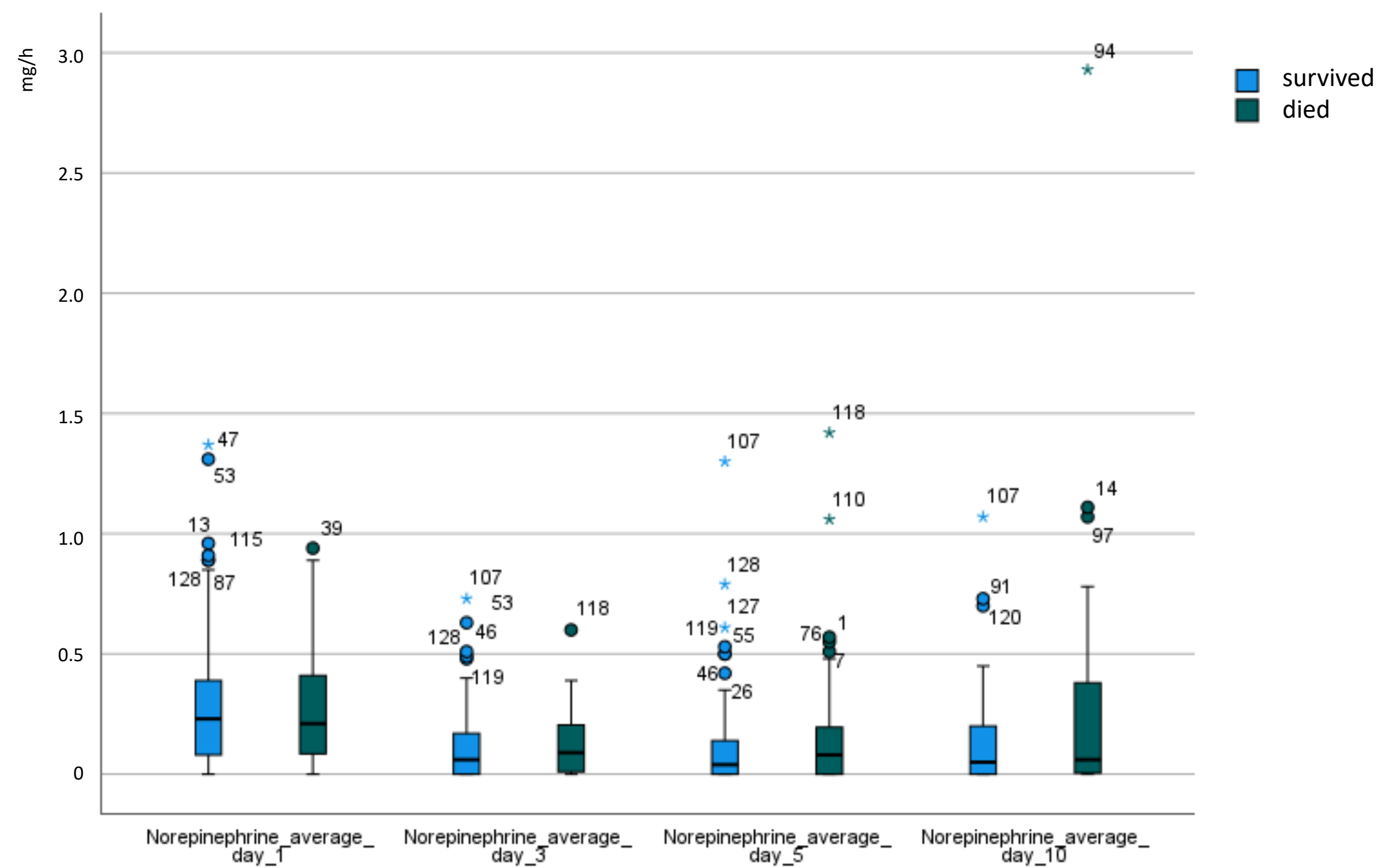

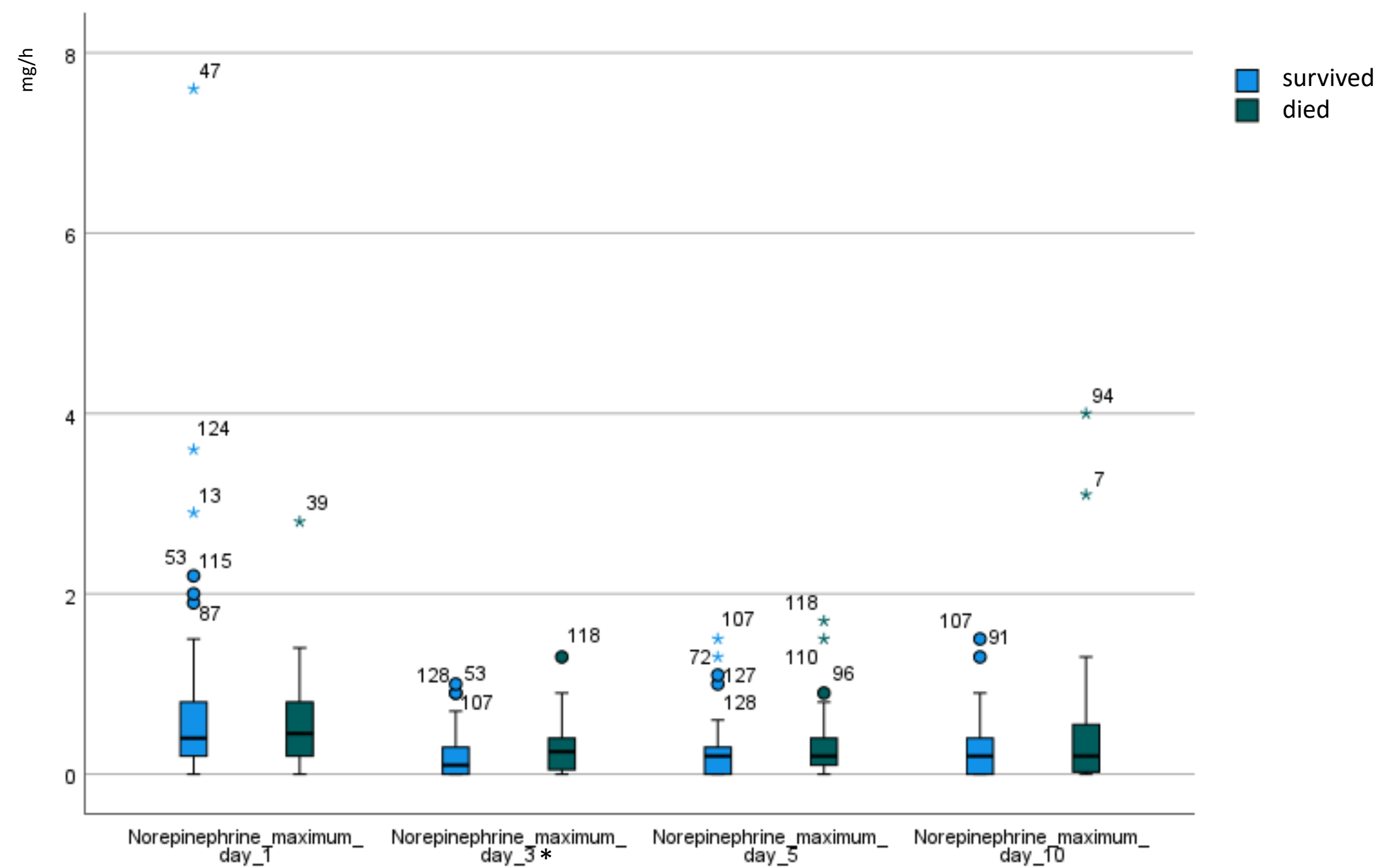

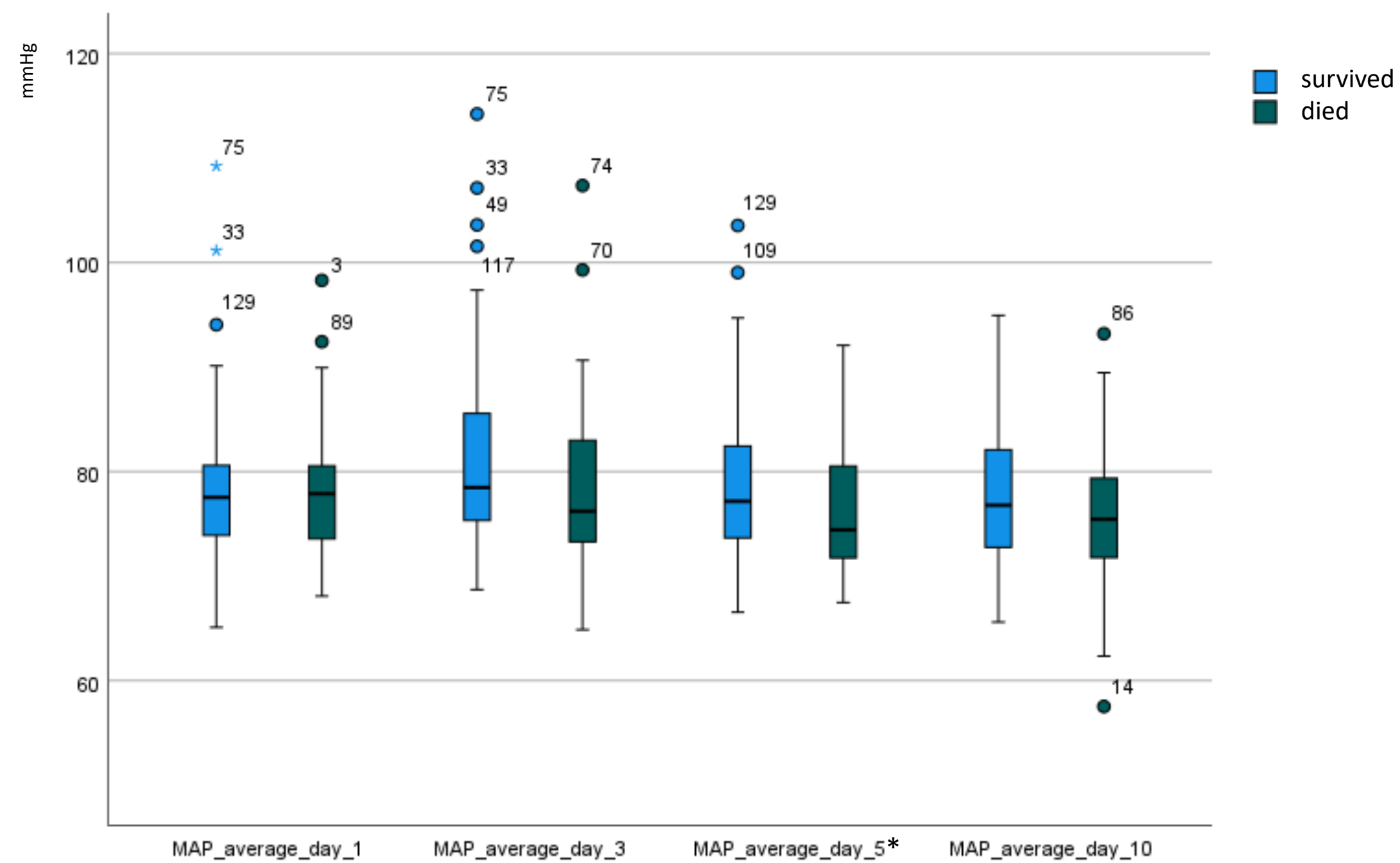

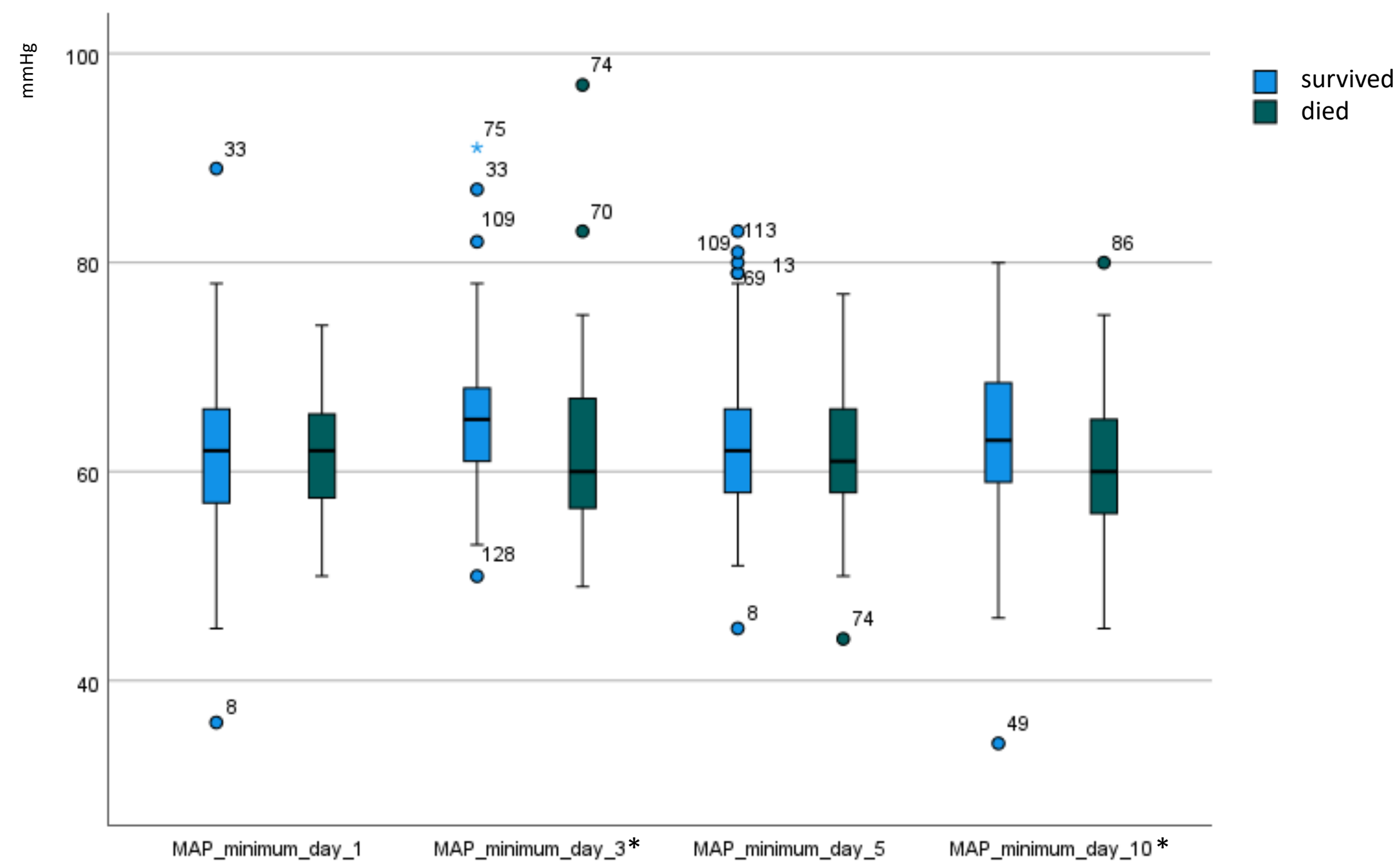

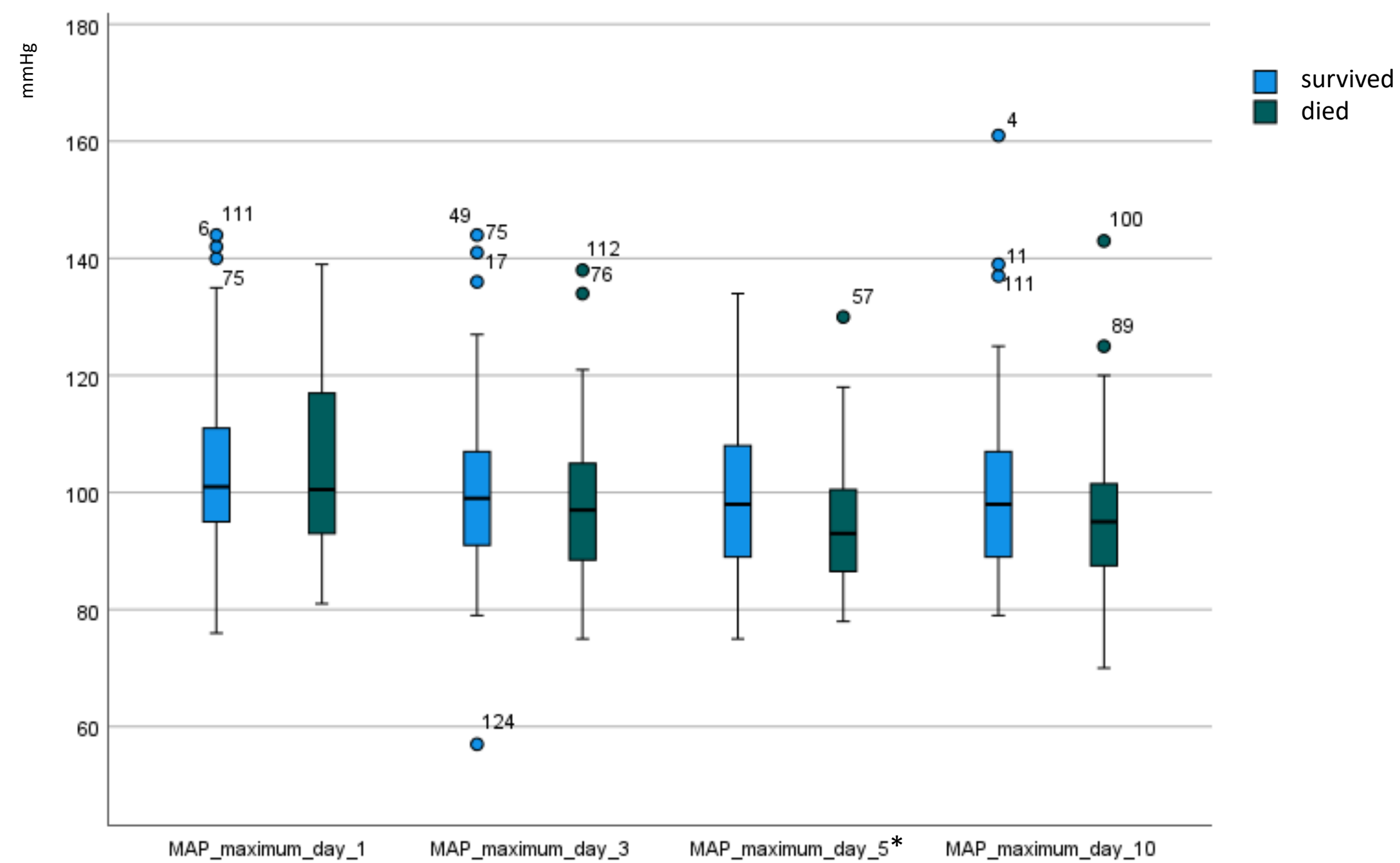

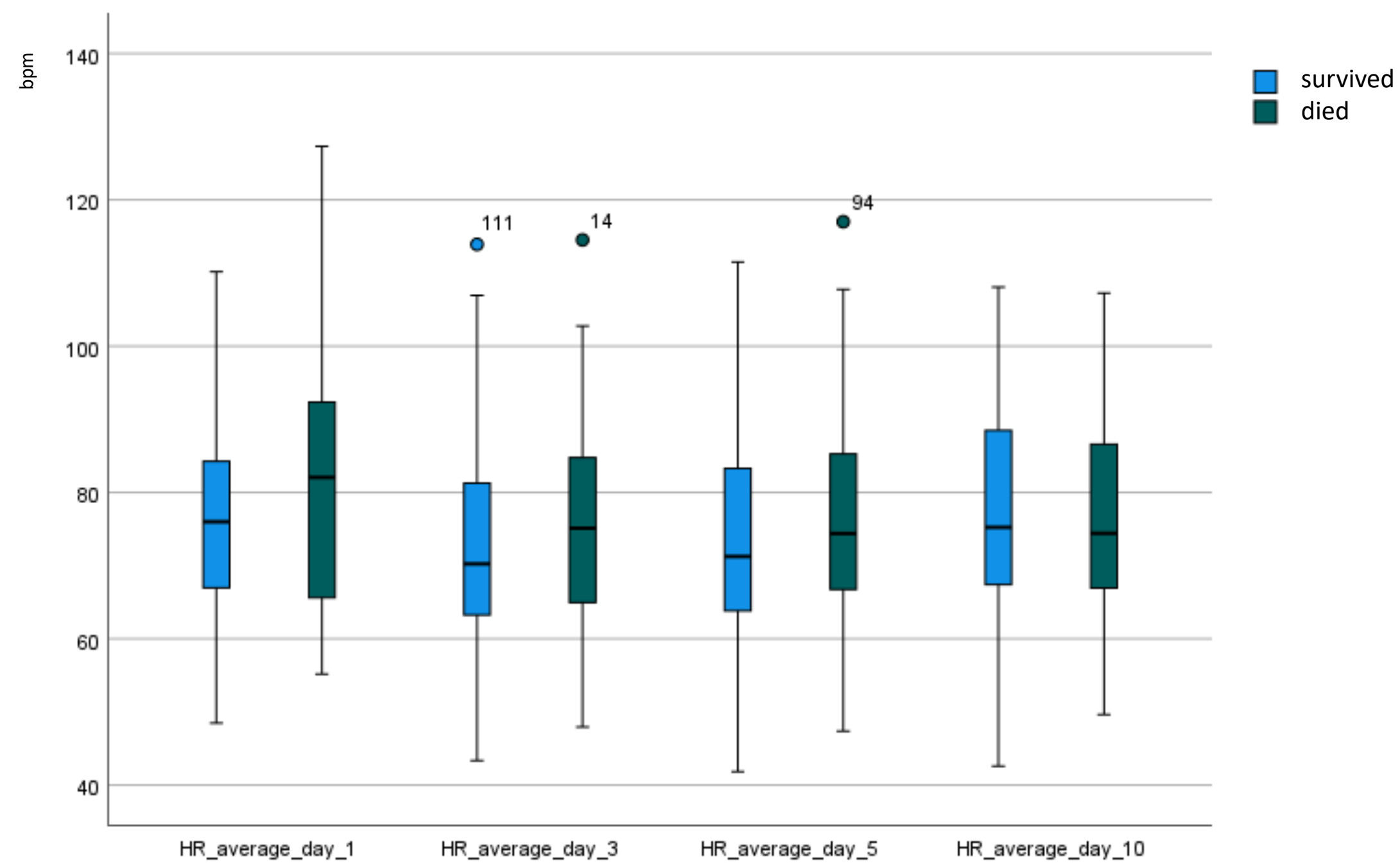

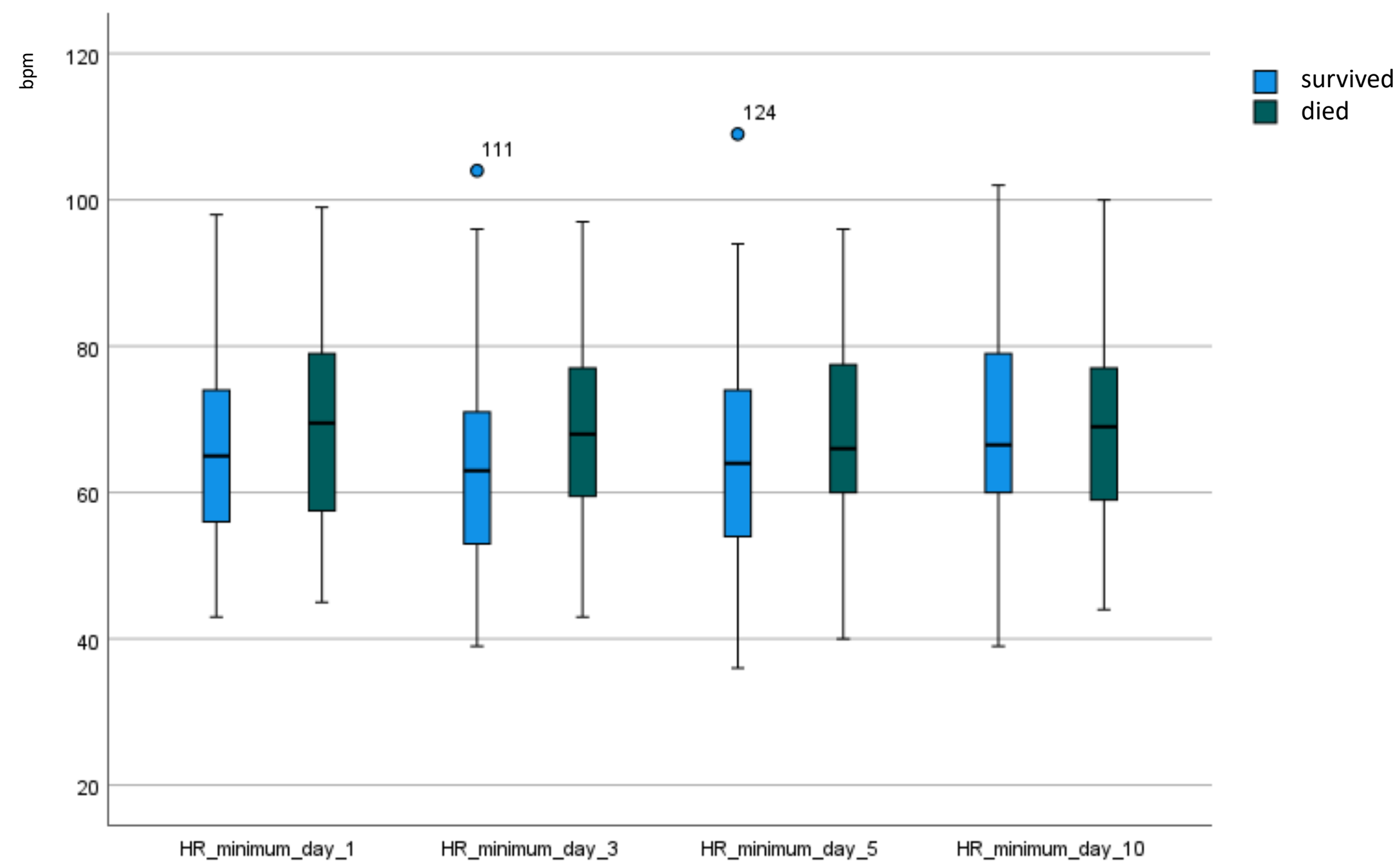

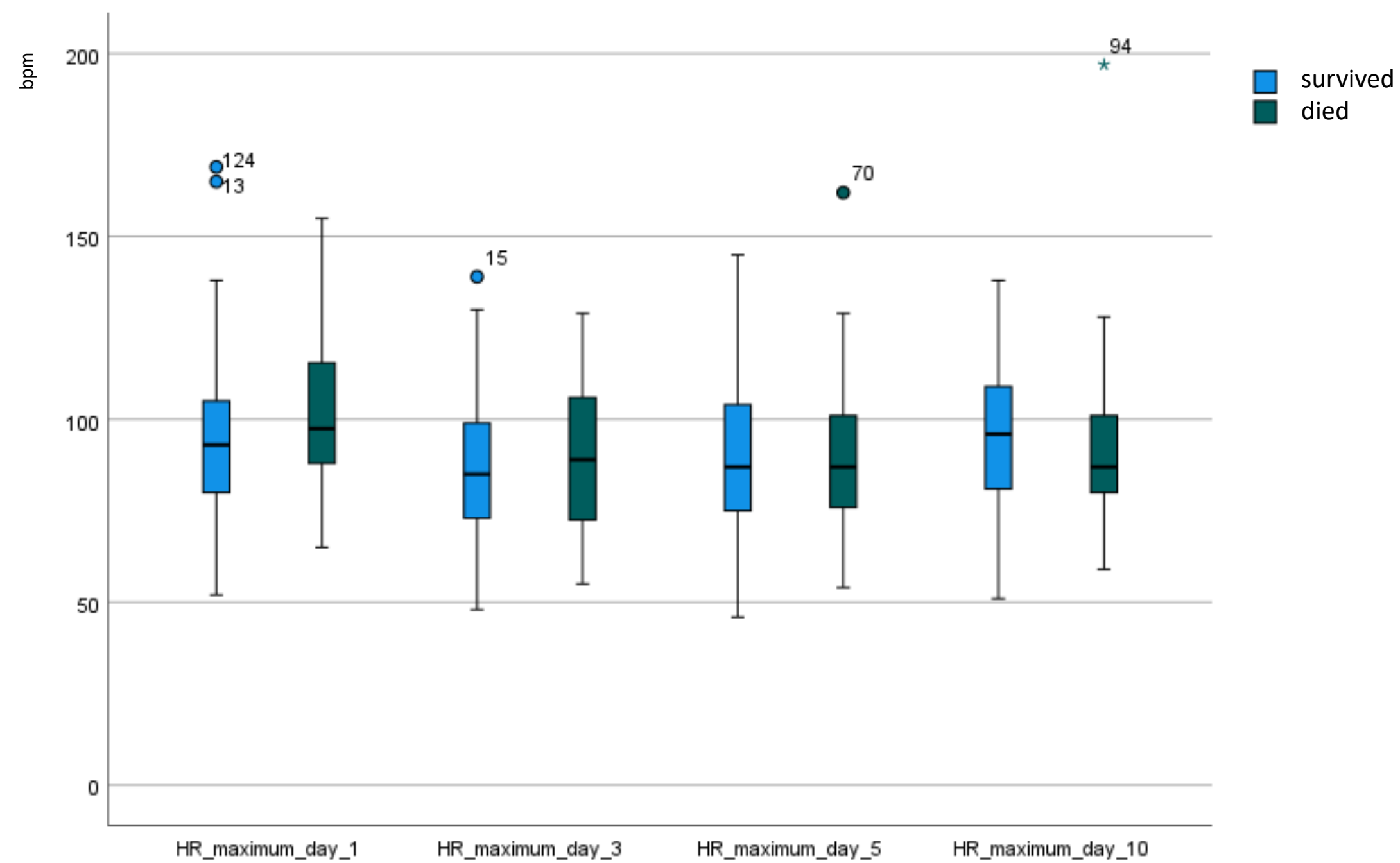

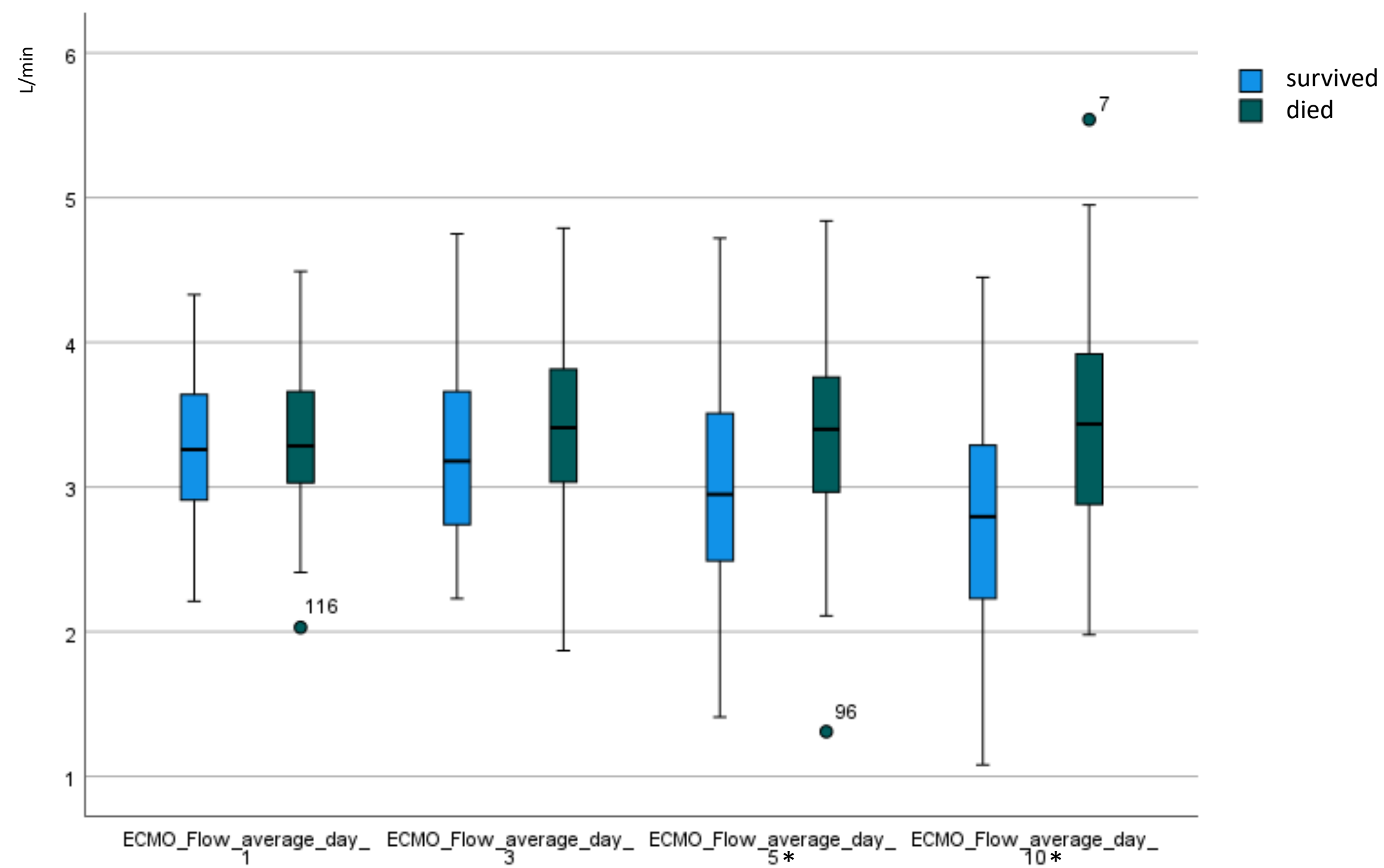

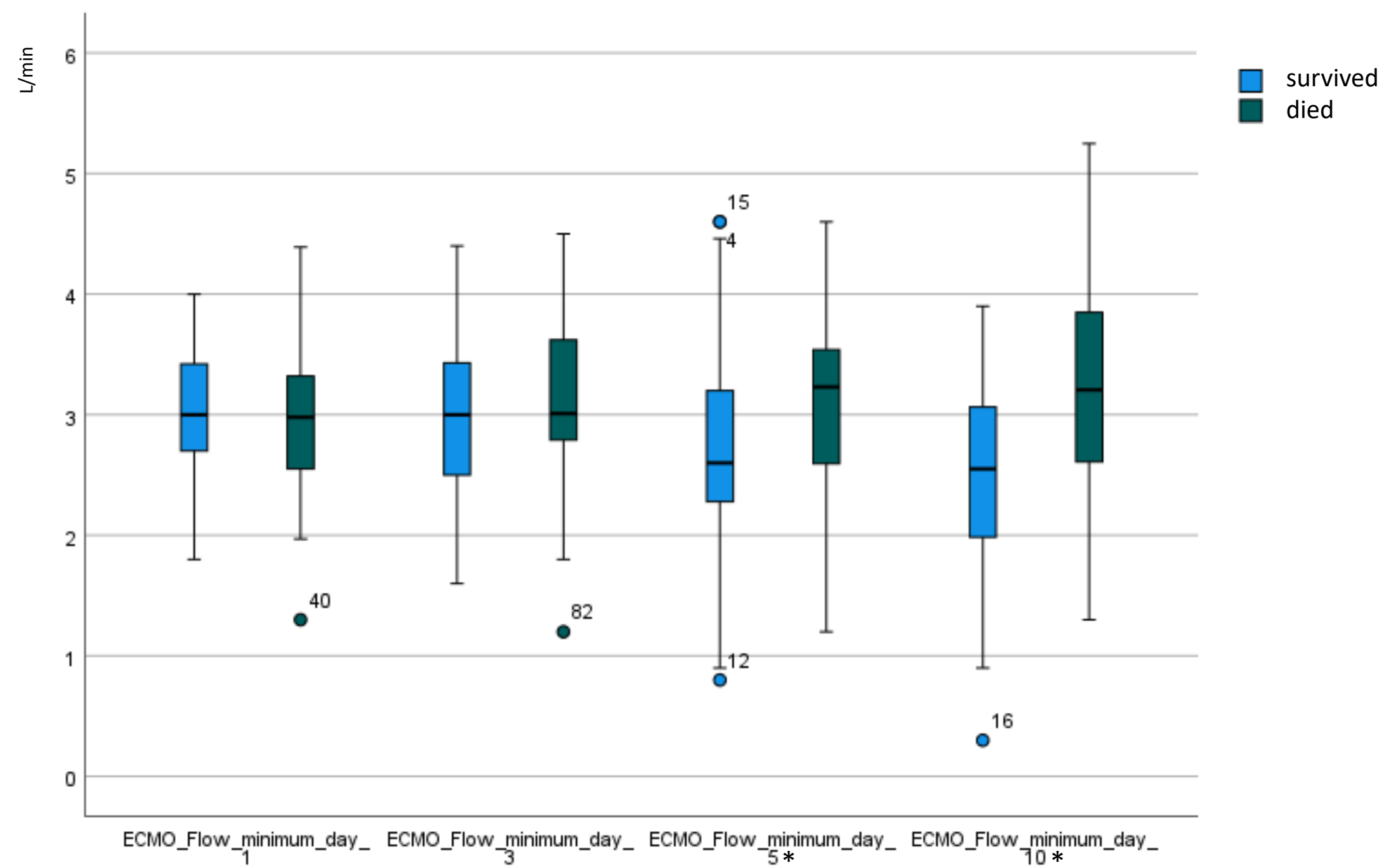

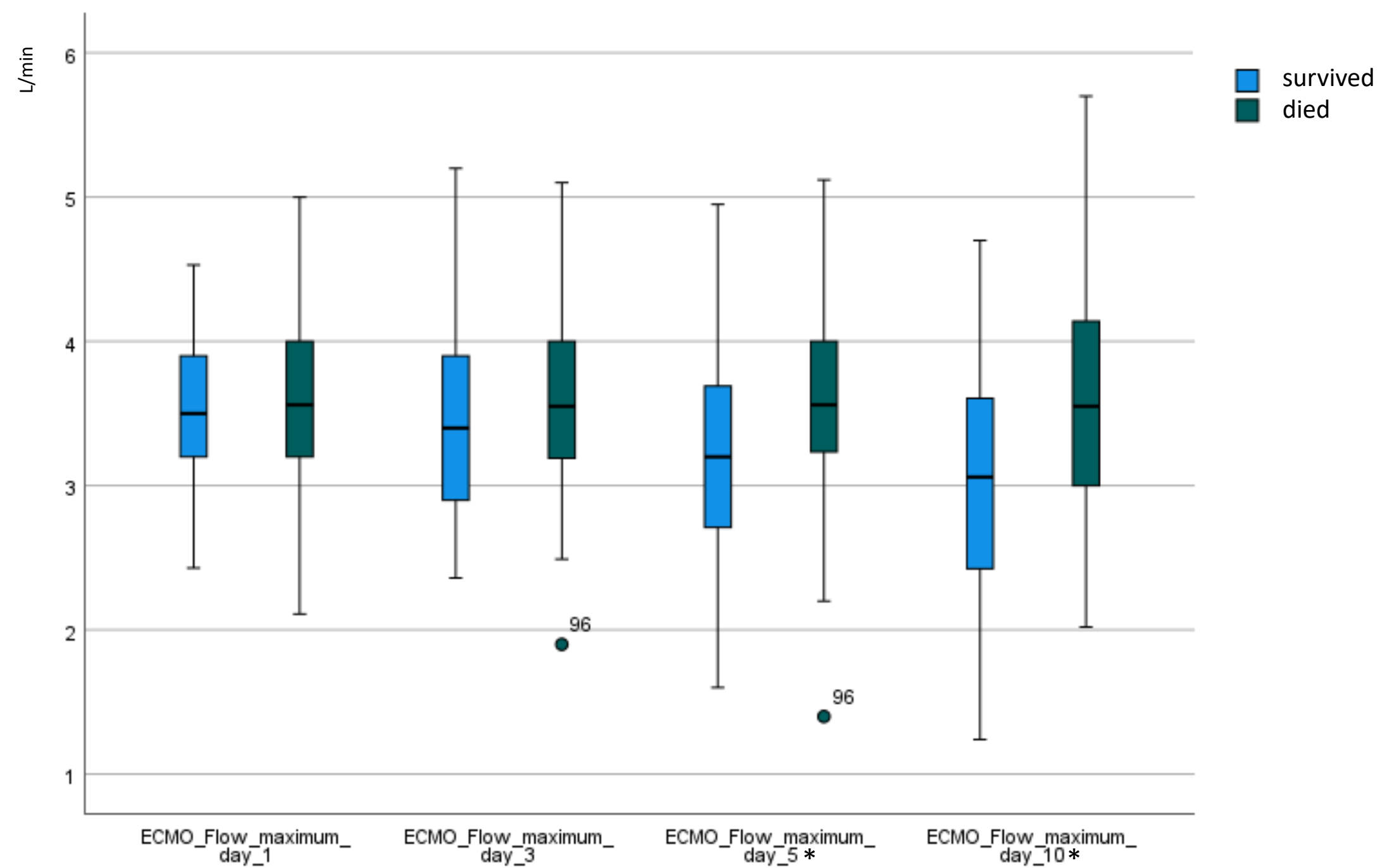

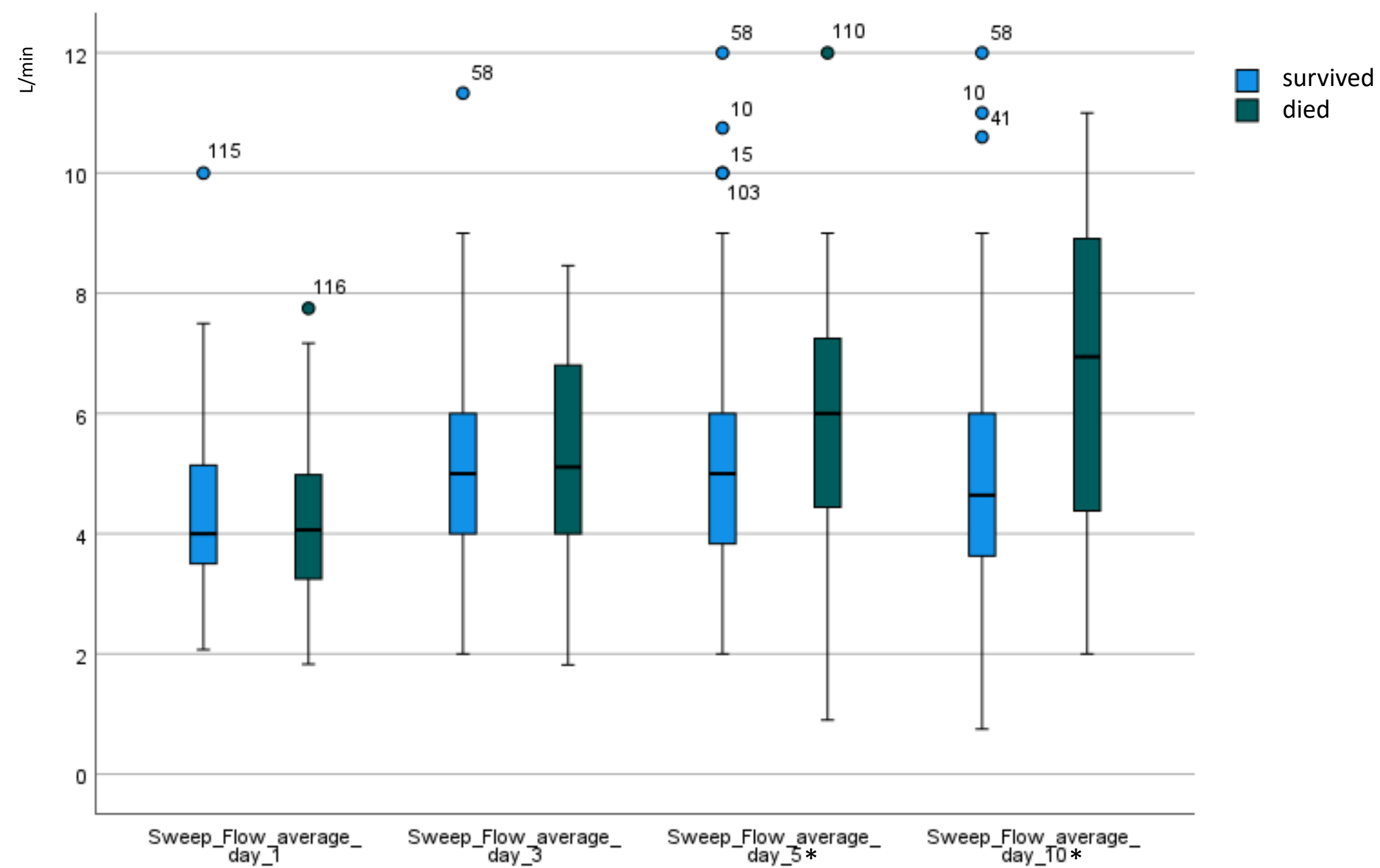

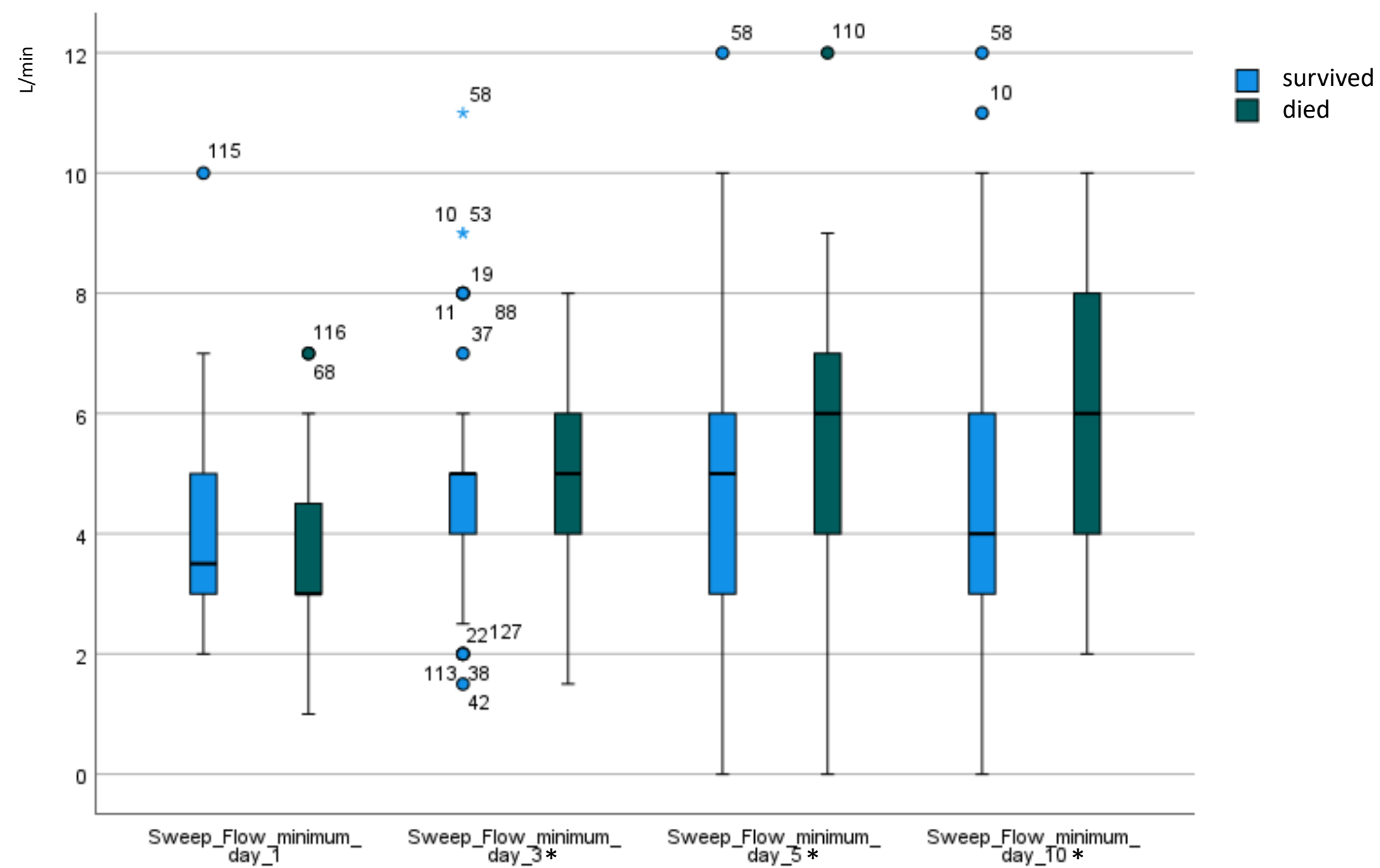

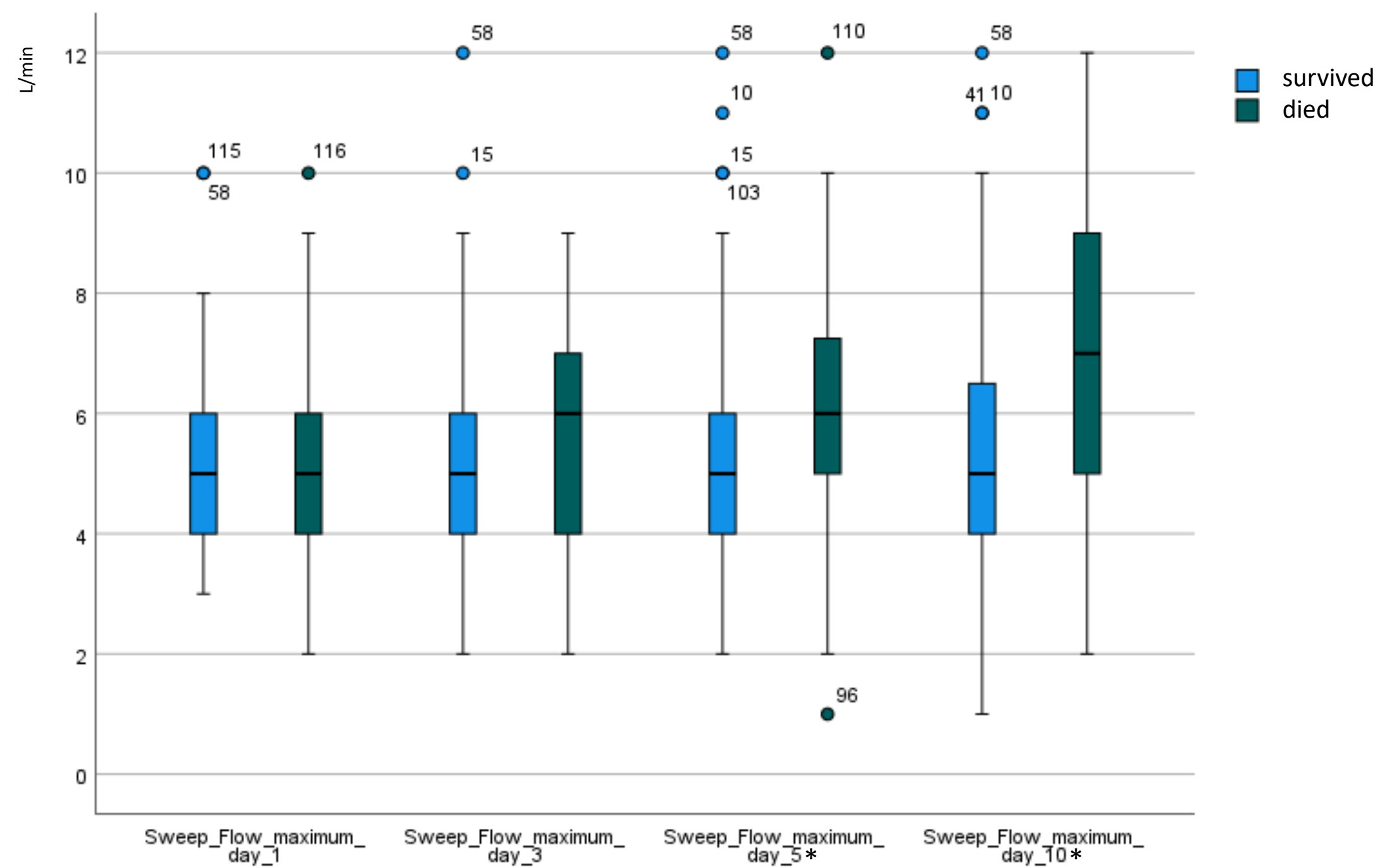

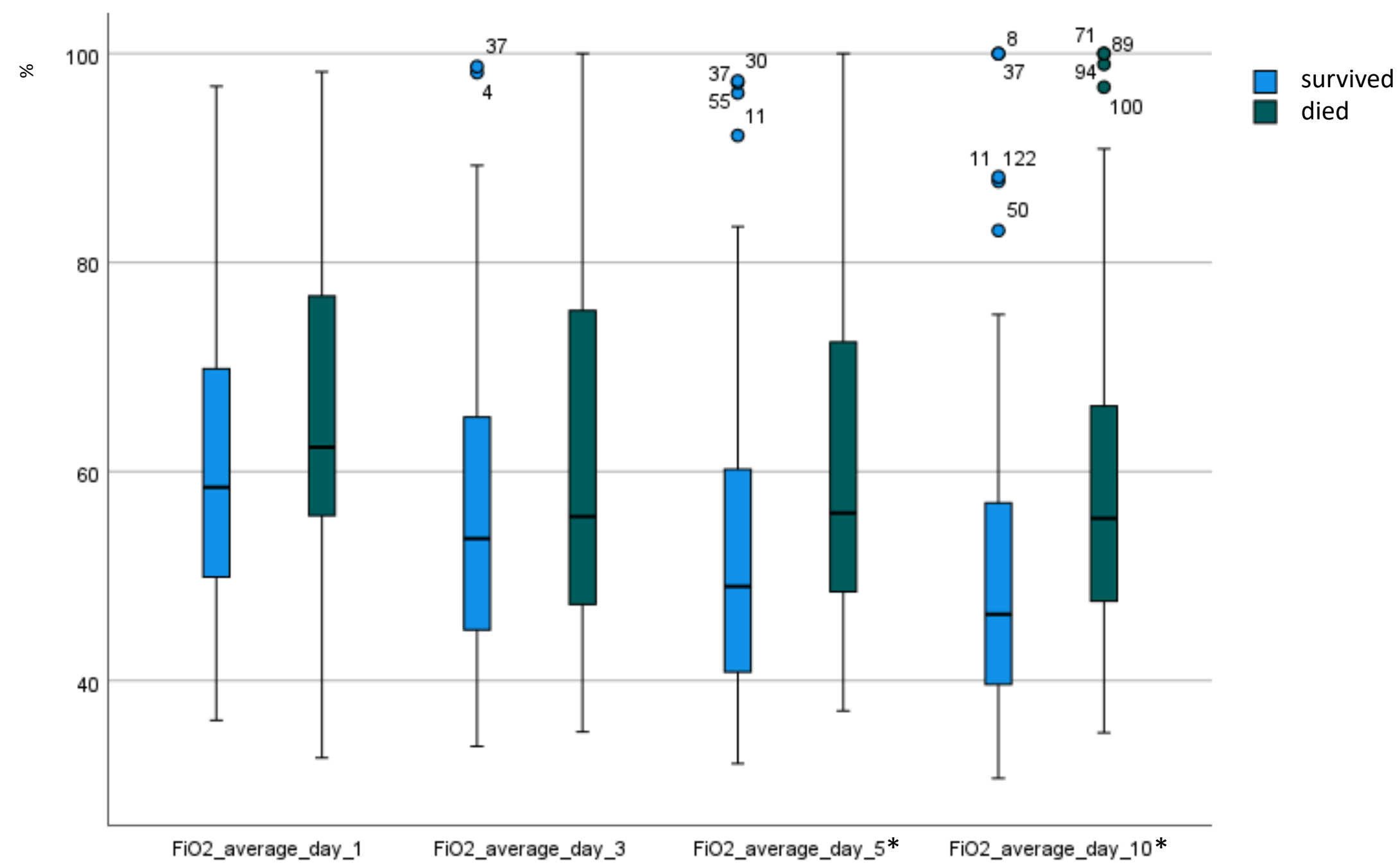

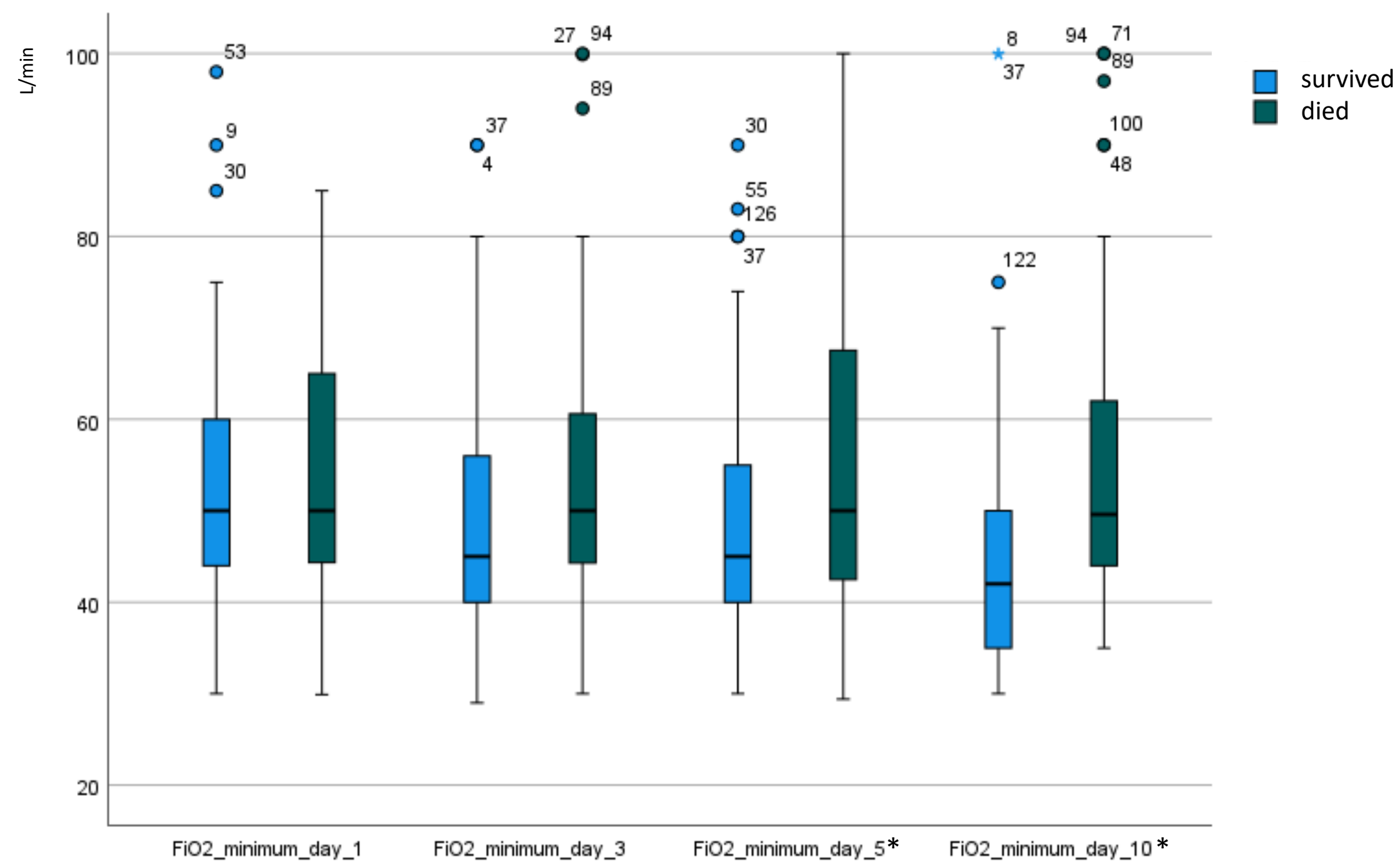

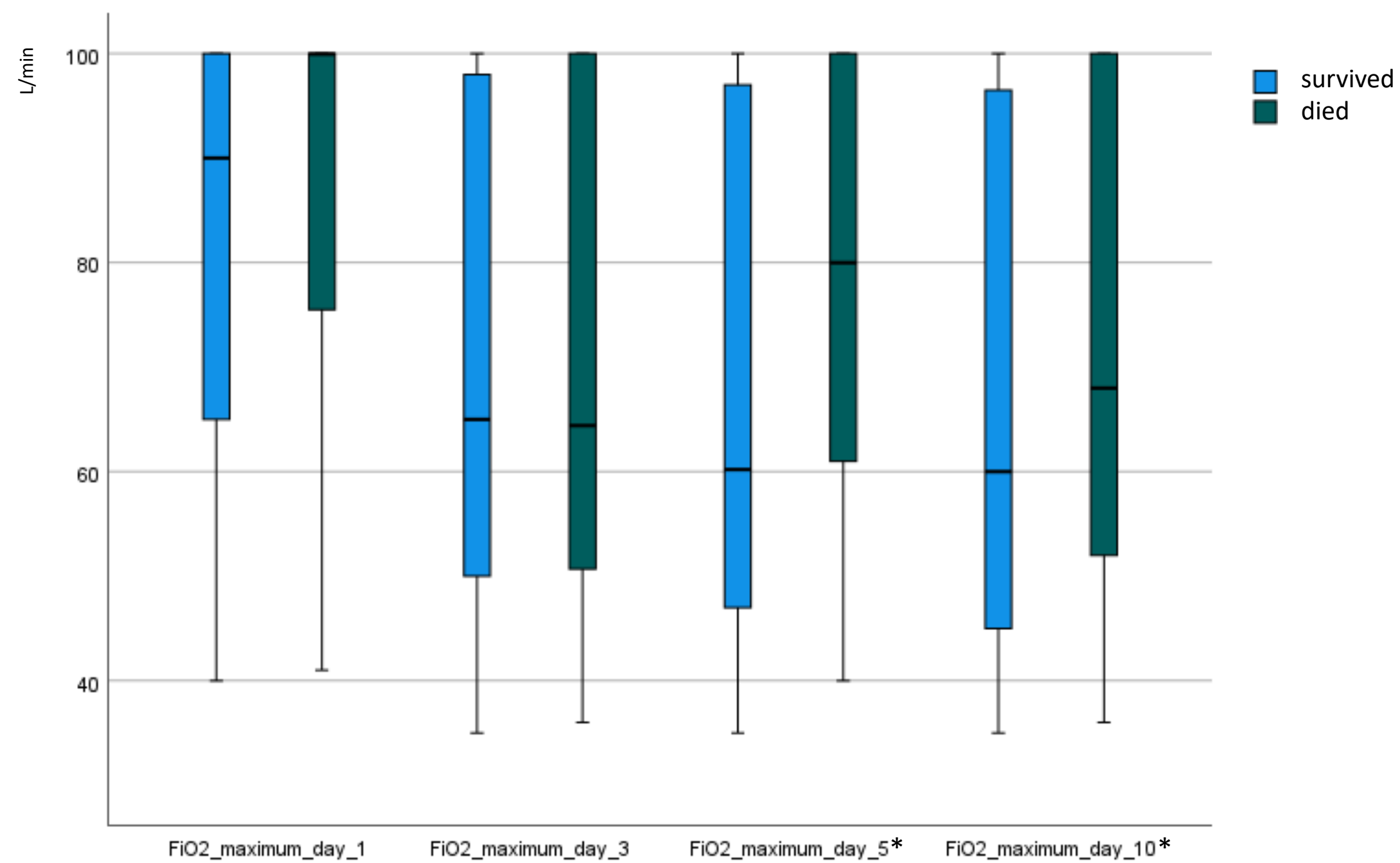

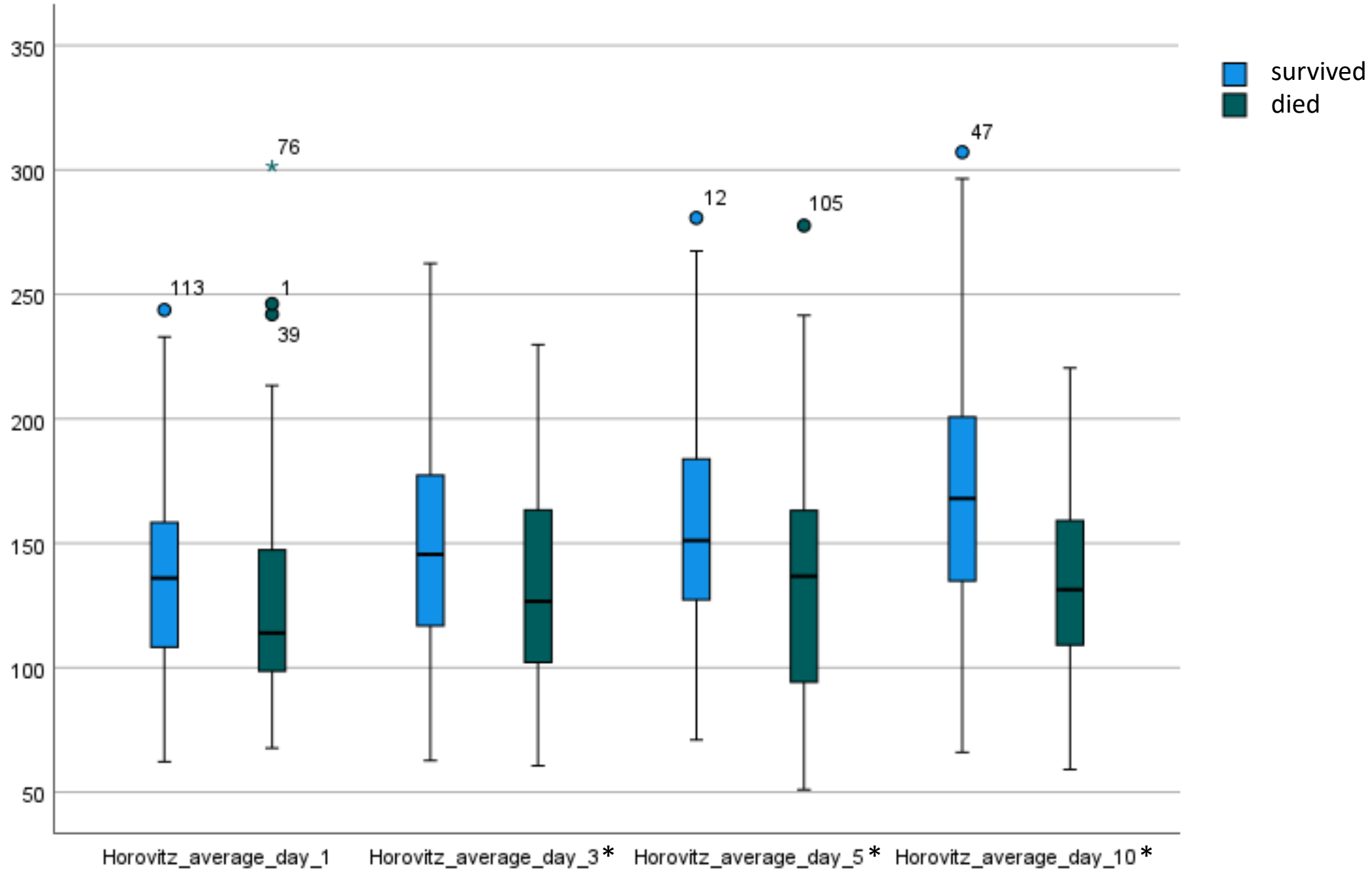

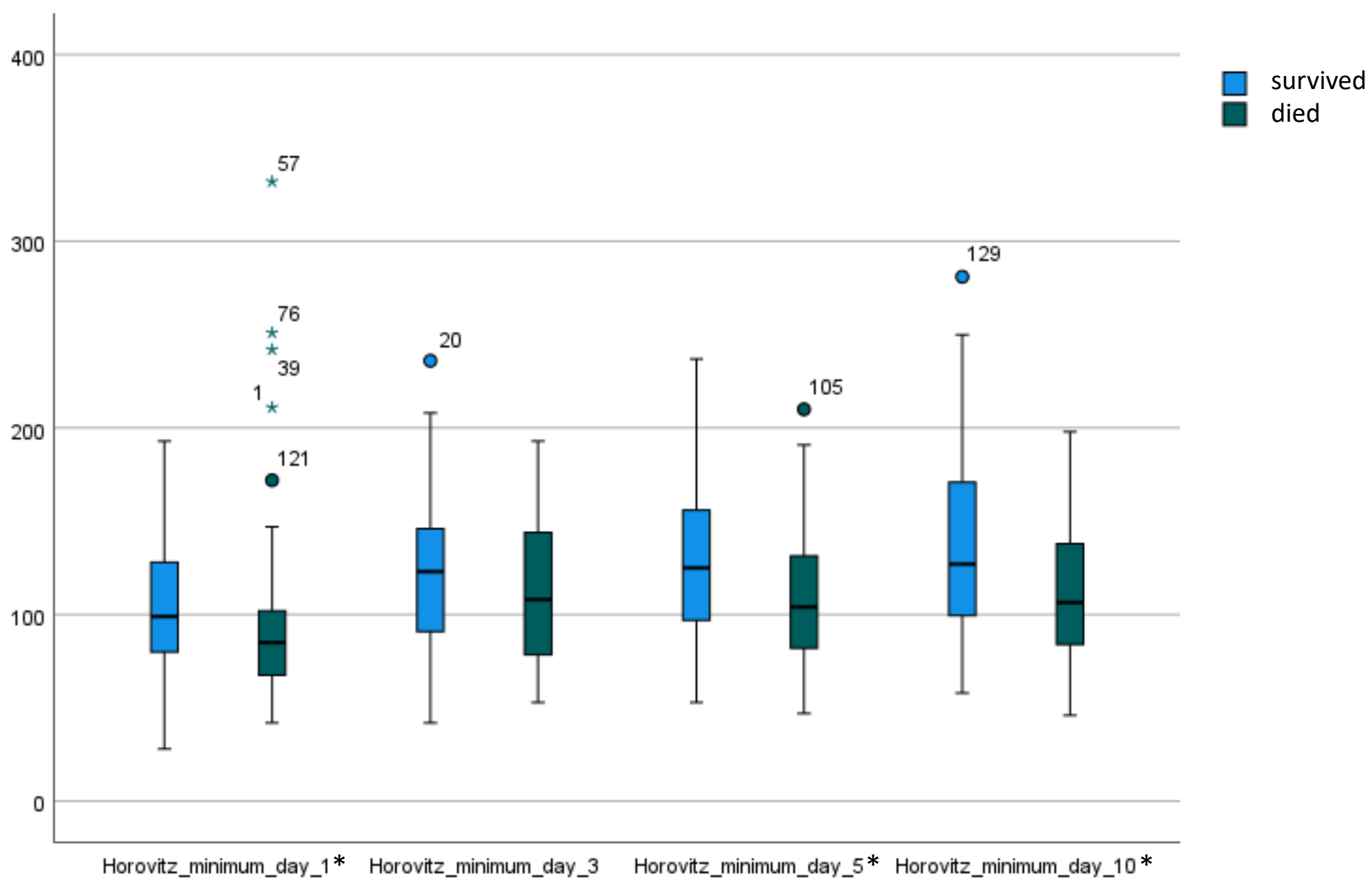

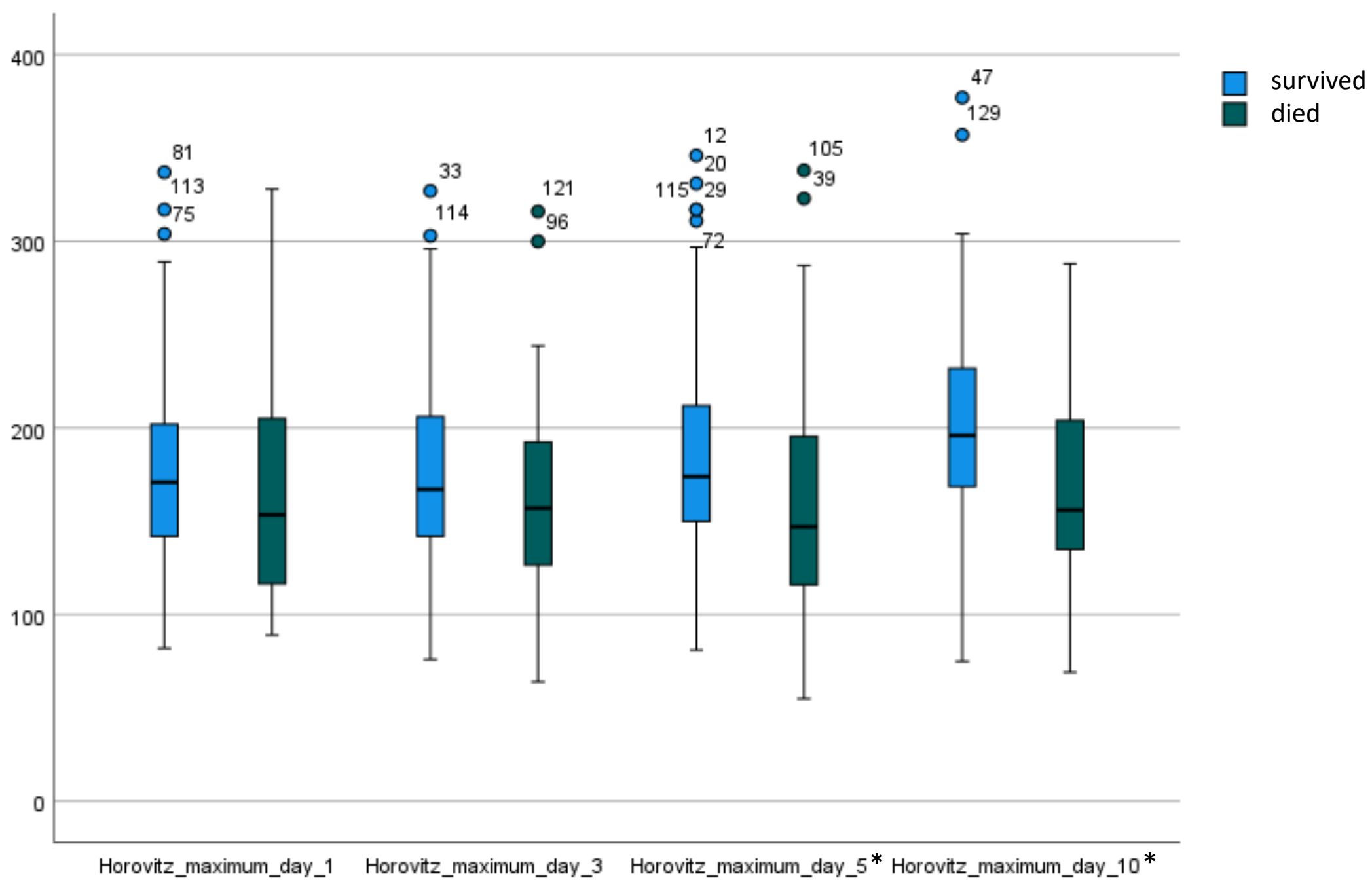

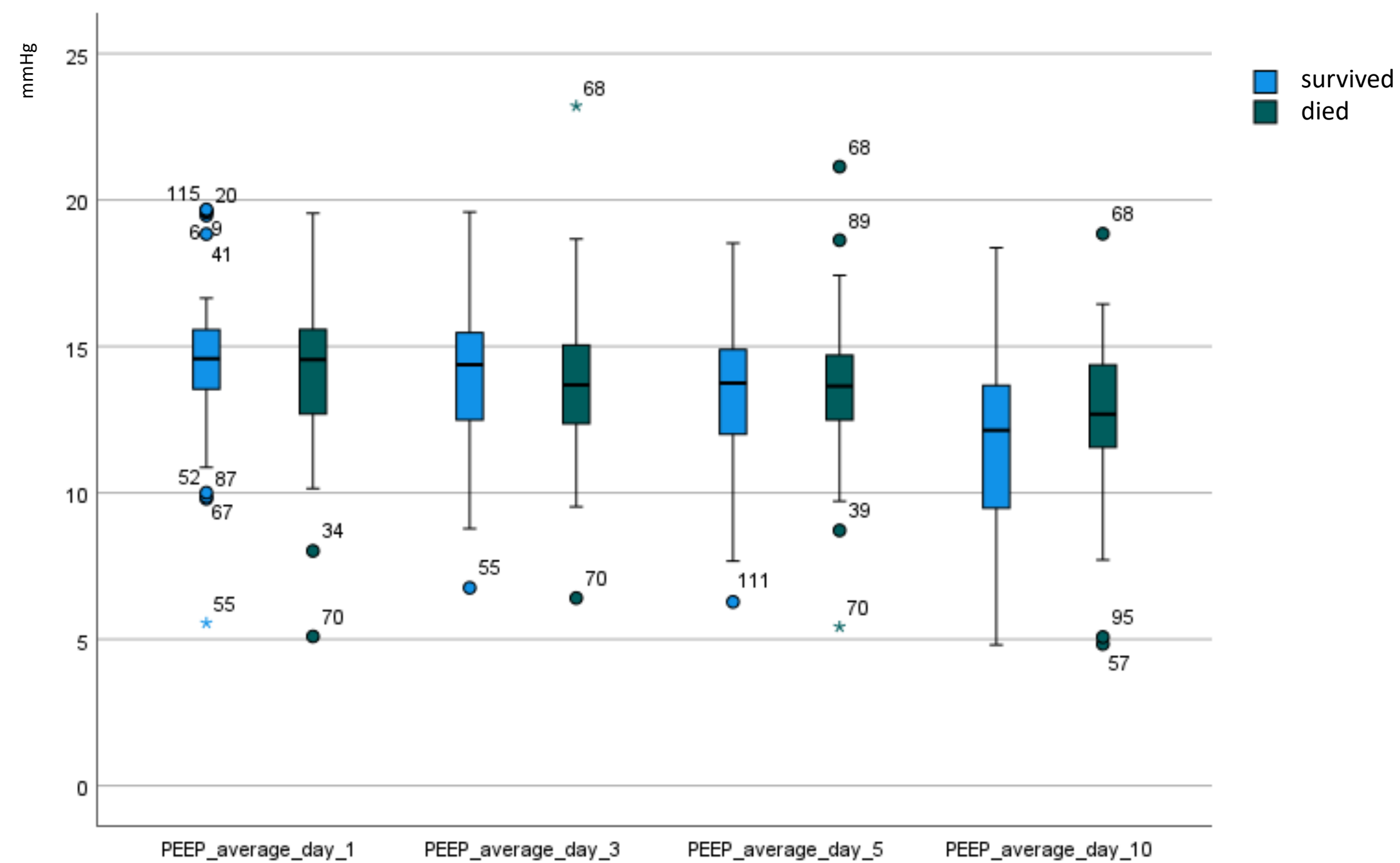

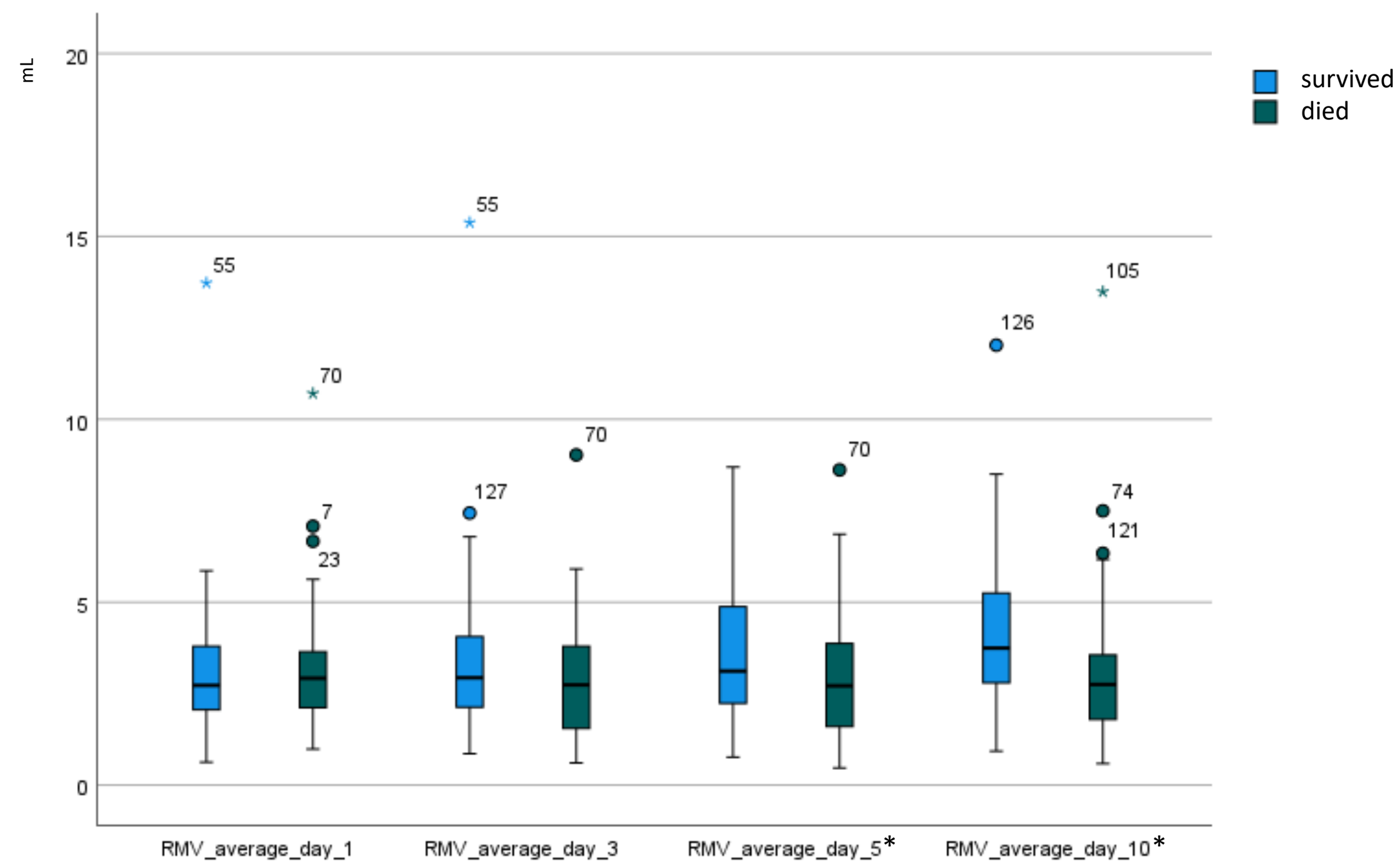

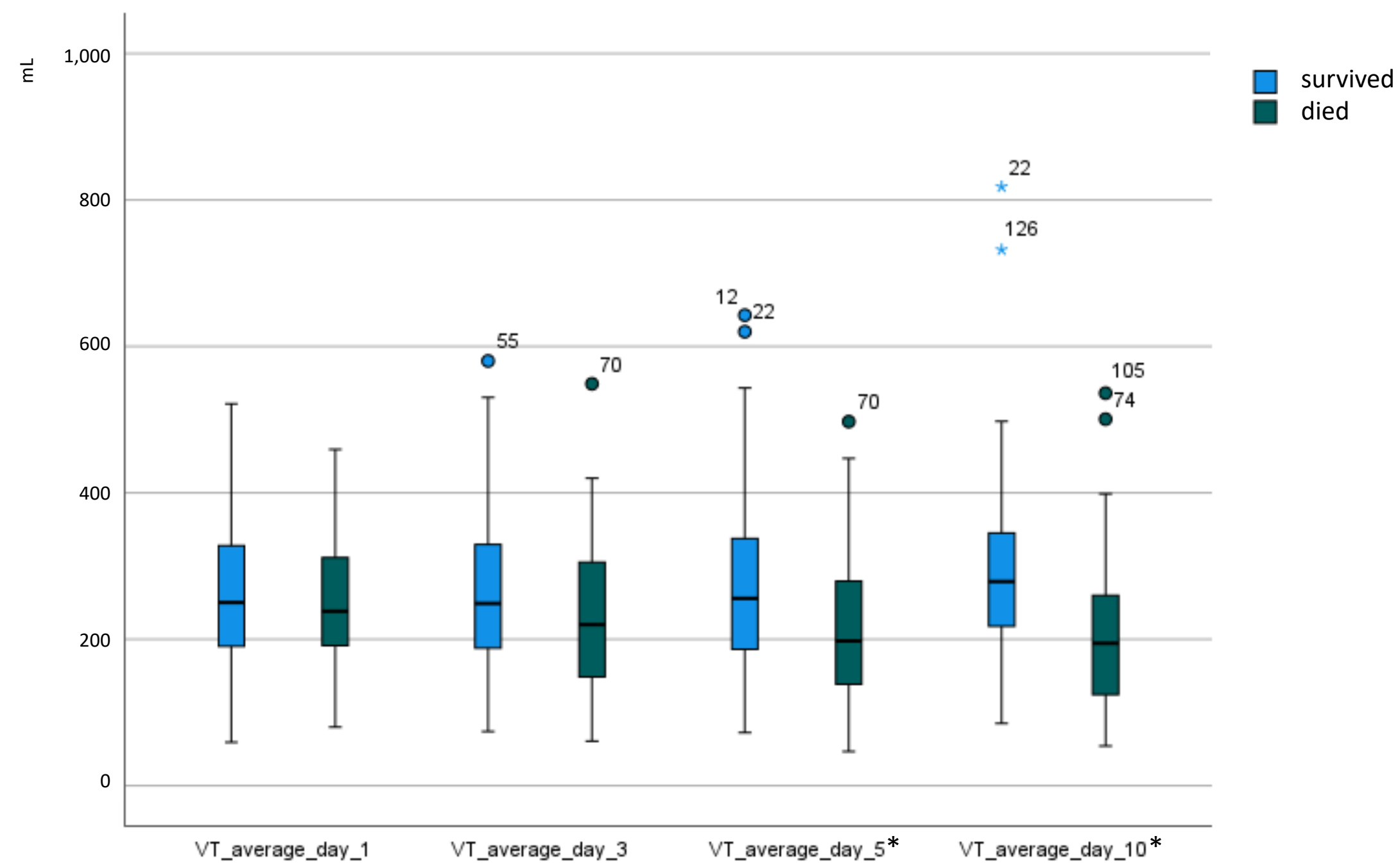

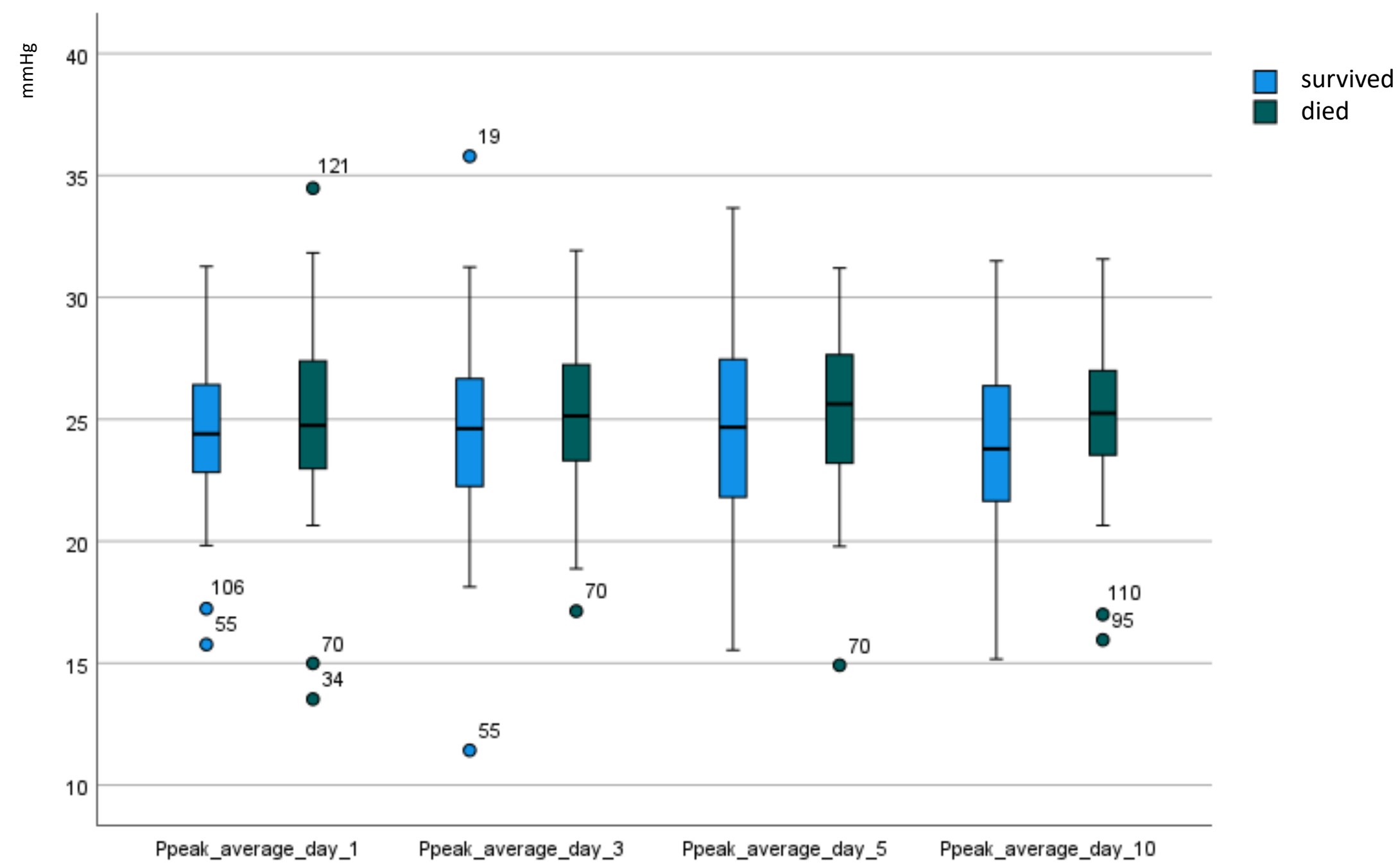

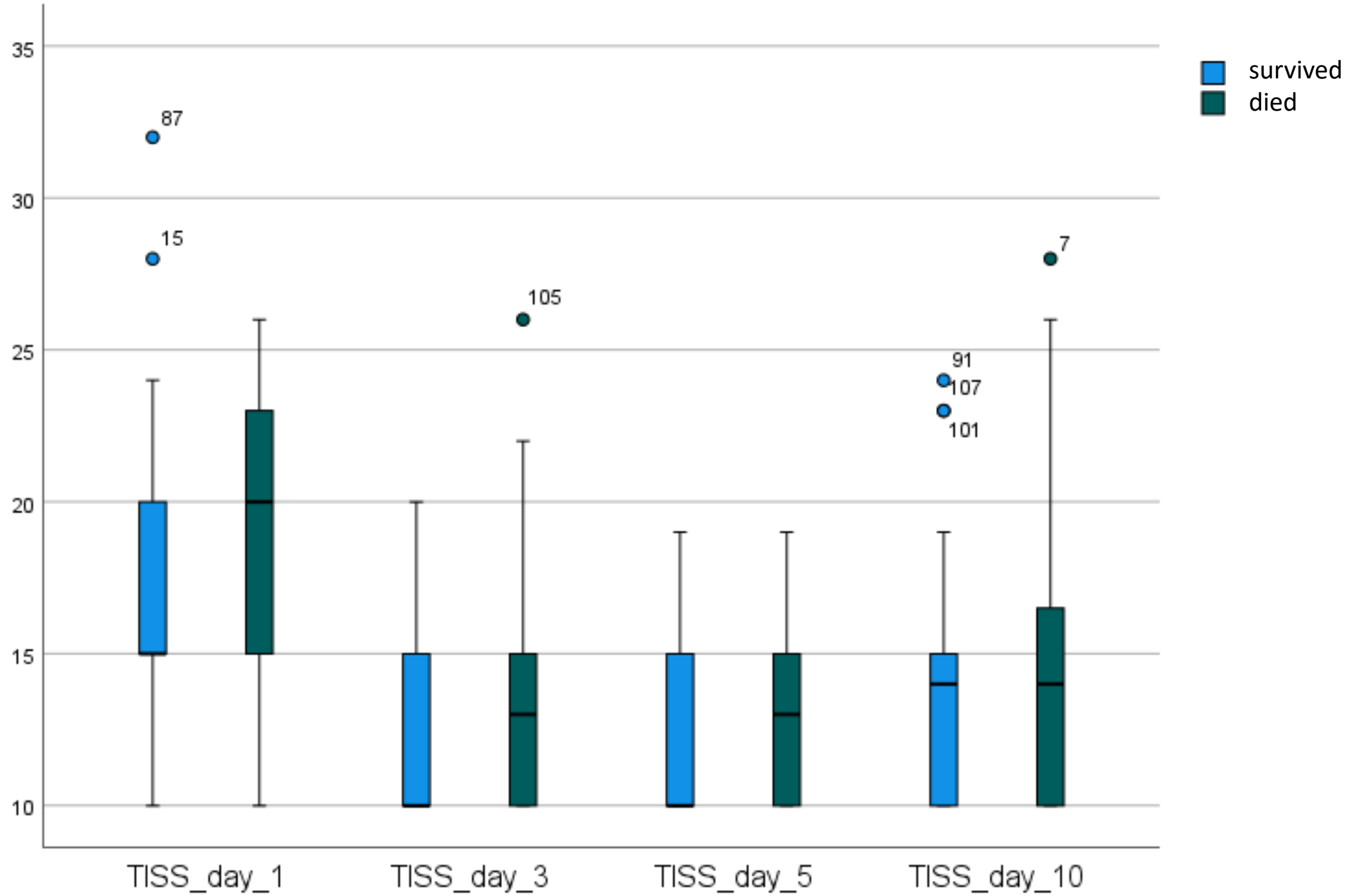

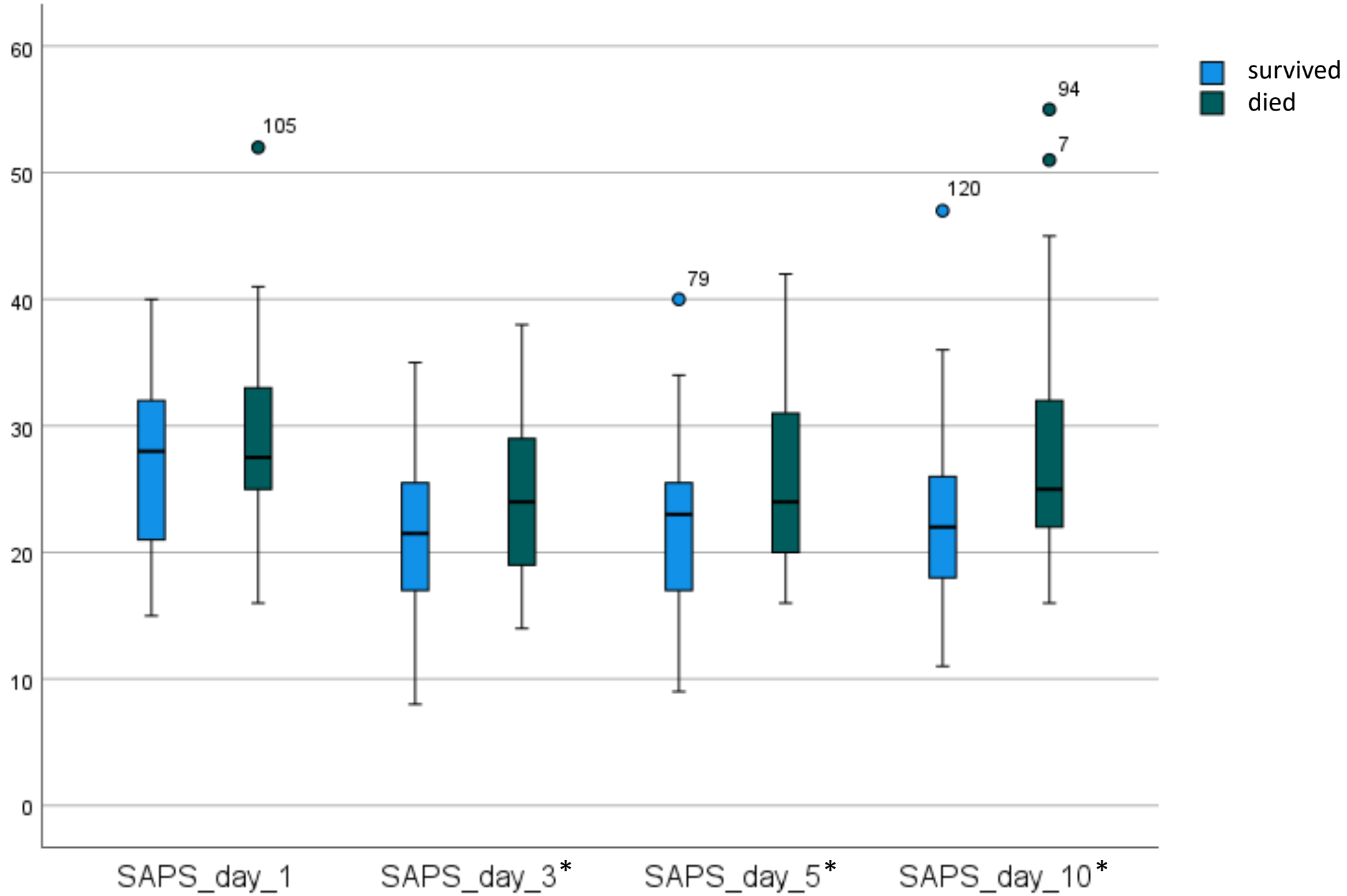

Supplement: S2 Fig — (PDF) [file pone.0280502.s007.pdf]
