# Supplementary material for: Prognostic factors for favorable outcomes after veno-venous extracorporeal membrane oxygenation in critical care patients with COVID-19
Source: PLoS One. 2023 Jan 20;18(1):e0280502. doi: 10.1371/journal.pone.0280502 (PMC9858373; doi:10.1371/journal.pone.0280502)
Supplement: Attachment — Submitted filename: Rebuttal letter 2022-12-29.docx [file pone.0280502.s008.docx]

PLOS ONE

Lakshmi Kannan

Academic Editor

Regensburg, 29.12.2022

**PONE-D-22-22958**

Revised version of the manuscript **Prognostic factors for favorable outcomes after veno-venous extracorporeal membrane oxygenation in critical care patients with COVID-19**

Dear Lakshmi Kannan,

On behalf of my co-authors, I would like to thank you and the reviewers for your valuable comments and suggestions to improve the quality of our manuscript. We have revised the manuscript according to the reviewers' comments. Enclosed you find a point-by-point response to each comment indicating the action taken or the revision made.

In addition to this letter, we have uploaded a version of the revised manuscript with tracked changes ('Revised Manuscript with Track Changes') and a version without tracked changes ('Manuscript').

Thank you very much again for your help in improving the quality of our paper. We trust that our manuscript is now suitable for publication in PLOS ONE.

Sincerely,

PD Dr. Martin Kieninger

Please take note of the following statement regarding Martin Kieninger's service as Academic Editor for PLOS One: This does not alter our adherence to PLOS ONE policies on sharing data and materials.

**Reviewer #1:**

**1. Was the cutoff for ECMO initiation at 14 d of mech vent due to your center's criteria used for all patients or just COVID?**

At our center, we do not have a fixed cutoff until when VV ECMO therapy is started after initiation of ventilator therapy. In justified cases, the implantation of ECMO can still be performed a few weeks after the start of ventilation therapy as a case-by-case decision. This was also done in COVID-19 patients.

In the present study, we were interested in capturing the initial phase of COVID-19 disease with severe respiratory failure and ECMO therapy. The cutoff of 14 days was chosen because, of the 131 patients included, 129 were started on VV ECMO during this period; only 2 patients were implanted on VV ECMO later (see S1 Figure).

**2. Was there also info on survival to discharge not just ICU?**

Unfortunately, we have data on this for only a small fraction of patients, as most patients were transferred from the ICU to either a rehabilitation facility or a non-tertiary hospital.

**While the association of plt count and outcome is interesting, one wonders if this is clinically significant. There is also no data on how many plt transfusion were required or given between groups, the number of circuit changes etc, all of which can affect plat ct.**

The aim of our study was to investigate a variety of parameters relevant to intensive care medicine with regard to their suitability as prognostic markers. Platelet count was identified as a very important prognostic parameter in COVID-19 patients with VV ECMO therapy. However, due to the design of our study, no further information on the cause of this and the actual clinical relevance can be derived. As a next step, the identified prognostically relevant parameters would now have to be further investigated in a targeted manner.

However, we have attached another file as a supplement in which technical data on ECMO therapy can be found (S3 Appendix). It also lists the number of platelet concentrate transfusions during the entire ICU stay and the number of membrane oxygenators required during the entire period of ECMO therapy for each patient.

The number of patients who required transfusion of at least one platelet concentrate during the ICU stay did not differ between survivors and non-survivors (23.4% vs. 22.9%).

**3. It seems that the NS had more signs of infection and being sicker overall at day 10--higher flows, need for higher Fio2, higher PCt, higher lactate.**

This is consistent with our observations. While on the first days after start of ECMO therapy the differences between the survivors and the non-survivors are far from clear, a clear difference is already measurable on day 10, although the event of death is still several weeks away in a large proportion of the non-survivors (see Figure 3). This is the reason why we used day 10 to calculate our model. Conversely, at the time start of ECMO therapy, the patients did not differ measurably with respect to the parameters typically reflecting disease severity.

**Reviewer #2:**

**1. Important work to determine the factors that most correlate with poor outcomes among patients placed on VVECMO. Work needs to be done to solidify eligibility criteria for VVECMO - especially in COVID-19 patients. The fact that age and platelet count were significant is helpful information.**

Thank you for this feedback. The intention for initiating this study was to generate further data to make meaningful decisions about which COVID-19 patients should receive therapy with VV ECMO.

**2. The manuscript is a little complicated for me (as a clinician) – I would recommend review by a biostatistician.**

The team of authors consists of experienced intensive care physicians, but also two scientists with appropriate mathematical training (Bärbel Kieninger, Bernd Salzberger). Bärbel Kieninger was responsible for the statistical calculations and developed the mathematical models presented in the manuscript.

**3. I would encourage the investigators to simplify the take home message in the discussion and how that take home message fits into the existing literature. For instance, if age and platelet count are associated with the worst outcomes, is this finding in-line with existing research (e.g. is the age range similar?).**

Our manuscript was originally submitted in August 2022. In the meantime, further studies on ECMO therapy in COVID-19 patients have been published. We performed a new literature search and found in addition to the study published by Bergman et al. three further papers that also showed a correlation between older age and higher probability of death. The mean age of patients included in these studies was basically in a similar range to the age of the patients in our study (our study: survivors 52.6 year, non-survivors 57.4 years; Bergman et al.: survivors 49.1 years, non-survivors 55.5 years; Joshi et al: survivors 46.5 years, non-survivors 51.8 years; Raff et al.: survivors 40.6 years, non-survivors 53.1 years; Hettlich et al.: survivors 47.9 years, non-survivors 56.7 years).

In addition, a study was published in the meantime in which significantly lower platelet counts were measured in COVID-19 patients with ECMO therapy in agreement with our results in non-survivors.

We have included the results of these studies in the discussion (line 332-333, line 350-352) and added the corresponding sources in the references.

We have tried to make the take home message in the Conclusion easier to understand and have reformulated it accordingly (line 391-403):

*Before initiation of ECMO therapy, subsequent survivors and non-survivors differed primarily in age and platelet count. A linear regression model could be calculated. By entering the patient's age and platelet count into this formula, a numerical value is obtained that allows estimation of the prognosis in terms of survival or death at ICU. This may provide guidance in deciding whether to begin VV ECMO therapy.*

*At 10 days after initiation of VV ECMO therapy, there were highly significant differences in age, platelet count, average blood pH, minimum paO2, and average pump flow in the later survivors and non-survivors. Again, a linear regression model could be calculated and cutoff values could be derived to estimate the prognosis with high probability. If there is any doubt as to whether it is appropriate to continue intensive care therapy, this can provide valuable additional information.*

*Because of the limited number of patients, however, the models need to be tested in a larger patient collective.*

**4. I could use some clarification of how the "cut off" values were determined - if Youden's index? (Or something similar?)**

For the calculation of the first model (model from the initial known parameters before VV ECMO), the value that mathematically best separated the 2 groups was chosen as cutoff, which corresponds to the maximization of the Youden index in the present data set.

The ROC curve in the second model (model at day 10) shows a peculiarity in that it first runs very close to the y-axis (very high specificity) and then in the later course runs parallel to the x-axis at a sensitivity of 1. Because of this shape, it is convenient to define 2 cutoffs, each of which keeps one of the classification errors false-positive and false-negative low, but at the same time satisfies the condition that the resulting subgroups must also contain a significant proportion of the patient cohort. The two cutoffs result in three patient groups, one with very high mortality and the second with very low mortality. For these two groups, the model is able to estimate the outcome in our cohort with high probability. However, for the third resulting group, no reasonable estimate of the outcome is possible. This method with two cutoffs seems to be much more helpful in clinical practice than if only one cutoff had been generated by optimizing both classification errors simultaneously.
